# Super-enhancer–driven EFNA1 fuels tumor progression in cervical cancer via the FOSL2-Src/AKT/STAT3 axis

**DOI:** 10.1172/JCI177599

**Published:** 2025-02-18

**Authors:** Shu-Qiang Liu, Xi-Xi Cheng, Shuai He, Tao Xia, Yi-Qi Li, Wan Peng, Ya-Qing Zhou, Zi-Hao Xu, Mi-Si He, Yang Liu, Pan-Pan Wei, Song-Hua Yuan, Chang Liu, Shu-Lan Sun, Dong-Ling Zou, Min Zheng, Chun-Yan Lan, Chun-Ling Luo, Jin-Xin Bei

**Affiliations:** 1 State Key Laboratory of Oncology in South China, Guangdong Provincial Clinical Research Center for Cancer, Collaborative Innovation Center for Cancer Medicine, Sun Yat-sen University Cancer Center, Guangzhou, China.; 2 Cancer Hospital of China Medical University, Liaoning Cancer Hospital & Institute, Shenyang, China.; 3 Department of Experimental Research, Sun Yat-sen University Cancer Center, Guangzhou, China.; 4 Department of Gynecologic Oncology, Chongqing University Cancer Hospital & Chongqing Cancer Institute & Chongqing Cancer Hospital, Chongqing, China.; 5 Department of Gynecology, The First People’s Hospital of Foshan, Foshan, China.; 6 Department of Medical Oncology, National Cancer Centre Singapore, Singapore.; 7 Sun Yat-sen University Institute of Advanced Studies Hong Kong, Science Park, Hong Kong, China.; 8 Department of Clinical Oncology, School of Clinical Medicine, Li Ka Shing Faculty of Medicine, University of Hong Kong, Hong Kong, China.

**Keywords:** Cell biology, Oncology, Cervical cancer, Epigenetics

## Abstract

Super-enhancers (SEs) are expansive *cis*-regulatory elements known for amplifying oncogene expression across various cancers. However, their role in cervical cancer (CC), a remarkable global malignancy affecting women, remains underexplored. Here we applied integrated epigenomic and transcriptomic profiling to delineate the distinct SE landscape in CC by analyzing paired tumor and normal tissues. Our study identifies a tumor-specific SE at the *EFNA1* locus that drives EFNA1 expression in CC. Mechanically, the *EFNA1-SE* region contains consensus sequences for the transcription factor FOSL2, whose knockdown markedly suppressed luciferase activity and diminished H3K27ac enrichment within the SE region. Functional analyses further underlined EFNA1’s oncogenic role in CC, linking its overexpression to poor patient outcomes. EFNA1 knockdown strikingly reduced CC cell proliferation, migration, and tumor growth. Moreover, EFNA1 *cis*-interacted with its receptor EphA2, leading to decreased EphA2 tyrosine phosphorylation and subsequent activation of the Src/AKT/STAT3 forward signaling pathway. Inhibition of this pathway with specific inhibitors substantially attenuated the tumorigenic capacity of EFNA1-overexpressing CC cells in both in vitro and in vivo models. Collectively, our study unveils the critical role of SEs in promoting tumor progression through the FOSL2-EFNA1-EphA2-Src/AKT/STAT3 axis, providing new prognostic and therapeutic avenues for CC patients.

## Abstract 2

<strong>S</strong><strong>uper-enhancer</strong><strong>-d</strong><strong>riven</strong> <strong>EFNA1 fuels</strong><strong> tumor progression in c</strong><strong>ervical cancer</strong>

## Introduction

Cervical cancer (CC) remains a remarkable global health challenge, ranking as the fourth most prevalent cancer and the fourth leading cause of cancer-related mortality among women worldwide (1, 2). Multiple factors contribute to the development of CC, including infections by high-risk human papillomavirus (HPV) subtypes (3), genetic susceptibility (4), and environmental risk exposures (5). While radical surgery or chemoradiotherapy has shown success in treating early-stage CC, late-stage diagnosis often results in poor prognosis, with a 5-year survival rate as low as 16.5% for patients with recurrent or metastatic disease (6). Therefore, a deeper understanding of the molecular mechanisms driving cervical carcinogenesis is essential for developing more effective diagnostic and therapeutic strategies.

Super-enhancers (SEs) are clusters of enhancer regions characterized by high densities of enhancer-associated histone modifications, such as histone H3 lysine 27 acetylation (H3K27ac) and histone H3 lysine 4 mono-methylation (H3K4me1), along with the binding of transcriptional coactivators and mediator complexes (7). These *cis*-regulatory elements play a crucial role in regulating the transcription of genes essential for cell identity and function (8–11). Recent evidence has linked SEs to the regulation of oncogenes in various cancers, including CC (12–15). Aberrant SEs have been implicated in the development and progression of several malignancies, such as neuroblastoma (16), medulloblastoma (17), breast (18), esophageal (19), gastric cancers (15, 20), pancreatic ductal adenocarcinoma (21), and melanoma (22). In the context of CC, SEs have been associated with HPV integration and the activations of oncogenic pathways involving EGFR and c-MET (23). However, most of these insights have been derived from commercial cell lines, which might not represent the epigenetic landscapes of primary tumors owing to technological challenges and limited access to clinical specimens. Notably, substantial differences exist between the epigenetic profiles of primary tumors and cell lines (17, 24), highlighting the urgent need for comprehensive exploration of the genome-wide SE landscape and understanding of their regulatory mechanisms in CC tumors.

In this study, we conducted integrated chromatin immunoprecipitation sequencing (ChIP-Seq) and transcriptome analyses to map the tumor-specific SE landscape in CC biopsies. Our analyses identify *EFNA1* as an SE-driven gene, modulated by the interaction of FOSL2 with the SE domains of *EFNA1*. Further investigations reveal that EFNA1 plays a tumorigenic role in CC via interacting with its receptor EphA2, leading to the activation of the Src/AKT/STAT3 signaling pathway. These findings underscore the pivotal role of SEs and their associated genes in the malignant progression of CC. Understanding the molecular mechanisms behind SE-mediated oncogenesis in CC holds vital promise for the development of targeted therapies and improved patient outcomes.

## Results

### Characterization of tumor-specific SEs and SE-regulated genes in CC.

**Figure 1:**
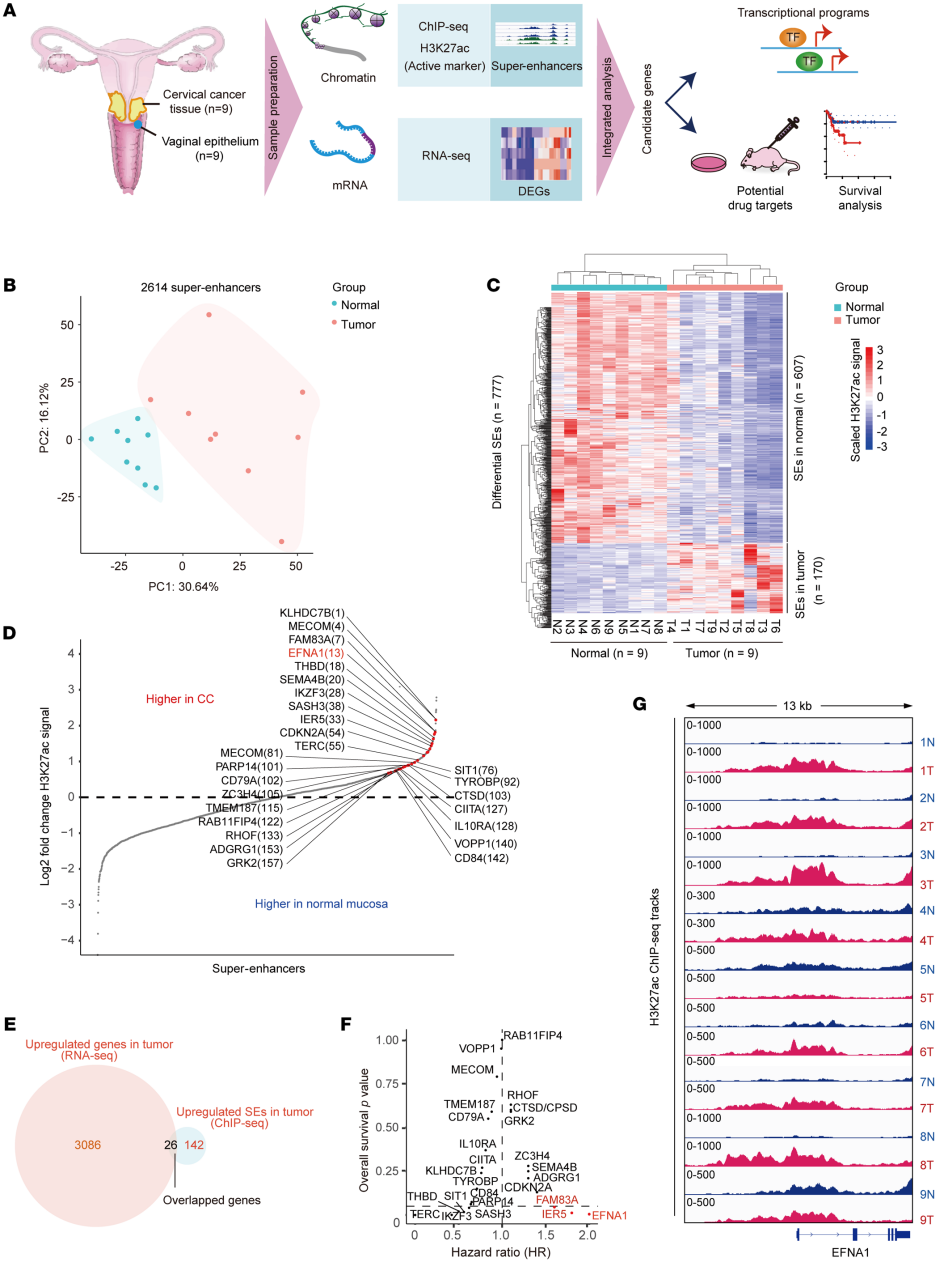
Global landscape of SEs in CC. (**A**) Graphical overview of the study design. (**B**) Principal component analysis (PCA) of H3K27ac levels from 2,614 SEs in 9 CC samples and their corresponding normal tissues. Tumor (red) and normal samples (blue) are represented by circles. (**C**) Heatmap illustrating differential SE activity in the 9 CC samples compared with their paired normal tissues. H3K27ac signals are raw-scaled, with the color spectrum ranging from red (high intensity) to blue (low intensity), indicating H3K27ac signal intensity. (**D**) Visualization of H3K27ac signals across 2,614 SEs. The *x* axis represents individual SEs, while the *y* axis portrays the log_2_ fold change in H3K27ac signals in CC relative to matched normal tissues. Red dots highlight 26 genes upregulated in CC compared with normal tissues that are considered potential SE targets. Numbers in parentheses indicate the rank order based on the fold increase of SEs in CC relative to normal counterparts. (**E**) Venn diagram illustrating the overlap between genes with upregulated expression in tumor and those targeted by elevated SEs in CC. (**F**) Scatterplot showing survival analysis for the 26 SE-targeted genes identified in **E**. The *x* axis represents hazard ratio (HR), and the *y* axis represents *P* values. Horizontal dashed line represents *P* = 0.05, and vertical dashed line represents HR = 1. Genes marked in red are upregulated and have a significant association with a poorer overall survival rate in CC patients. Patients were divided into 2 groups based on the median expression level of each gene. The statistical significance of differences between the 2 groups was assessed using the log-rank test. (**G**) H3K27ac ChIP-Seq signals mapped proximate to the EFNA1 locus in CC (T) and NOR samples (N).

To investigate the role of SEs and the epigenetic regulation in CC tumorigenesis, we conducted a comprehensive analysis of genome-wide H3K27ac occupancy using both ChIP-Seq and RNA sequencing (RNA-Seq) across 9 pairs of CC tumors and their matched normal tissues (NOR) (Figure 1A, Supplemental Figure 1, and Supplemental Table 1; supplemental material available online with this article; https://doi.org/10.1172/JCI177599DS1). After applying stringent bioinformatic filtering (Supplemental Figure 2, A–C), we identified an average of 54,140 H3K27ac signal peaks per sample at a genome-wide level (Supplemental Figure 2D and Supplemental Figure 3A). Notably, most of these signals were localized in promoter and intron regions within the CC tumor samples (Supplemental Figure 3B), consistent with patterns observed in other cancers (25, 26). Using the rank ordering of super-enhancers (ROSE) algorithm (11), we identified 2,614 SEs distributed across both CC and NOR samples. Principal component analysis (PCA) revealed distinct SE distribution patterns between tumor and normal samples, indicating substantial SE dynamics during CC development (Figure 1B). Further comparison between CC and NOR samples revealed 777 differentially activated SEs, including 170 more active in CC and 607 predominant in NOR samples (Figure 1C and Supplemental Table 2).

The ROSE algorithm also revealed SE-associated genes based on their proximity to SE regions (Figure 1D and Supplemental Table 2). Considering the propensities of SEs to facilitate target gene expression, we next examined the expression patterns of these SE-associated genes in CC using our RNA-Seq data (Supplemental Figure 2, E and F, Supplemental Figure 4, A and B, and Supplemental Table 3). Gene set enrichment analysis validated that genes associated with activated SEs (*N* = 170) in tumors exhibited increased expression, while those linked to suppressed SEs (*N* = 607) showed diminished expression, underscoring the regulatory function of SEs on these genes (Supplemental Figure 4, C and D). These findings highlight a pivotal role of SEs in mediating CC development through their target genes.

To further identify key SE-regulated genes involved in CC tumorigenesis, we intersected these differential SE-associated genes with differentially expressed genes (DEGs) between CC and normal samples (Supplemental Figure 4, B and E). This analysis spotlighted 26 SE-regulated genes with increased expression and 167 with decreased expression in CC compared with NOR (Figure 1E and Supplemental Figure 4E). Among the 26 upregulated SE-regulated genes, survival analysis revealed that 3 genes — *EFNA1*, *IER5*, and *FAM83A* — were significantly associated with poorer overall survival in CC patients derived from the GEPIA2 database (27) (*n* = 292; Figure 1F). Notably, *EFNA1* demonstrated the most significant association (*P* = 0.0047) and the highest hazard ratio (HR; Figure 1F), positioning it as the most promising candidate. Additionally, *EFNA1* showed a modest association with disease-free survival (*P* = 0.077), with an HR greater than 1.5 among the 3 genes (Supplemental Figure 4F). Subsequent transcriptome analysis corroborated elevated *EFNA1* expression in CC compared with NOR samples (Supplemental Figure 4G). These findings strongly suggest that SE-driven regulation of *EFNA1* plays a crucial role in CC progression.

### Identification of a cis-regulatory SE at the EFNA1 locus in CC.

**Figure 2:**
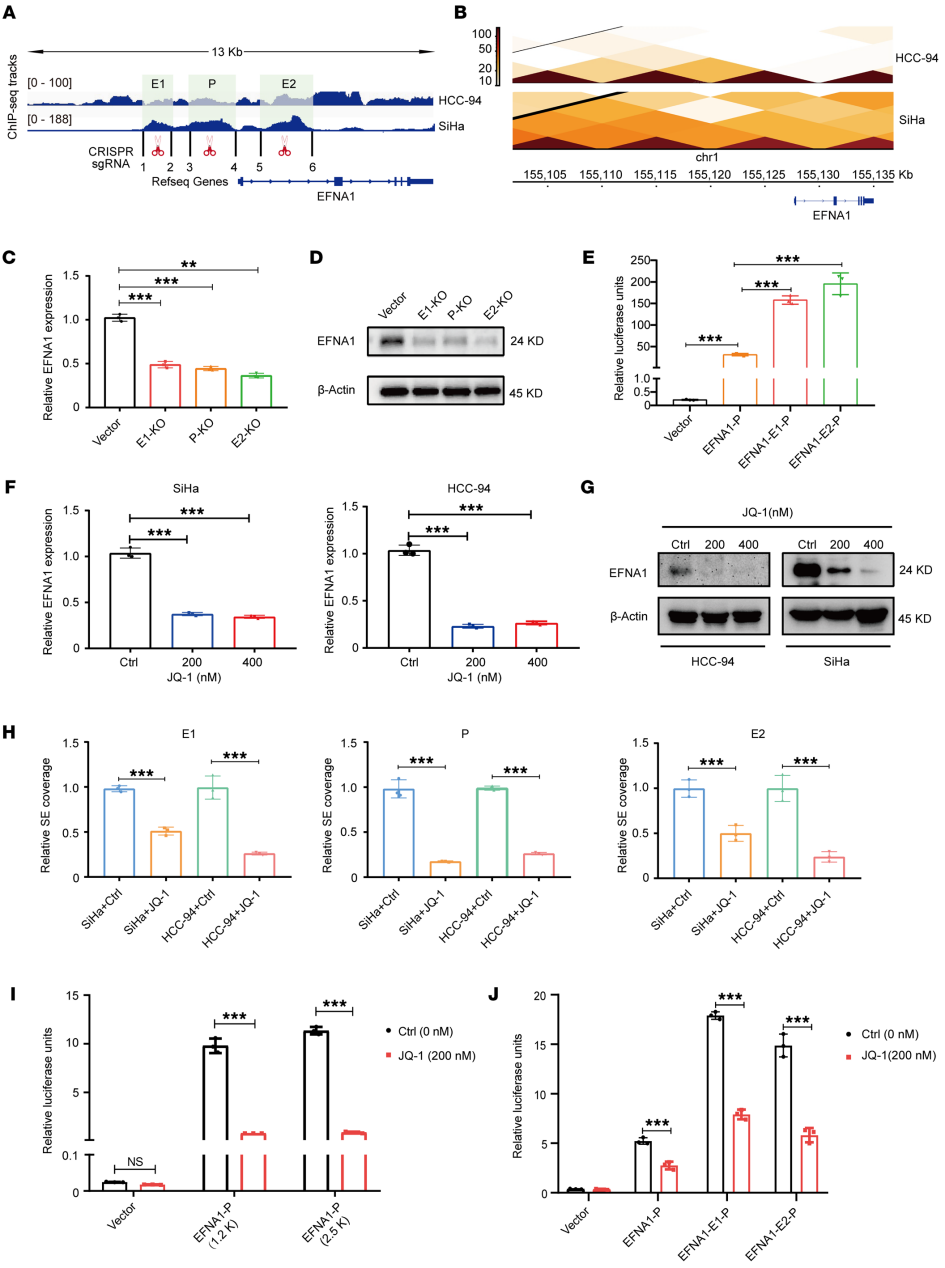
Identification of *EFNA1* as an SE-driven gene in CC. (**A**) Schematic diagram illustrating H3K27ac ChIP-Seq signals proximate to the *EFNA1* locus in SiHa and HCC-94 cells and the CRISPR/Cas9–mediated deletions targeting *EFNA1*-SEs. (**B**) Topologically associated domain (TAD) regions at the *EFNA1* locus, predicted based on Hi-C data from CC cells. The heatmap color gradient, from white to red, represents the interaction intensity between the SE and the *EFNA1* promoter region, ranging from low to high. (**C** and **D**) Analysis of *EFNA1* expression in SiHa cells following *EFNA1*-SE deletions, including E1 (E1-KO), E2 (E2-KO), and promoter (P-KO) regions. mRNA levels were measured by quantitative reverse transcription–PCR (qRT-PCR) (**C**), and protein levels were assessed by Western blot (**D**), with β-actin serving as the internal control. (**E**) Dual-luciferase reporter assays in HEK293T cells assessing the enhancer activities of *EFNA1* promoter (EFNA1-P) and *EFNA1*-SEs (EFNA1-E1-P, EFNA1-E2-P). (**F** and **G**) *EFNA1* expression in CC cells treated with various concentrations of JQ1. qRT-PCR results (**F**) and Western blot results (**G**) are shown, with vehicle-treated cells as the control. (**H**) qPCR assay showing H3K27ac enrichments at the *EFNA1* promoter (P) and SE regions (E1, E2) from ChIP assays in CC cells. The ChIP assays were conducted using H3K27ac antibodies, in cells treated either with or without 200 nM JQ1 for 24 hours. (**I** and **J**) Luciferase reporter assays assessing the transcriptional activity of the *EFNA1* promoter (1.2 K and 2.5 K) (**I**) and the combined *EFNA1* promoter and SE regions, *EFNA1*-P-SEs (**J**), in HEK293T cells treated with 200 nM JQ1 or vehicle control for 24 hours (EFNA1-E1-P, EFNA1-E2-P). Data are presented as mean ± SD, with *n* = 3 replicates. Between-group comparisons: 1-way ANOVA test. Significant *P* values: **P < 0.01, ***P < 0.001.

Our ChIP-Seq analysis, using the ROSE algorithm, identified 2 enhancer constituents (E1 and E2) within the *EFNA1-SE* locus. CC samples exhibited a remarkable enrichment of H3K27ac occupancy at these sites compared with their paired NOR samples (Figure 1G), which was further corroborated by ChIP–quantitative PCR (qPCR) assays (Supplemental Figure 5A). Furthermore, we observed a strong correlation between H3K27ac deposition at the *EFNA1-SE* locus and EFNA1 transcription (Supplemental Figure 5B). ChIP-Seq analysis of CC cell lines using H3K27ac antibodies, combined with ChIP-PCR analysis using H3K4me1 and BRD4 antibodies (well-established markers of enhancers), confirmed the presence of the SE region within *EFNA1*, consistent with the pattern observed in the aforementioned CC samples (Figure 2A and Supplemental Figure 5, C and D). Moreover, Hi-C assay further confirmed a direct interaction between the *EFNA1-SE* locus and the *EFNA1* promoter in SiHa and HCC-94 cells (Figure 2B). Collectively, these findings strongly suggest that the SE locus plays a critical role in regulating *EFNA1* transcription.

To further verify the regulatory function of the *EFNA1-SE*, we used CRISPR/Cas9 technology to delete each of the endogenous constituents (E1, E2, and promoter) within the *EFNA1-SE* in SiHa cells (Figure 2A and Supplemental Figure 5E). The deletion of these SE constituents resulted in a significant reduction in *EFNA1* expression at both the mRNA and protein levels (Figure 2, C and D). Conversely, introducing luciferase reporter plasmids containing the exogenous SE constituents (*EFNA1*-P-E1~2) led to higher luciferase activity in HEK293T cells (Figure 2E), affirming the pivotal regulatory role of the SE locus in modulating *EFNA1* expression. Given that BRD4 is known to interact with H3K27ac at SEs to drive transcriptional activation and elongation (28), we explored whether the SE-driven regulation of *EFNA1* was dependent on BRD4. Treatment with JQ1, a small-molecule inhibitor blocking the binding of BRD4 to H3K27ac, resulted in a decline in EFNA1 expression at both mRNA and protein levels in SiHa and HCC-94 cells (Figure 2, F and G). ChIP-qPCR assays further revealed a reduction in H3K27ac occupancy at the SE regions (E1 and E2) following JQ1 treatment in these two CC cell lines (Figure 2H). Additionally, luciferase reporter assays demonstrated that JQ1 treatment significantly impaired the regulatory capacity of SE constituents over the *EFNA1* promoter, thereby hindering the transcriptional activity of the *EFNA1* promoter (Figure 2, I and J). Collectively, these findings underscore the BRD4-dependent regulatory function of SEs in controlling *EFNA1* expression.

To explore whether *EFNA1-SE* regulation is a common feature across various cancer types, we conducted a pan-cancer analysis encompassing 24 primary cancer types. Interestingly, only a fraction (~25%) of these cancers manifested the *EFNA1-SE* signature, with detection rates ranging from 5% to 39%, notably lower than the 56% prevalence observed in CC samples (Supplemental Figure 5F). Subsequent transcriptome analyses of tumors with an activated *EFNA1-SE* (detected ratio >1%) revealed that *EFNA1* upregulation was predominantly observed in CC and colon adenocarcinoma (COAD) tumors (Supplemental Figure 4G and Supplemental Figure 5G), highlighting a tumor-specific SE-driven activation of *EFNA1* in certain cancer types.

### FOSL2 engagement at the EFNA1-SE facilitates EFNA1 transcription.

**Figure 3:**
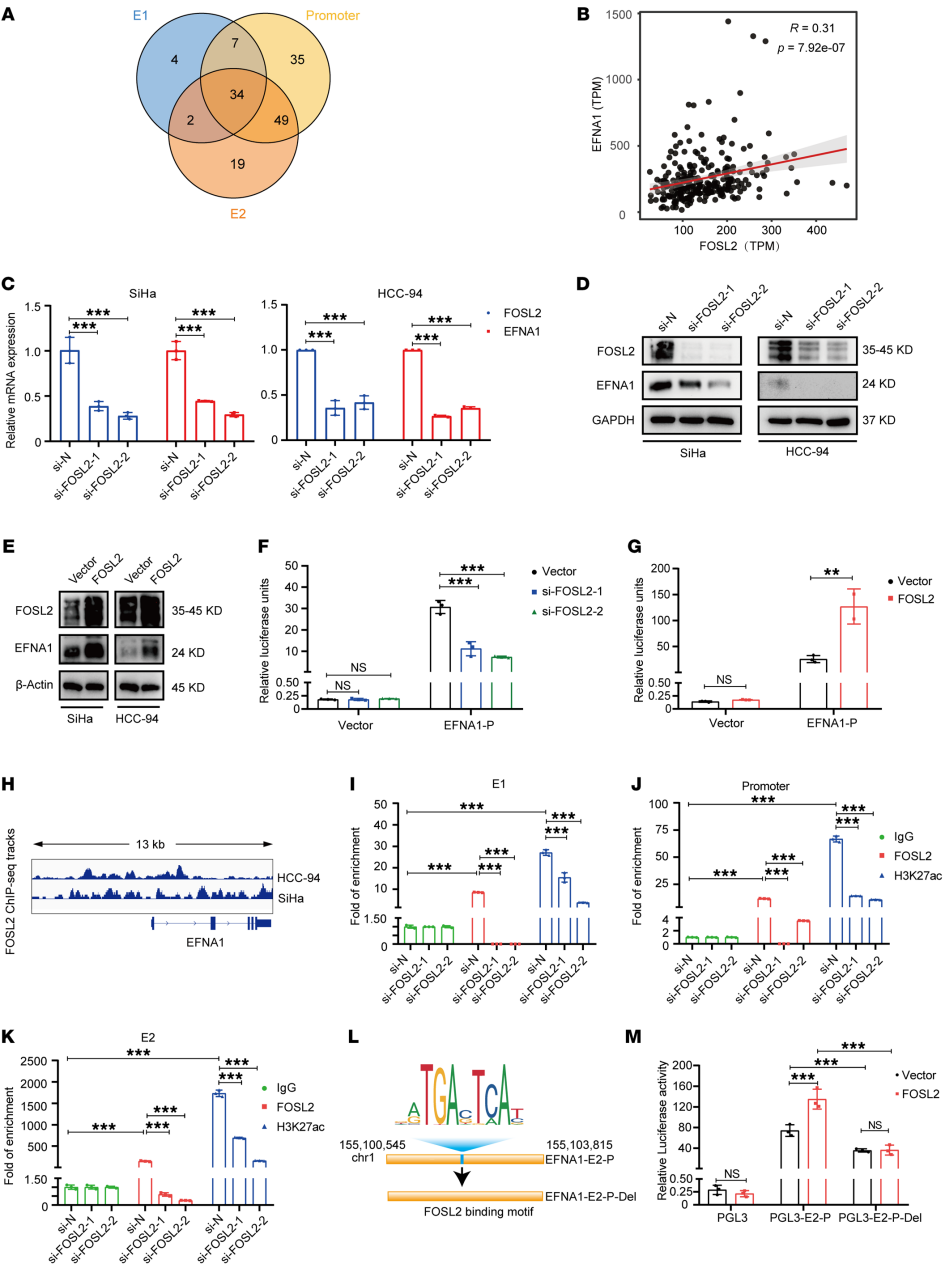
FOSL2 regulates *EFNA1* transcription through binding to SE regions. (**A**) Venn diagram presenting potential transcription factors binding to the SE regions (E1 and E2) and the core promoter region of *EFNA1*. (**B**) Pearson’s correlation analysis showing the positive correlation between EFNA1 and FOSL2 expression in CC samples from the TCGA database (27). TPM, transcripts per million. (**C**) Expression of *FOSL2* and *EFNA1* in SiHa and HCC-94 cells transiently transfected with siRNAs targeting *FOSL2* or control siRNA. mRNA expression was determined using qRT-PCR. (**D** and **E**) Western blotting showing the protein expression levels of FOSL2 and EFNA1 following FOSL2 knockdown (**D**) or overexpression (**E**). (**F** and **G**) Luciferase reporter assay showing *EFNA1* promoter activity in HEK293T cells with FOSL2 knockdown (**F**) or overexpression (**G**). (**H**) Gene tracks showing FOSL2 ChIP-Seq occupancy at the *EFNA1* loci in SiHa and HCC-94 cell lines. The *x* axis shows genomic position, and the *y* axis shows ChIP-Seq occupancy signal in reads per million mapped reads per base pair (rpm/bp). (**I**–**K**) qPCR assay showing FOSL2 and H3K27ac enrichments at the *EFNA1* core promoter and SE regions from ChIP assay in HCC-94 cells with FOSL2 and H3K27ac antibodies. Data are presented as mean ± SD from 3 independent experiments. (**L**) Schematic diagram showing the *EFNA1* E2 region (from chr1:155,100,545–155,103,815) with an FOSL2 binding motif (from chr1:155,101,975–155,101,985). (**M**) Luciferase activity of the indicated plasmids in HEK293T cells. After 48 hours of transfection of specified plasmid, luciferase activity was determined and normalized to pRL-TK luciferase activity. Data are presented as mean ± SD across *n* = 3 replicates. Statistical analysis was performed using Pearson’s correlation test in **B**, 1-way ANOVA test in **C**, **F**, **G**, **I**–**K**, and **M**. Significant *P* values: **P < 0.01, ***P < 0.001.

To uncover the regulatory mechanisms driving the transcriptional activation of the *EFNA1-SE*, we screened transcription factors (TFs) that might potentially bind to the *EFNA1-SE* locus using ChIP-Seq data from the ENCODE project (29). We identified 150 TFs with potential binding sites dispersed across the component enhancer constituents (E1 and E2) and the core promoter area at the *EFNA1* locus. Among these, 34 TFs exhibited potential binding across the entire SE region (Figure 3A). To further narrow down key TFs, we conducted a correlation analysis between the expression of these 34 TFs and *EFNA1* in CC tumors using transcriptomic data from The Cancer Genome Atlas (TCGA) database. Notably, *FOSL2* emerged as the top candidate, showing the strongest correlation with *EFNA1* expression (Figure 3B and Supplemental Table 4). To validate the regulatory role of *FOSL2* in *EFNA1* expression, we performed FOSL2 knockdown in SiHa and HCC-94 cells, which resulted in a significant decrease in EFNA1 expression at both the mRNA and protein levels (Figure 3, C and D). Conversely, FOSL2 overexpression led to an increase in EFNA1 expression (Figure 3E), affirming FOSL2’s regulatory impact on *EFNA1*.

To further elucidate the mechanism by which FOSL2 regulates EFNA1, we performed luciferase reporter assays. FOSL2 knockdown significantly suppressed the luciferase activity of the *EFNA1* promoter in HEK293T cells (Figure 3F), while FOSL2 overexpression significantly enhanced it (Figure 3G). This suggests that FOSL2 directly influences *EFNA1* transcription. Additionally, ChIP-Seq analysis demonstrated that FOSL2 directly bound to the *EFNA1* enhancer region (Figure 3H). Complementary ChIP-qPCR assays further revealed the coexistence of FOSL2 and H3K27ac at the core promoter and adjacent SE constituents of the *EFNA1* locus in CC cells (Figure 3, I–K). Moreover, FOSL2 knockdown led to reduced H3K27ac deposition at the *EFNA1-SE* (Figure 3, I–K). Further analysis using the JASPAR database identified potential FOSL2 binding motifs within the *EFNA1* E1 and E2 regions (Figure 3L and Supplemental Figure 6A). Subsequent luciferase assays showed that the increased luciferase activity in the *EFNA1-SE* constituents due to FOSL2 overexpression was significantly abolished when the binding motifs were removed (Figure 3M and Supplemental Figure 6B). Intriguingly, coimmunoprecipitation (co-IP) assays demonstrated a direct interaction between FOSL2 and H3K27ac (Supplemental Figure 6, C and D), strongly suggesting that FOSL2, in conjunction with H3K27ac, activates *EFNA1* transcription at the SE in CC cells.

### Oncogenic role of EFNA1 in CC.

**Figure 4:**
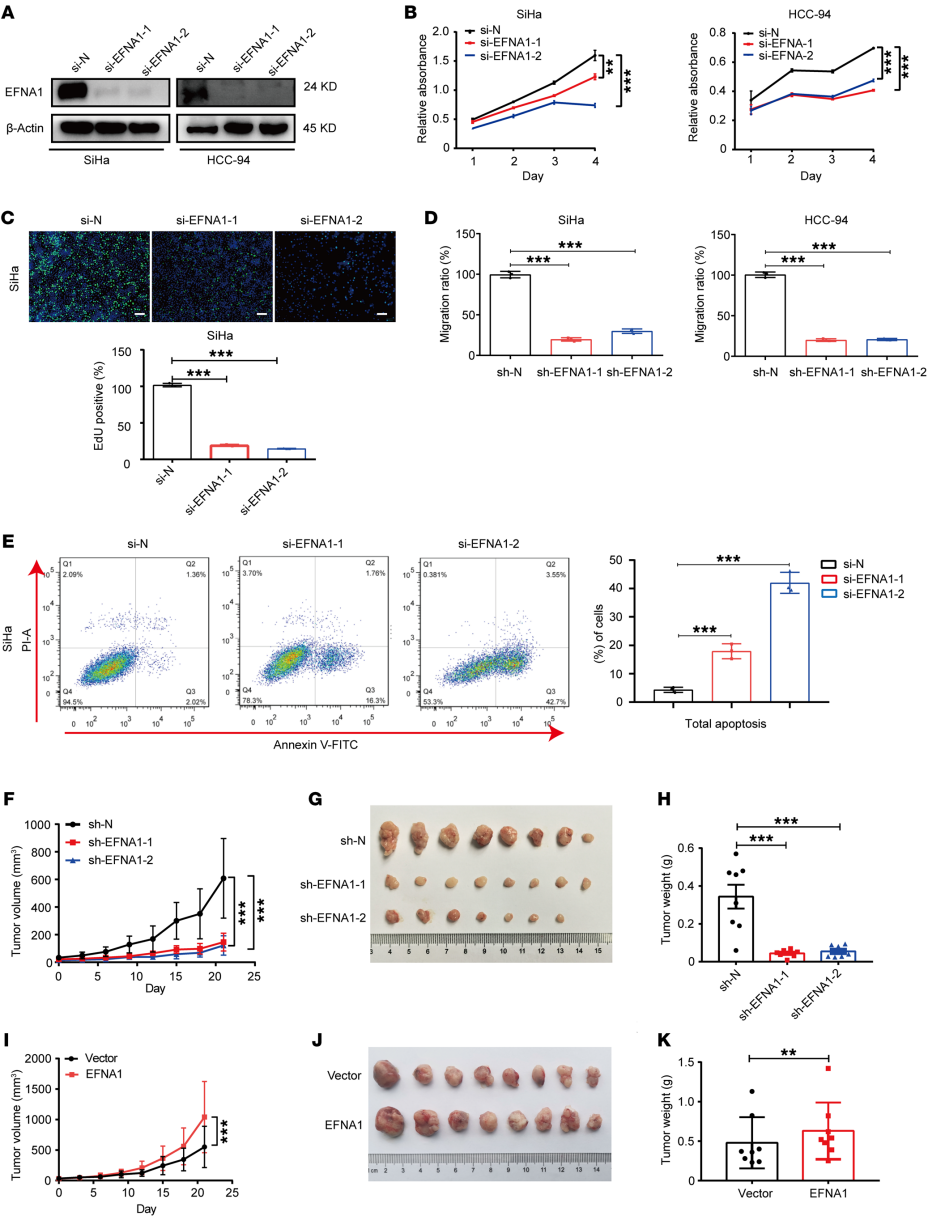
*EFNA1* acts as an oncogene in CC. (**A**) Western blot assay showing the knockdown efficiency of *EFNA1* in SiHa and HCC-94 cells transfected with siRNAs specifically targeting *EFNA1* or control siRNA. β-Actin serves as a loading control. (**B**) CCK-8 assay showing the cell growth rate of cells described in **A**. Absorbance from day 1 to day 4 was normalized to day 0 values. (**C**) Representative images of EdU staining in SiHa cells from **A**. The corresponding statistical analysis is presented at the bottom. (**D**) Statistical results of Transwell assay showing the migration ability of SiHa and HCC-94 cells infected with lentivirus expressing EFNA1 shRNAs or control shRNA. (**E**) Representative images of flow cytometry analysis of apoptosis in SiHa cells described above, using annexin V–FITC/propidium iodide staining. Quantification is presented on the right. (**F**–**K**) Tumorigenesis measurements in nude mice subcutaneously injected with SiHa cells expressing EFNA1 knockdown shRNA or control shRNA (**F**–**H**), and SiHa cells with stable EFNA1 overexpression or control vectors (**I**–**K**). Tumor volumes were measured (**F** and **I**), and tumors were photographed (**G** and **J**) and weighed (**H** and **K**) after mice were sacrificed. Scale bars: 100 μm. Between-group comparisons: 1-way ANOVA test. Significant *P* values: **P < 0.01, ***P < 0.001.

Considering the unique presence of *EFNA1-SE* and the upregulated *EFNA1* expression in CC, we hypothesized that *EFNA1* may play an oncogenic role in CC. To verify this, we first conducted EFNA1 knockdown in the CC cell lines SiHa and HCC-94 (Figure 4A). Subsequent Cell Counting Kit-8 (CCK-8) and EdU staining assays revealed that EFNA1 knockdown significantly inhibited cell proliferation (Figure 4, B and C, and Supplemental Figure 7A). Furthermore, EFNA1 knockdown led to marked reduction in the migration and invasion abilities of CC cells (Figure 4D and Supplemental Figure 7, B and C). These findings were further supported by the deletion of *EFNA1-SE*s (E1 and E2), which resulted in attenuated EFNA1 expression and further suppression of proliferation and migration in CC cells (Supplemental Figure 7, D–F). Flow cytometry analysis revealed that EFNA1 knockdown obviously induced apoptosis and caused cell cycle arrest at the S phase in CC cells (Figure 4E and Supplemental Figure 8, A and B).

To further explore *EFNA1*’s tumorigenic function in vivo, we established a xenograft mouse model using SiHa cells expressing *EFNA1* shRNAs (sh-*EFNA1*-1, sh-*EFNA1*-2) or control shRNA (sh-N). Remarkably, EFNA1 knockdown led to significant inhibition of tumor growth, as evidenced by reductions in both tumor volume and weight in comparison with the control group (Figure 4, F–H). In support of this, Ki-67 immunostaining, a proliferative marker, demonstrated a sharp decline in Ki-67^+^ cells in the knockdown groups (Supplemental Figure 8, C and D). These in vitro and in vivo data strongly suggest that *EFNA1* is essential for the proliferation, migration, and tumor growth of CC cells.

To further corroborate the oncogenic role of *EFNA1* in CC, we also generated CC cell lines with exogenous EFNA1 overexpression (Supplemental Figure 9A). EFNA1 overexpression significantly promoted cell proliferation, as evidenced by cell growth curves, colony formation, and EdU staining assays (Supplemental Figure 9, B–D). Moreover, Transwell assays demonstrated a significant increase in migration and invasion potential upon EFNA1 overexpression (Supplemental Figure 9, E and F). Additionally, an in vivo xenograft model revealed that EFNA1 overexpression markedly promoted tumor growth in CC cells (Figure 4, I–K). Collectively, these findings underscore the oncogenic role of *EFNA1* in CC. In contrast, overexpression of either EFNA2 or EFNA5 significantly reduced cell proliferation and migration in vitro and tumor growth in vivo in comparison with control cells (Supplemental Figure 10), suggesting divergent biological roles among ephrinA family members.

### EFNA1 positively regulates the Src/AKT/STAT3 pathway.

**Figure 5:**
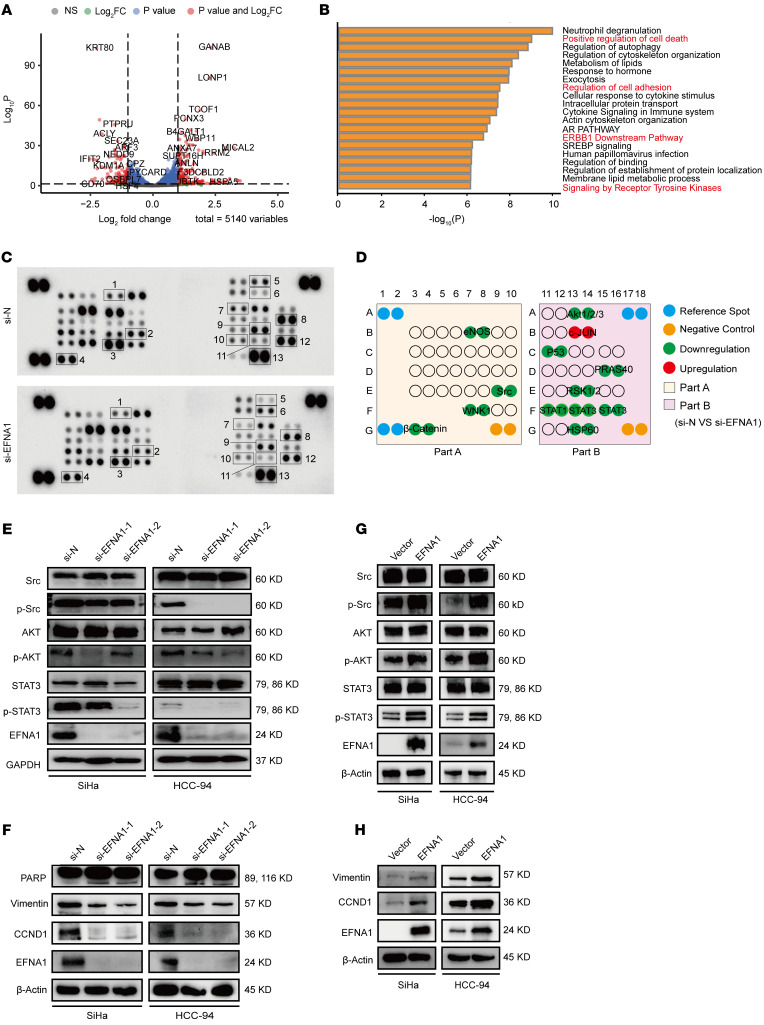
EFNA1 stimulates the Src/AKT/STAT3 signaling pathway. (**A**) Volcano plot illustrating the DEGs in HCC-94 cells following *EFNA1* knockdown relative to control cells, based on RNA-Seq analysis (FDR < 0.01). The *x* axis shows log_2_ fold change, and the *y* axis shows log_10_ *P*. (**B**) Top pathways affected by *EFNA1* knockdown as identified by Gene Ontology analysis for the DEGs as described in **A**. (**C** and **D**) Human phospho-kinase array results showing the phosphorylated proteins in SiHa cells transfected with EFNA1 siRNAs or control siRNA. Numbered boxes are highlighted targets and phosphorylation sites listed on the table at right (**D**). Red dots indicate upregulated phosphorylated proteins, while green dots represent downregulated ones (**D**). (**E**–**H**) Western blot assays showing protein levels of the Src/AKT/STAT3 pathway and its downstream genes in SiHa and HCC-94 cells following *EFNA1* knockdown (**E** and **F**) or overexpression (**G** and **H**). β-Actin or GAPDH serves as a loading control.

To further investigate the molecular mechanisms underlying EFNA1-mediated tumorigenesis, we conducted transcriptome analysis, revealing 394 DEGs influenced by EFNA1 knockdown in HCC-94 cells (Figure 5A). Gene Ontology analysis revealed that these DEGs were predominantly linked to cell death and adhesion processes (Figure 5B), aligning with *EFNA1*’s role in promoting the viability and motility of CC cells. Notably, EFNA1 knockdown also suppressed the receptor tyrosine kinases signaling pathway (Figure 5B), consistent with previous findings in other cancers (30, 31). To further delineate the phosphorylation pathways influenced by EFNA1, we performed human phosphorylated kinase arrays in SiHa cells. Strikingly, EFNA1 knockdown led to decreased phosphorylation levels of several essential players in the AKT and STAT3 signaling pathways (Figure 5, C and D), which were further validated through Western blot assays (Figure 5E). Additionally, Western blot assays demonstrated that EFNA1 knockdown markedly reduced the expression of upstream (p-Src) and downstream proteins (CCND1, vimentin) in the AKT/STAT3 pathway in CC cells (Figure 5, E and F). In contrast, EFNA1 overexpression resulted in a substantial upregulation of these proteins (Figure 5, G and H).

Subsequent transcriptomic analysis of CC tissues (*n* = 9) and their paired adjacent non-tumor tissues revealed a substantial upregulation of key genes involved in the Src/AKT/STAT3 pathway, along with elevated EFNA1 expression in tumor samples compared with normal tissues (Supplemental Figure 11A). Further validation using data from TCGA and single-cell transcriptomic profile of CC (32) consistently demonstrated robust activation of the Src/AKT/STAT3 pathway in tumors, particularly within malignant cells (Supplemental Figure 11, B and C). Collectively, these findings strongly support the activation of the Src/AKT/STAT3 pathway and position *EFNA1* as a critical activator of this signaling axis in CC.

### EphA2 mediates EFNA1’s signaling through cis-interaction.

**Figure 6:**
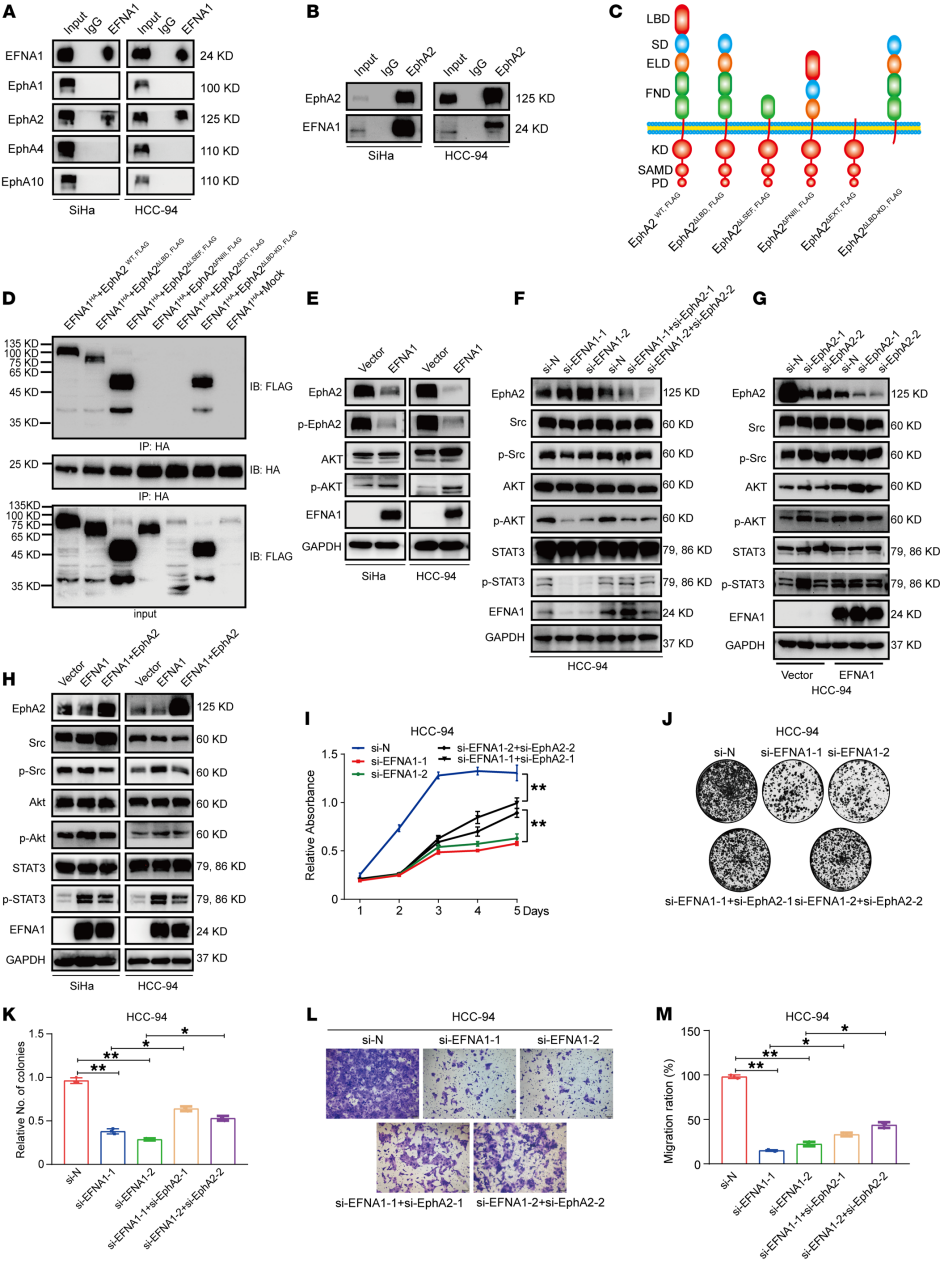
EphA2 mediates EFNA1 signals through *cis*-interaction with EFNA1. (**A** and **B**) Western blot analyses of immunoprecipitated products using anti-EFNA1 (**A**) or anti-EphA2 (**B**) antibodies in SiHa and HCC-94 cells. (**C**) Schematic representation of the domain structure of wild-type EphA2 and its mutants. LBD, ligand binding domain; SD, sushi domain; ELD, epidermal growth factor–like domain; FND, fibronectin type III domains; KD, kinase domain; SAMD, sterile-α-motif domain; PD, PDZ binding domain. (**D**) Co-IP assays in SiHa cells cotransfected with EFNA1-HA and either EphA2^WT,FLAG^, EphA2^ΔLCF2,FLAG^, EphA2^ΔLBD,FLAG^, EphA2^ΔEXT,FLAG^, EphA2^ΔFNIII^, or EphA2^ΔLBD-KD,FLAG^ plasmids. Immunoprecipitations were performed using anti-HA antibodies. (**E**) Western blot analysis of the total and phosphorylated protein levels of EphA2 and AKT in SiHa and HCC-94 cells with EFNA1 overexpression. GAPDH serves as a loading control. (**F** and **G**) Western blot analyses of EphA2 and downstream cascades in HCC-94 cells with concurrent EphA2 knockdown and EFNA1 knockdown (**F**) or EFNA1 overexpression (**G**). GAPDH serves as a loading control. (**H**) Western blot analysis of EphA2 and downstream cascades in HCC-94 cells with concurrent EphA2 and EFNA1 overexpression. GAPDH serves as a loading control. (**I**) CCK-8 assay for cell proliferation in SiHa and HCC-94 cells with *EFNA1* knockdown alone or combined knockdown of both *EFNA1* and EphA2. (**J** and **K**) Colony formation assay for cells described in **I**, with corresponding statistical data presented in **K**. (**L** and **M**) Representative images of Transwell assay showing the migration ability of cells described in **I**, with statistics shown on the right in **M**. Scale bars: 100 μm. Between-group comparisons: 1-way ANOVA test. Significant *P* values: *P <0.05, **P < 0.01.

Given that EFNA1 typically interacts with EphA receptors to initiate downstream signaling cascades (33), we sought to identify the specific EphA receptor involved with EFNA1 in CC cells. Transcriptome analysis revealed the expression of *EphA1*, *EphA2*, *EphA4*, and *EphA10*, but no other EphA receptors, in both SiHa and HCC-94 cell lines (Supplemental Figure 12). Co-IP assays further demonstrated that EFNA1 specifically interacted with EphA2, but not with other EphA receptors (Figure 6, A and B), identifying EphA2 as the primary receptor for EFNA1 in CC.

To determine whether EphA2 mediates EFNA1’s signaling through *cis*- or *trans*-interaction (34), we constructed a series of truncated EphA2 receptor mutants, each lacking a specific functional domain or domains (Figure 6C). Strikingly, both forward and reverse co-IP assays revealed that deletion of the FNIII domain alone (EphA2^ΔFNIII^) or the entire extracellular part (EphA2^ΔEXT^) completely abolished the interaction with EFNA1. In contrast, removal of other domains, including the ligand-binding domain (LBD), did not affect this interaction, indicating a *cis*-interaction between the 2 proteins mediated by the membrane-proximal FNIII domain of EphA2 (Figure 6D and Supplemental Figure 13).

We next elucidated whether EphA2 contributes to EFNA1-mediated activation of Src/AKT/STAT3 pathway. Intriguingly, EFNA1 overexpression led to a remarkable decrease in both the expression and phosphorylation of EphA2 (Figure 6E). Moreover, EphA2 knockdown notably increased the phosphorylation levels of Src, AKT, and STAT3 in CC cells independently of EFNA1 expression (Figure 6F). Importantly, the activation of the Src/AKT/STAT3 pathway induced by EFNA1 overexpression was substantially reversed by EphA2 overexpression (Figure 6, G and H). These findings suggest that EFNA1 activates this pathway by suppressing EphA2 expression and function. Subsequent functional studies confirmed that EphA2 knockdown remarkably counteracted the inhibitory effects on cell proliferation and migration caused by EFNA1 knockdown in CC cells (Figure 6, I–M), underscoring EphA2’s pivotal role in EFNA1-driven CC progression. Additionally, both forward and reverse IP experiments revealed no detectable interactions between EphA2 and EFNA2 or EFNA5 (Supplemental Figure 14), suggesting that other receptors and downstream pathways may mediate the tumor-suppressive roles of EFNA2 and EFNA5 (Supplemental Figure 10).

### EFNA1 drives tumor progression through Src/AKT/STAT3 pathway activation.

**Figure 7:**
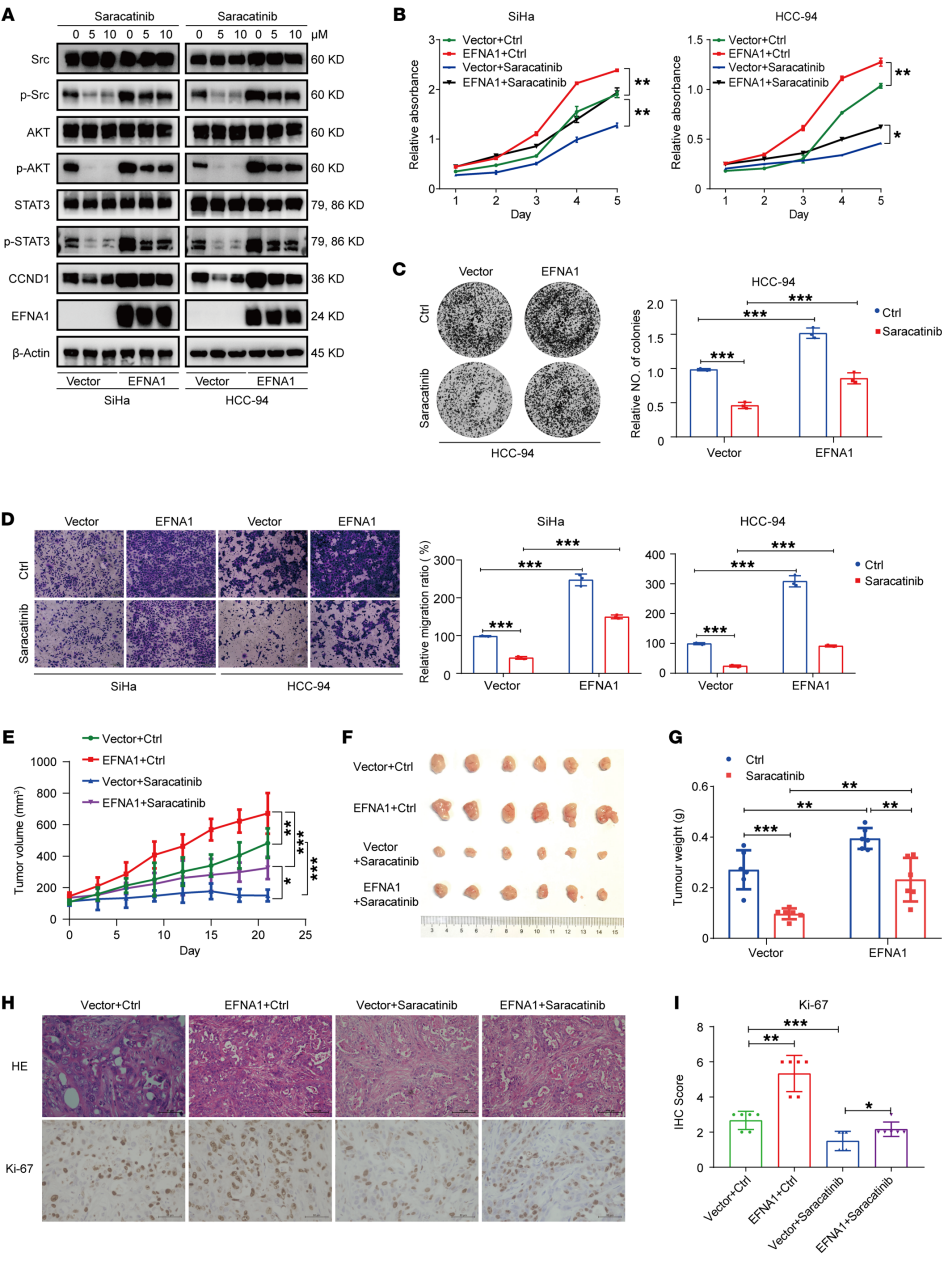
Saracatinib mitigates EFNA1-driven tumorigenesis in CC. (**A**) Western blot analyses of Src/AKT/STAT3 signaling pathway proteins and downstream genes in SiHa and HCC-94 cells with or without EFNA1 overexpression and saracatinib treatment. GAPDH serves as a loading control. (**B**) CCK-8 assay showing cell proliferation curves for cells described in **A**. (**C**) Colony formation for cells described in **A**, with corresponding statistical analysis on the right. (**D**) Representative images for Transwell assay showing migration ability of cells described in **A**, with statistical data indicated on the right. Scale bar: 200 μm. (**E**–**G**) Tumorigenesis measurements in nude mice subcutaneously injected with HCC-94 cells stably expressing EFNA1 or control vectors, and then treated with saracatinib or control vehicle. Tumor volumes were measured every 3 days (**E**). Tumors were photographed (**F**) and weighed (**G**) after the mice were sacrificed. (**H** and **I**) H&E staining results and IHC of Ki-67 in tumors described in **F**, with corresponding statistics presented on the right. Scale bars: 100 μm. Between-group comparisons: 1-way ANOVA test. Significant *P* values: *P < 0.05, **P < 0.01, ***P < 0.001.

To investigate the role of the Src/ATK/STAT3 pathway in mediating the tumorigenic function of EFNA1, we introduced a series of pathway-specific inhibitors, including saracatinib (Src inhibitor), MK2206 (AKT inhibitor), and Stattic (STAT3 inhibitor). Using in vitro CC cell assays, we found that the upregulated phosphorylation levels of Src and its downstream signaling pathways (AKT/STAT3), induced by EFNA1 overexpression, were significantly inhibited upon treatment with saracatinib (Figure 7A). Furthermore, saracatinib treatment markedly reduced the proliferation and migration abilities of CC cells overexpressing EFNA1 (Figure 7, B–D). Supporting these findings, in vivo xenograft models demonstrated that saracatinib treatment substantially diminished the pro-tumorigenic potential of CC cells overexpressing EFNA1 (Figure 7, E–G) and led to a reduced number of Ki-67^+^ proliferative cells in treated tumors compared with the control groups (Figure 7, H and I). Additionally, similar anti-proliferative and anti-migratory effects were observed with MK2206 and Stattic treatment, which also effectively suppressed phosphorylation within the AKT/STAT3 pathway (Supplemental Figures 15 and 16). These effects were consistently demonstrated in in vivo mouse models, where both inhibitors significantly restricted tumorigenesis in EFNA1-overexpressing CC cells (Supplemental Figures 15 and 16). Together, these observations strongly suggest that EFNA1 drives tumor progression primarily through activation of the Src/AKT/STAT3 signaling axis.

### Association of EFNA1 with poor survival in CC patients.

**Figure 8:**
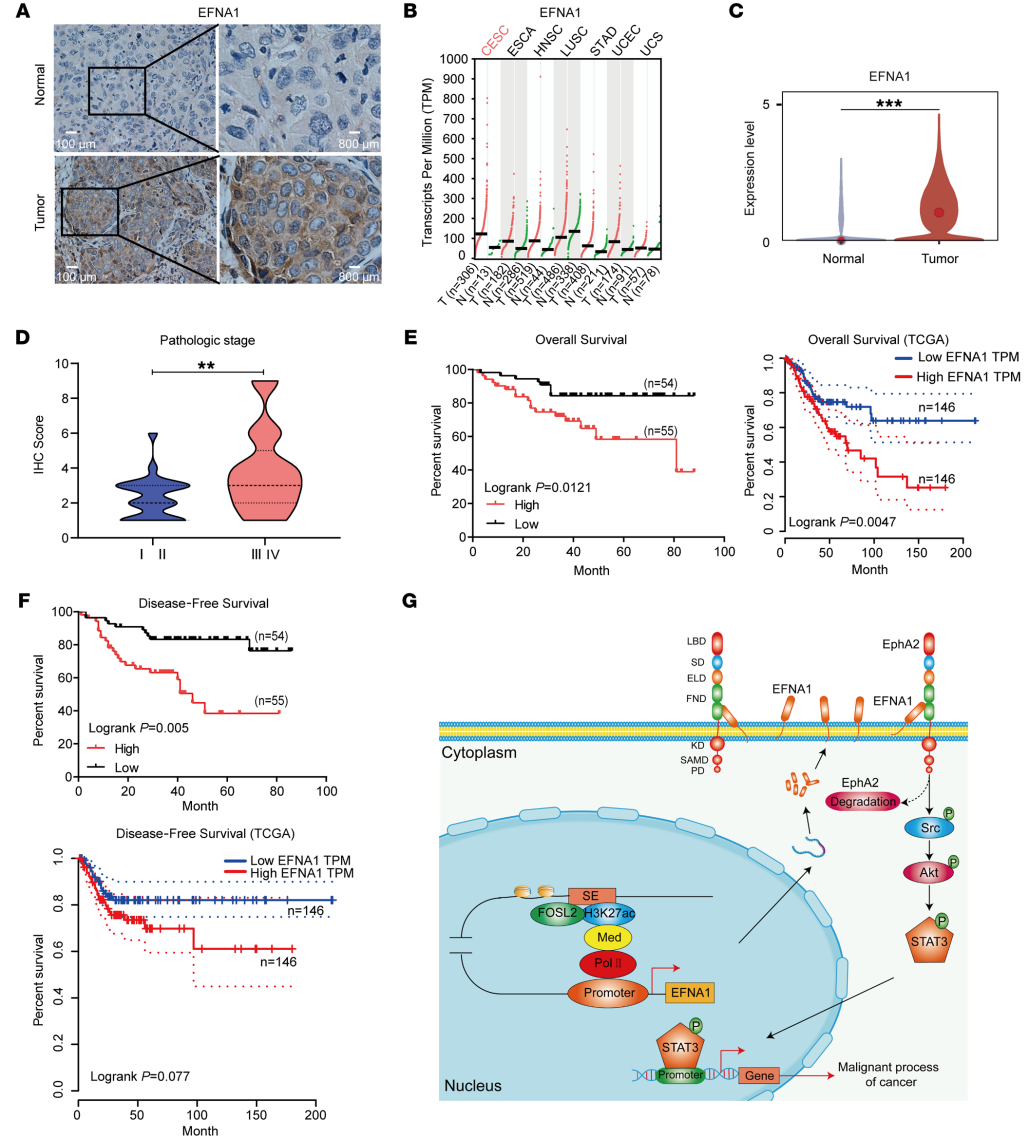
Elevated EFNA1 expression correlates with poor prognosis in CC patients. (**A**) Representative IHC images showing EFNA1 expression in cervical tumors and their paired normal tissues from Figure 1. Scale bars: 100 μm (left), 800 μm (right). (**B**) Transcriptome analysis of *EFNA1* expression in CC and other squamous cell carcinoma from the TCGA database. The *y* axis represents expression levels in transcripts per million, while the *x* axis lists various cancer types. CESC, cervical squamous cell carcinoma; ESCA, esophageal cancer; HNSC, head and neck cancer; LUSC, lung squamous cell carcinoma; STAD, stomach adenocarcinoma; UCEC, uterine corpus endometrial carcinoma; UCS, uterine carcinosarcoma. (**C**) Violin plot showing *EFNA1* mRNA levels in cervical tumor cells and normal cells using public single-cell sequencing data. (**D**) IHC scoring for EFNA1 in an independent cohort of CC patients (*n* = 109). Patients were categorized by T stages: I–II and III–IV. (**E** and **F**) Kaplan-Meier survival curves illustrating overall survival (**E**) and disease-free survival (**F**) rates for CC patients stratified by EFNA1 protein levels as determined by IHC and mRNA expression data from the TCGA database. Patients were divided into 2 groups based on the median expression level of EFNA1. (**G**) A schematic diagram of the proposed working model: *EFNA1*-SEs recruit transcription factors, particularly FOSL2, to enhance *EFNA1* transcription, which subsequently activates the Src/AKT/STAT3 signaling axis, driving tumorigenesis in CC. Statistical analysis was performed using 2-tailed *t* test in **C** and **D**, log-rank test in **E** and **F**. Significant *P* values: **P < 0.01, ***P < 0.001.

To evaluate the clinical significance of EFNA1 in CC, we conducted immunohistochemistry (IHC) staining assays for EFNA1 in the CC samples previously used for ChIP-Seq analysis (*n* = 9). We observed significantly higher EFNA1 expression in tumor regions compared with their paired non-cancerous normal tissues (Figure 8A). Transcriptome analysis of multiple cancers from the TCGA database also revealed that *EFNA1* transcription is tumor specific in CC compared with other squamous cell carcinomas (Figure 8B). Furthermore, single-cell transcriptome analysis indicated that *EFNA1* was strikingly upregulated in malignant cervical cells compared with normal cervical cells (Figure 8C and Supplemental Figure 11B). Moreover, IHC staining in an expanded CC patient cohort (*n* = 109) demonstrated a positive correlation between EFNA1 expression and tumor staging (Figure 8D and Supplemental Figure 17). Kaplan-Meier survival analysis further showed that patients with higher EFNA1 expression had worse overall and disease-free survival compared with those with lower EFNA1 expression (Figure 8E, left, and Figure 8F, top), consistent with the observations based on the TCGA dataset (Figure 8E, right, and Figure 8F, bottom). Collectively, these observations suggest that EFNA1 plays a pivotal role in CC progression and could serve as an unfavorable prognostic marker for CC patients.

## Discussion

For the first time to our knowledge, we have unveiled divergent epigenetic landscapes between cervical malignancies and their normal counterparts by conducting a comprehensive analysis of ChIP-Seq data for active enhancer (H3K27ac) and clinical biopsy samples. This approach offers an advantage over past studies that primarily relied on cancer cell lines, which often exhibit substantial epigenomic alterations due to extended culturing (35) and frequently lack matching normal derivatives as controls, complicating the identification of authentic tumor-specific epigenetic modifications (20). By integrating ChIP-Seq and RNA-Seq data, we identified numerous aberrant SEs and SE-associated genes, among which *EFNA1* is spotlighted as a novel tumor-specific SE target in CC. Our study provides multifaceted evidence demonstrating that *EFNA1* transcription is precisely regulated by specific SEs in the same locus (*EFNA1-SE*), underscoring its pivotal role in CC tumorigenesis. Additionally, our study corroborates the involvement of an SE-associated gene, *KLHDC7B*, in the development of CC, as reported previously (36), thereby highlighting the robustness of our approach.

Through ChIP-Seq analysis, binding motif matching, and experimental validations, we identified *FOSL2* as a critical TF that directly binds to the *EFNA1-SE* and regulates its transcription. This finding is consistent with the established notion that SEs enhance target gene expression through recruitment of abundant TFs (37). For instance, TP63 and SOX2 bind to the SEs and promoter of CCAT1, driving its expression and thereby activating the oncogenic EGFR pathway in squamous cell carcinoma (38). Similarly, RUNX3 binds to the TOX2 SE, driving its transcription and promoting oncogenesis in natural killer/T cell lymphoma (39). As a member of the AP-1 TF family, FOSL2 has been implicated in various cancers, emphasizing its role in tumorigenesis and disease progression (40, 41). Moreover, previous studies have demonstrated that SEs are crucial epigenetic structures that recruit FOSL2 through their core components, thereby directly facilitating target gene expression (42). Notably, our study reveals a strong interaction between FOSL2 and H3K27ac in CC cells, further underscoring the pivotal role of FOSL2 engagement in the *EFNA1-SE* to drive oncogenic *EFNA1* expression in CC.

SEs are predominantly characterized as oncogenic drivers, promoting genes that favor tumor development (43). Our study identifies *EFNA1* as an SE-driven proto-oncogene in CC, underlining its notable upregulation and correlation with poor patient survival. EFNA1, a GPI-anchored cell surface protein, plays a crucial role in tumorigenesis, diverging from its family members, such as EFNA2 and EFNA5, which are more commonly associated with cell differentiation (31). This divergence is evident in our study, in which *EFNA2* and *EFNA5* overexpression reduced cell proliferation, migration, and tumor growth, in contrast to EFNA1’s tumor-promoting effects. While some studies underscore *EFNA1*’s inhibitory effect on proliferation and invasion in malignancies (44–47), others emphasize its pro-metastatic tendencies (48–50), suggesting a dualistic function that may vary depending on cancer types and specific SE-driven activations.

Our study further elucidates a functional mechanism where EFNA1 *cis*-interacts with its receptor EphA2, leading to EphA2 degradation and subsequent activation of the Src/AKT/STAT3 pathway, thereby promoting tumor progression in CC. Eph-ephrin signaling networks are inherently complex, occurring both in *trans* between opposing cell membranes and in *cis* on the same membrane, with bidirectional signaling playing a pivotal role in cancer development and progression (34). We identified a notable interaction between EphA2 and EFNA1 that operates independently of the canonical LBD, forming a *cis* complex that results in the dephosphorylation and degradation of EphA2. This finding aligns with previous findings that ephrin ligand binding to Eph receptors typically triggers receptor internalization, often leading to degradation (51). Given that EphA2 negatively regulates AKT phosphorylation (47), our study demonstrates that EFNA1 induces EphA2 degradation, thereby activating the Src/AKT/STAT3 pathway. Activation of this pathway is well established in promoting tumorigenesis across various malignancies (52–54). In alignment with this, we observed that the application of Src, AKT, and STAT3 inhibitors substantially suppressed tumorigenic effects on CC cells. Notably, our findings revealed that these inhibitors effectively abrogate the enhanced tumorigenic effects conferred by EFNA1 overexpression. These findings underscore the potential therapeutic applications of targeting the EFNA1, EphA2, and Src/AKT/STAT3 signaling axis in CC patients, particularly in those with EFNA1 upregulation.

Notably, our study reveals that the *EFNA1-SE* signature is restricted to 7 of 24 cancer types, with a remarkably high prevalence (56%) in CC, underscoring the substantial heterogeneity of SE landscapes across different malignancies and individual patients. Given that SEs emerge from dynamic interactions among multidimensional factors such as TFs, epigenetic modifiers, chromatin architectures, and viral infection (37), this heterogeneity likely reflects distinct regulatory programs activated in different tumorigenic contexts. Specifically, the composition of TFs bound to SEs varies among cancer types, aligning with diverse molecular characteristics and unique tumor microenvironment (55). Since HPV infection is a known risk factor for CC and has been associated with SE activation (3, 23), it is plausible that HPV infection may contribute to the specific activation of *EFNA1-SE*, a hypothesis that warrants further investigation. Additionally, our transcriptomic analysis of tumors with *EFNA1-SE* revealed EFNA1 upregulation primarily in CC and colon adenocarcinoma, demonstrating a tumor-specific expression pattern. This specificity may be attributed to the selective recruitment of key TFs, such as FOSL2, to the SE regions in these tumor types, highlighting a potential mechanism for *EFNA1-SE*–driven oncogenesis in CC and beyond.

In summary, our study enriches the understanding of the intricacies and role of SEs in CC, by leveraging epigenomic profiling of tumor specimens alongside normal controls. We identify EFNA1 as a crucial SE-driven oncogene in CC, uncovering a sophisticated regulatory network involving SEs, the transcription factor FOSL2, the signaling receptor EphA2, and the transcriptional orchestrators Src/AKT/STAT3 pathway, key regulators within which govern the transcription of downstream target genes (Figure 8G). Furthermore, our findings demonstrate that modulating the activation of the Src/AKT/STAT3 pathway can effectively reverse the oncogenic effect of *EFNA1*. These insights provide promising clinical implications for CC, including patient stratification based on elevated EFNA1 expression for poor prognostic assessment and the development of targeted therapeutic strategies. Specifically, interventions targeting regulatory mechanisms modulated by the *EFNA1-SE* such as the SE complex (BRD4 and FOSL2), EFNA1, EphA2, and the downstream Src/AKT/STAT3 signaling axis hold promise for improving treatment outcomes in CC. However, we acknowledge certain limitations. First, while inhibition of the Src/AKT/STAT3 pathway partially mitigated the tumorigenic effects of *EFNA1*, other *EFNA1*-regulated pathways may also contribute to CC pathogenesis and await further investigation. Second, although deactivating the Src/AKT/STAT3 pathway suppresses tumorigenesis even in control CC cells, additional factors responsible for activating this pathway and the specific role of EFNA1 in this broader context require more in-depth exploration. Finally, we observed notable downregulated SEs accompanied by reduced expression of SE-associated genes in CC. Future work is needed to explore the contributions of these downregulated SEs to CC development.

## Methods

Further information can be found in Supplemental Methods.

### Sex as a biological variable.

Our study focused exclusively on female mice and patients, as it specifically investigates cervical cancer, a disease that occurs in women.

### Clinical sample collection and preparation.

For ChIP-Seq and RNA-Seq analyses, we collected 9 pairs of fresh cervical cancer (CC) samples along with their matched normal tissues from patients diagnosed with CC at the Sun Yat-sen University Cancer Center (SYSUCC), Guangzhou, China. The representative H&E histopathology results are provided in Supplemental Figure 1. Because most CC samples were identified at stage IB3 or beyond, according to the International Federation of Gynecology and Obstetrics (FIGO) classifications (56), adjacent normal tissues were not available for comparison. Therefore, we obtained the matched normal samples from the vaginal epithelial tissues adjacent to the tumors. Detailed clinical information on the patients is provided in Supplemental Table 1. All fresh samples were immediately frozen in liquid nitrogen before downstream processing. Total RNAs were extracted from these samples for subsequent RNA-Seq and reverse transcription–PCR.

For IHC analysis, we included a set of 109 paraffin-embedded CC biopsy samples from patients who had been histopathologically diagnosed by at least 2 pathologists in accordance with the WHO classification at SYSUCC between January 2013 and June 2020. Clinicopathologic classifications and staging were determined based on the 2008 American Joint Committee on Cancer pTNM staging system guidelines for CC.

### In situ Hi-C library construction.

Hi-C libraries were constructed using a total of 1 × 10^7^ cells. Cells were first cross-linked with 1% formaldehyde, followed by nucleus extraction using a hypotonic solution. The genomic DNA was digested with the restriction enzyme MboI (New England Biolabs [NEB]) at 3°C overnight, and the cohesive ends were filled in with biotinylated nucleotides. Blunt-end proximity ligation was then performed with T4 DNA ligase (NEB) at 16°C for 4 hours. After de-cross-linking with proteinase K (Thermo Fisher Scientific) at 65°C overnight, the ligated DNA was purified by QIAamp DNA Mini Kit (QIAGEN) and sheared to approximately 400 bp fragments. Next, biotin-labeled ligation junctions were captured using Dynabeads MyOne Streptavidin C1 (Invitrogen). The Hi-C library was prepared using the NEB Next Ultra II DNA Library Prep Kit according to the manufacturer’s instructions and sequenced with paired-end 150 reads on the MGI DNBSEQ-T7 platform.

### ChIP-Seq assay and data generation.

ChIP assay was performed using the truChIP Chromatin Shearing Tissue Kit according to the manufacturer’s instructions (Covaris). The procedure is summarized as follows: For chromatin cross-linking, cells were treated with 1% formaldehyde in phosphate-buffered saline (PBS) for 10 minutes at room temperature. The cells were washed first with 5 mg/mL bovine serum albumin (BSA) in PBS and then with cold PBS, and were then lysed using a buffer containing 50 mM Tris-HCl (pH 8.1), 10 mM EDTA, 1% SDS, and 1× protease inhibitor cocktail. For chromatin shearing, chromatin was sonicated using a Covaris M220 to yield fragments ranging from 100 to 1,000 bp. The fragment size was determined by the Bioanalyzer DNA High Sensitivity kit (Agilent). The sheared chromatin was diluted with IP buffer (20 mM Tris-HCl [pH 8.1], 150 mM NaCl, 2 mM EDTA, 1% Triton X-100) and incubated overnight at 4°C with protein G magnetic beads precoated with anti-H3K27ac antibodies. The following day, the immunoprecipitate was washed 6 times using a wash buffer (50 mM HEPES [pH 7.6], 0.5 M LiCl, 1 mM EDTA, 0.7% Na deoxycholate, and 1% IGEPAL CA-630) (Merck KGaA), followed by 2 washes with TE buffer (10 mM Tris-Hcl [pH 8.0] and 1 mM EDTA). For DNA extraction, both the immunoprecipitated and the input DNA was treated with RNase A and proteinase K. The DNA was subsequently eluted using a mixture of 1% SDS and 0.1 M NaHCO_3_, followed by incubation at 65°C for 7 hours. DNA purification was achieved using the DNA Clean & Concentrator kit (Zymo Research). A library was constructed using up to 10  ng of the extracted DNA with the NEB Next Ultra II DNA Library Prep Kit (E7645). DNA sequencing was performed on the NovaSeq 6000 system in accordance with the manufacturer’s instructions (Illumina). On average, 47.2 million reads per sample were obtained, with 150 bp for each paired-end read.

### ChIP-Seq analysis, super-enhancer identification, and differential super-enhancer determination.

ChIP-Seq reads were first processed to remove adapter sequences using Trim Galore and subsequently aligned to the human reference genome (hg38) with Bowtie 2 (57). PCR duplicates were eliminated with Picard (v2.27.4) (https://broadinstitute.github.io/picard/). Significant peaks corresponding to certain genomic regions were identified using MACS3 (v3.0.0a7) (58) with default parameters, yielding a narrow peak bed file. After removal of the peaks in the blacklist database (59) (https://github.com/Boyle-Lab/Blacklist/tree/master), which contains repeat sequences, the remaining peaks were subjected to the ROSE algorithm to identify super-enhancers for each sample (11).

In the ROSE algorithm, active typical enhancers were defined as significant H3K27ac peaks situated at least 2 kb away from the nearest transcription start sites. SE components within a 12.5 kb range were stitched together and ranked according to the H3K27ac signal in each tumor and normal sample. Subsequently, the resulting SEs from the 9 tumor samples and their corresponding normal tissues were merged using bedtools (v2.29.1) (60). The intensity of each SE was then computed individually for each sample using featureCounts (v2.0.1) (61), with SE signals normalized as reads per kilobase per million mapped reads (RPKM). The RPKM matrix was used to conduct principal component analysis using the “prcomp” function implemented in the R package stats (version 4.2.0). To identify differential SEs between CC and its matched normal tissues, Wilcoxon’s rank sum test was used, and the resulting *P* values were adjusted using FDR. SEs with fold changes exceeding 1.5 or below 0.67 in CC compared with matched normal tissues were considered as significantly differential. Additionally, potential target genes linked to each SE were defined based on the closest gene, as annotated by the ROSE algorithm. Bigwig files were generated using deepTools2 (62), and data visualization was conducted using Integrative Genomics Viewer (v2.12.3) (63).

### Cell culture and reagents.

Human embryonic kidney (HEK) 293T cells were obtained from the Cell Bank of Type Culture Collection of Chinese Academy of Sciences, Shanghai Institute of Cell Biology, Chinese Academy of Sciences. Human SiHa cervical cancer cells were purchased from Shanghai Biowing Applied Biotechnology Co. Ltd., while HCC-94 cells were obtained from Guangzhou Cellcook Biotech Co. Ltd. HEK293T and SiHa cells were cultivated in DMEM (Gibco) supplemented with 10% fetal bovine serum (FBS; Gibco) and 1% penicillin-streptomycin (Gibco). HCC-94 cells were cultured in MEM (Gibco) with 10% FBS and 1% penicillin-streptomycin (Gibco). All cells were incubated at 37°C with 5% CO_2_ in a humidified incubator. Regular screenings confirmed that all cell lines were free from contamination, as verified using a mycoplasma detection kit (Vazyme, Nanjing, China). Cell line authentications were confirmed through short tandem repeat analysis. The compounds JQ1 (Selleck, S7110) and saracatinib (Selleck, AZD0530) were obtained from commercial sources.

### siRNA, plasmids, and lentivirus.

Small interfering RNA (siRNA) oligonucleotides targeting *EFNA1* and *FOSL2* were commercially synthesized (GenePharma) and transfected into cells using Lipofectamine RNAiMAX (Thermo Fisher Scientific) following the manufacturer’s protocols. Short hairpin RNAs (shRNAs) targeting human *EFNA1* were synthesized (Rui Biotech) and constructed into pLKO.1-puromycin lentiviral vectors. The full-length cDNAs of *EFNA1*, *EFNA2*, and *EFNA5* were subcloned into pCDH-puromycin vectors. For lentivirus production, HEK293T cells were cotransfected with the respective vectors along with lentivirus packaging plasmids (psPAX2 and pMD2.G) using Lipofectamine 2000 (Thermo Fisher Scientific). Lentiviruses were harvested and used to infect the targeted CC cells, followed by puromycin selection. Primer sequences are listed in Supplemental Table 5.

### Generation of truncated mutations of EphA2.

FLAG-tagged wild-type EphA2 (EphA2^WT^) was cloned into the pCDH-CMV-MCS-EF1-Puro vector. A series of truncated EphA2 mutants were generated based on the EphA2^WT^: EphA2^ΔLBD^, lacking the ligand-binding domain (LBD); EphA2^ΔLSEF^, lacking the LBD, the cysteine-rich domain, and the first FNIII domain but retaining the membrane-proximal FNIII domain; EphA2^ΔFNIII^, lacking the entire FNIII domain; EphA2^ΔEXT^, lacking the entire extracellular portion; and EphA2^ΔLBD-KD^, which retains the same extracellular region as EphA2^ΔLBD^ but lacks the entire intracellular domain. All truncated EphA2 mutants were constructed using the ClonExpress Ultra One Step Cloning Kit (Vazyme, C115). The primer sequences used for these constructs are provided in Supplemental Table 5.

### Immunohistochemical staining.

IHC was carried out as described previously (64). In brief, paraffin-embedded tissue sections underwent deparaffinization in xylene and rehydration. Antigen retrieval was then done using sodium citrate, followed by quenching of peroxidase activity with 3% hydrogen peroxide. After blocking with 5% goat serum in TBS-T, sections were incubated overnight at 4°C with the designated primary antibodies. The next day, they were incubated with secondary antibodies at a 1:500 dilution for 1 hour at room temperature. Chromogenic immunolocalization was conducted using 0.05% 3,30-diaminobenzidine (Dako), and sections were subsequently counterstained with hematoxylin. Immunostaining results were analyzed and scored independently by 2 pathologists at SYSUCC. Staining intensity was scored on a scale of 0 (negative), 1–2 (intermediate), and 3 (strong), with final IHC scores calculated based on staining intensity and the percentage of stained cells.

### RNA isolation and reverse transcription–qPCR.

Total RNA was extracted from cell lines and tissues using Trizol (Thermo Fisher Scientific) and was then converted to cDNA using oligo-dT primers and M-MLV Reverse Transcriptase Kit (Promega), according to the manufacturers’ protocols. The resulting cDNA was quantified via qPCR using the SYBR Green PCR kit (Takara) on a CFX96 Touch Sequence Detection System (Bio-Rad). All samples were normalized to internal genes, and relative fold changes were calculated using the relative quantification method (2^–ΔΔCt^). Every experiment was conducted in triplicate. The primer sequences are provided in Supplemental Table 5.

### Western blotting.

Cells or tissues were lysed with RIPA lysis buffer (Beyotime) supplemented with a protease and phosphatase inhibitor cocktail (Beyotime) for 30 minutes. Protein concentrations were determined using the bicinchoninic acid (BCA) assay using a BCA kit (Beyotime) according to manufacturer’s instructions. Equal amounts of protein from cell lysates were then subjected to electrophoresis on an SDS-PAGE gel and transferred onto PVDF membrane (Millipore). The membranes were subsequently blocked in 5% BSA (Sangon Biotech) in TBS-T for 60 minutes at room temperature, followed by overnight incubation with the specified primary antibodies at 4°C. The following day, after washing 3 times with TBS-T, the membranes were incubated with HRP-conjugated secondary antibodies. Immunoreactive protein bands were visualized using the FDbio-Dura ECL kit (FDbio Science Biotech) and Bio-Rad ChemiDoc Touch. GAPDH and β-actin were used as internal loading controls. Details for all antibodies are provided in Supplemental Table 6.

### Cell proliferation and colony formation assays.

Cell proliferation was assessed using the Cell Counting Kit-8 (Dojindo Laboratories). Cells were seeded at a density of 1 × 10^3^ per well in a 96-well plate, with 5 replicates for each condition. The cells were incubated for 2 hours at 37°C, and the absorbance at 450 nm was measured daily for 5 consecutive days, per the manufacturer’s instructions. For colony formation assay, 3 × 10^3^ treated cells were plated in 6-well plates, with each condition replicated 3 times. After a 10-day incubation, these plates were gently rinsed twice with PBS, fixed with formalin for 15 minutes, and stained with a 0.5% crystal violet solution for 10 minutes. Subsequently, colonies were counted under a light microscope by examination of 6 randomly chosen representative fields for each sample.

### Cell cycle and apoptosis assays.

For cell cycle assay, cells were harvested and fixed in chilled 70% ethanol, then stored at –20°C overnight or longer. After fixation, the cells were washed twice with PBS and resuspended. They were then incubated with 1 mL of staining solution (1 mg/mL propidium iodide; Thermo Fisher Scientific) for 30 minutes at room temperature. Cell cycle distribution was assessed using an SP6800 Spectral Analyzer (Sony) and analyzed using FlowJo v10 software. Cell apoptosis was evaluated through flow cytometry using the Dead Cell Apoptosis Kit with Annexin V Alexa Fluor 488 & Propidium Iodide (Thermo Fisher Scientific) per the manufacturer’s instructions. CC cells (2.5 × 10^5^) were seeded in each well of a 6-well plate in triplicate. Once the cells reached a density of 60%–80%, they were harvested, washed with cold PBS, and resuspended in 1× binding buffer. The cells were then stained with annexin V and propidium iodide for 15 minutes at room temperature. Apoptosis analysis was carried out using the same flow cytometry setup and software as were used for the cell cycle assay.

### Cell migration and invasion assays.

Migration or invasion assays were carried out using 24-well Transwell plates with filter inserts of 8 μm pore size (Corning), with (migration) or without (invasion) Matrigel precoating. Approximately 5 × 10^4^ cells suspended in serum-free medium were seeded into the upper chamber, while the bottom chamber was filled with medium containing 10% FBS. After 24 hours of incubation at 37°C, cells that had migrated to the underside of the membrane were fixed with 4% paraformaldehyde for 10 minutes and stained with 0.5% crystal violet. Migrated cells were then counted in 5 random fields under a light microscope.

### Double-CRISPR genome editing using CRISPR/Cas9.

To delete the SEs of *EFNA1*, we designed sgRNA sequences targeting regions flanking the SEs, within a 500 bp range, using the online tool CHOPCHOP (https://chopchop.cbu.uib.no/). Subsequently, these sgRNA sequences were individually cloned into the LentiCRISPR VII plasmid (Addgene) for lentivirus production. Lentiviruses were then used to infect CC cells. After a 3-day puromycin selection, successfully transfected cells were seeded at subcloning density to obtain knockout clones. Knockout efficiency was confirmed by immunoblot and sequencing techniques. To further verify the deletion efficiency of the SEs, PCR primers were designed outside the CRISPR sgRNAs target sites, flanking the SE region. Given the effective CRISPR/Cas9–mediated DNA cutting and subsequent non-homologous end joining repair, we anticipated a PCR product of approximately 600 bp (Supplemental Table 5).

### Dual-luciferase reporter assays.

DNA fragments located 1,200 bp or 2,500 bp upstream of the *EFNA1* transcription start site, as well as those with mutant deletions at the FOSL2 binding sites, were subcloned into a luciferase reporter pGL3-basic plasmid. Subsequently, the SE constituents of *EFNA1*, labeled as E1 and E2, were amplified and constructed into the above *EFNA1* promoter luciferase reporter plasmids, resulting in constructs named pGL3-E1/E2-P. Then, approximately 1.5 × 10^5^ HEK293T cells were seeded into each well of a 24-well plate and cotransfected with the specified constructs along with *FOSL2* siRNAs, control siRNA, *FOSL2* overexpression plasmids, or control vectors. Forty-eight hours after transfection, transcription activity was assessed using the Dual-Luciferase Reporter Assay kit (Promega) according to the manufacturer’s instructions. Promoter activity was calculated as the ratio of firefly luciferase activity to Renilla luciferase activity.

### Human phosphorylated kinase array.

To explore potential downstream pathways, we used the Human Phospho-Kinase Array Kit (R&D Systems, ARY003C) according to the manufacturer’s protocols. In brief, CC cells transfected with *EFNA1* siRNA or control siRNA were lysed. Each of the 8 nitrocellulose membranes, containing 39 different capture antibodies arrayed in duplicate, was blocked with 2 mL Array Buffer 1 (R&D Systems) for 1 hour. Subsequently, the lysate samples were added to the wells and incubated overnight. After a series of washes to remove non-specifically bound proteins, a biotinylated detection antibody cocktail was applied. After additional washes to remove any unbound detection antibodies, the membranes were treated with a diluted streptavidin-HRP solution. Phosphorylated proteins were detected using the streptavidin-HRP conjugate in combination with chemiluminescent detection reagents from the FDbio-Pico ECL kit (FD Bioscience Biotech). The signals were captured using a Bio-Rad chemiluminescence imaging system, and signal densities were quantified and analyzed using ImageJ software (NIH).

### In vivo xenograft mouse model.

Six-week-old female BALB/c nude mice, obtained from Beijing Vital River Laboratory Animal Technology, were housed under specific pathogen–free conditions. To verify the specific oncogenic role of EFNA1 in CC, SiHa cells (1 × 10^6^) infected with EFNA1-, EFNA2-, or EFNA5-overexpressing or EFNA1 shRNAs (sh-EFNA1-1 and sh-EFNA1-2), or control lentiviruses were respectively mixed with Matrigel (0.20 vol/vol; Corning Inc.) and subcutaneously injected into the dorsal flank of each mouse (*n* = 8 mice per group). To assess the effect of saracatinib, MK2206, and Stattic on CC, mice with xenografted tumors derived from subcutaneous injection of either EFNA1-overexpressing or control SiHa cells were randomly assigned to 1 of 4 treatment groups for each inhibitor: (a) vector plus vehicle, (b) EFNA1 plus vehicle, (c) vector plus inhibitors, and (d) EFNA1 plus inhibitors. Treatment was initiated when xenografted tumor reached an average volume of 0.1 cm^3^. The mice received daily oral gavage for 21 days, with treatments including vehicle alone, saracatinib (50 mg/kg), MK2206 (120 mg/kg), and Stattic (10 mg/kg; *n* = 6 mice per group). Tumor size was measured every 3 days using a caliper. The mice were humanely sacrificed via cervical dislocation before the tumors reached a volume of 1,500 mm^3^. Tumors were excised, weighed, and photographed for further analysis. Tumor volume in mm^3^ (*V*) was determined using the formula *V* = *L* × *W* × *W*/2, where *L* is the length and *W* is the width of the tumor.

### Statistics.

Statistical analyses were conducted using SPSS 20.0 (SPSS) and Prism 8.0 (GraphPad). Each experiment was independently repeated at least 3 times. Measured data were presented as the mean ± SD. Quantitative data were analyzed using 1-way analysis of variance (ANOVA) or 2-tailed Student’s *t* test, while qualitative data were assessed using the nonparametric χ^2^ test. Pearson’s correlation analysis was applied to evaluate the correlation between EFNA1 expression and other genes. To identify independent prognostic factors, both univariate and multivariate analyses were carried out using Cox’s proportional-hazards regression model. Survival analysis, including overall and disease-free survival, was assessed using the Kaplan-Meier method, with differences determined by the log-rank test. *P* values indicating statistical significance are presented in the respective figures and defined as follows: *P < 0.05, **P < 0.01, ***P < 0.001, and ****P < 0.0001.

### Study approval.

All animal experiments were performed in compliance with protocols approved by the Institutional Animal Care and Use Committee of Sun Yat-sen University Cancer Center (Guangzhou, China) under approval SYSU-IACUC-2024-002650. Procedures involving human samples were conducted with approval from the Ethics Committee of Sun Yat-sen University Cancer Center (approval SL-B2022-069), and informed consent was obtained from all patients.

### Data and materials availability.

Essential data were deposited in the Research Data Deposit public platform (RDD, RDDB2025692461, http://www.researchdata.org.cn). The raw sequence data in this study were deposited in the Genome Sequence Archive (65) in the National Genomics Data Center (66), China National Center for Bioinformation/Beijing Institute of Genomics, Chinese Academy of Sciences, and are publicly accessible at https://ngdc.cncb.ac.cn/gsa-human (GSA-Human: HRA005901). Data included in this study are provided in the Supporting Data Values file and are also available upon request.

## Author contributions

JXB and CLL designed the study. JXB, SQL, and SH procured financial support. SQL, CYL, PPW, ZHX, SHY, MZ, MSH, and DLZ performed sample recruitment and preparation and data collection. SH, TX, YQL, YL, CL, and SLS analyzed and interpreted data. SQL, XXC, WP, and YQZ performed functional experiments. SQL, CLL, XXC, SH, and JXB wrote the original draft of the paper. All authors approved the final report.

## Supplementary Material

Supplemental data

Unedited blot and gel images

Supplemental tables 1-6

Supporting data values

## Version 1 02/18/2025

In-Press Preview

## Version 2 04/15/2025

Electronic publication

## Version 3 08/03/2026

Revised Supplemental Figure 4 and supporting data values per corrigendum
