# Corrigendum to Super-enhancer–driven EFNA1 fuels tumor progression in cervical cancer via the FOSL2-Src/AKT/STAT3 axis

**DOI:** 10.1172/JCI209744

**Published:** 2026-08-03

**Authors:** Shu-Qiang Liu, Xi-Xi Cheng, Shuai He, Tao Xia, Yi-Qi Li, Wan Peng, Ya-Qing Zhou, Zi-Hao Xu, Mi-Si He, Yang Liu, Pan-Pan Wei, Song-Hua Yuan, Chang Liu, Shu-Lan Sun, Dong-Ling Zou, Min Zheng, Chun-Yan Lan, Chun-Ling Luo, Jin-Xin Bei

Original citation: *J Clin Invest.* 2025;135(8):e177599. https://doi.org/10.1172/JCI177599

Citation for this corrigendum: *J Clin Invest.* 2026;136(15):e209744. https://doi.org/10.1172/JCI209744

After publication of this article, the authors became aware of the following errors: In Supplemental Figure 4F, the HR and *P* values for RHOF were inadvertently transposed; the correct values are HR = 1.2 and *P* = 0.59. In Supplemental Figure 16C, for the HCC-94 cells (right panel), the values with Stattic treatment were inadvertently duplicated from the values with Saracatinib treatment presented in Figure 7C. The Supplemental material PDF and Supporting Data Values, have been updated.

The authors regret these errors.

## Version 1 08/03/2026

Electronic publication

