# Supplementary material for: Super-enhancer–driven EFNA1 fuels tumor progression in cervical cancer via the FOSL2-Src/AKT/STAT3 axis
Source: J Clin Invest. 2025 Feb 18;135(8):e177599. doi: 10.1172/JCI177599 (PMC11996870; doi:10.1172/JCI177599)
Supplement: Supplemental data [file jci-135-177599-s009.pdf]

Supplemental Notes

Supplemental Note 1. Analysis of public datasets

We utilized transcriptome data from The Cancer Genome Atlas (TCGA) and Genotype-Tissue Expression (GTEx) databases to analyze the expression patterns of *EFNA1* gene between CC and normal cervical epithelial tissues, and to determine the correlation between SE-associated genes with the prognosis of CC patients. This analysis was facilitated by Gene Expression Profiling Interactive Analysis 2 (GEPIA2, http://gepia2.cancer-pku.cn/) (1).

For Single-cell RNA-seq (scRNA-seq) data, we utilized Li's dataset to obtain data specific to CC (2). Tumor and normal cervical epithelial cells were identified using annotations provided in the original publication. Normalized expression of *EFNA1* was calculated using Seurat package (v3.2.3) (3) and the significant differences were estimated using the Willcox Test.

Supplemental Note 2. RNA-seq analysis

Total RNA was extracted from both the cell lines and CC tissues using trizol reagent (Thermo, Waltham, USA). After evaluation of RNA quality using a Qseq 100 Bioanalyzer (BIOptic, Taiwan, China), Ribosomal RNA depletion was then performed using the Ribo-Zero Magnetic kit (Illumina, California, USA). Subsequently, RNA sequencing libraries were prepared using the TruSeq RNA Library Prep Kit (Illumina, California, USA) and sequenced on the NovaSeq 6000 system in accordance with the manufacturer's instructions (Illumina, San Diego, USA), achieving an average of 27.1 million reads per sample with 150 bp paired-end reads.

Adapter sequences were trimmed from the reads using the Trim Galore (version 0.6.1, http://www.bioinformatics.babraham.ac.uk/projects/trim_galore/). High-quality pair-end reads were first aligned to ribosomal RNAs using Bowtie2 (4). After removing the ribosomal RNA-aligned reads, the remaining reads were then mapped to the human reference sequence (Hg38) using STAR with default settings (5). Raw counts and normalized expression values, represented as transcripts per million

(TPM), were calculated for each gene using RSEM (6). Differentially expressed genes (DEGs) were identified using the DESeq2 package (version 1.36.0) (7), with a false discovery rate (FDR) threshold set to 0.05.

Supplemental Note 3. Hi-C data analysis

The raw FASTQ sequencing data were first processed to trim adapter sequences using TrimGalore (version 0.6.1; https://github.com/FelixKrueger/TrimGalore). The clean reads were then aligned to the human genome (hg38) using BWA (version 0.7.18) (8). Subsequent analyses, including the creation of contact matrix, identification of TADs, and data visualization, were conducted using functions implemented in HiCExplorer (9) (version 3.7.3) in python. Default settings were applied for all parameters unless specified otherwise. Initially, the "hicFindRestSite" function was employed to identify restriction sites for the Phase Genomics cutting enzyme (GATC). After mapping reads, the Hi-C contact matrix was built using "hicBuildMatrix" the bin dize ("--binSize") set to 10 kb. This matrix was then corrected using "hicCorrectMatrix correct" function, applying the iterative matrix correction (ICE) method. Subsequently, the "hicFindTADs" tool was used on the ICE-normalized matrices to calculate a genome-wide TAD separation score, applying a delta value and correcting for multiple testing with an FDR threshold of 0.01. A false discovery rate (FDR) less than 0.05 was considered significant. Visualization of the Hi-C matrix was achieved using the "hicPlotTADs" function.

Supplemental Note 4. Motif analysis

Motif analysis was conducted using HOMER(10) with default settings. Predicted TF binding sites were ranked according to their respective *P* values.

Supplemental Note 5. Gene Set Enrichment Analysis (GSEA)

Differentially expressed genes (DEGs) were identified using DESeq2, applying an absolute log-fold change threshold greater than one. Enrichment analysis of SE-associated genes, particularly those active in cervical tumors or their matched

non-tumoral tissues, was subsequently conducted using the GSEA function implemented in the clusterProfiler package (version 4.4.4) (11).

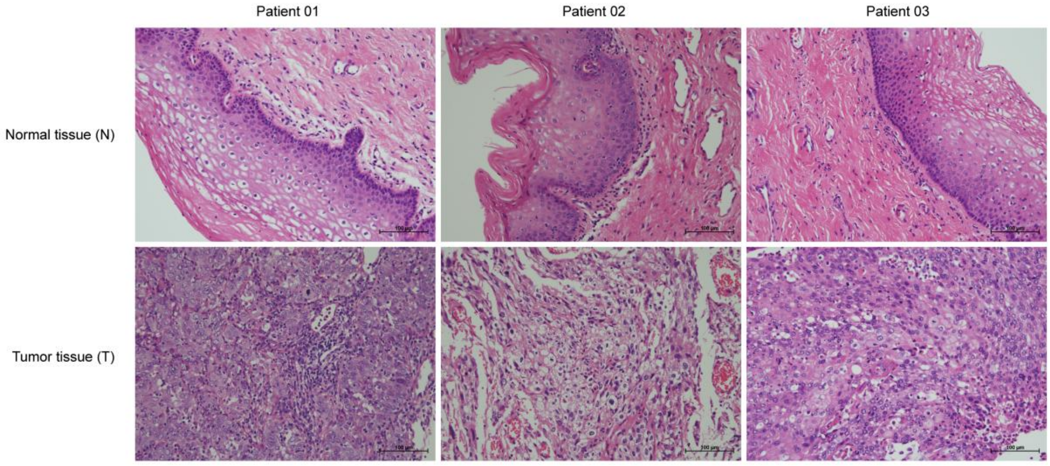

Supplementary Fig 1. Representative images for the H&E staining. Staining of normal and tumor tissues from cervical cancer patients showing normal tissues (N) and matched tumor tissues (T) from three cervical cancer patients (Patient 01, Patient 02, and Patient 03). Scale bar: 100 μm.

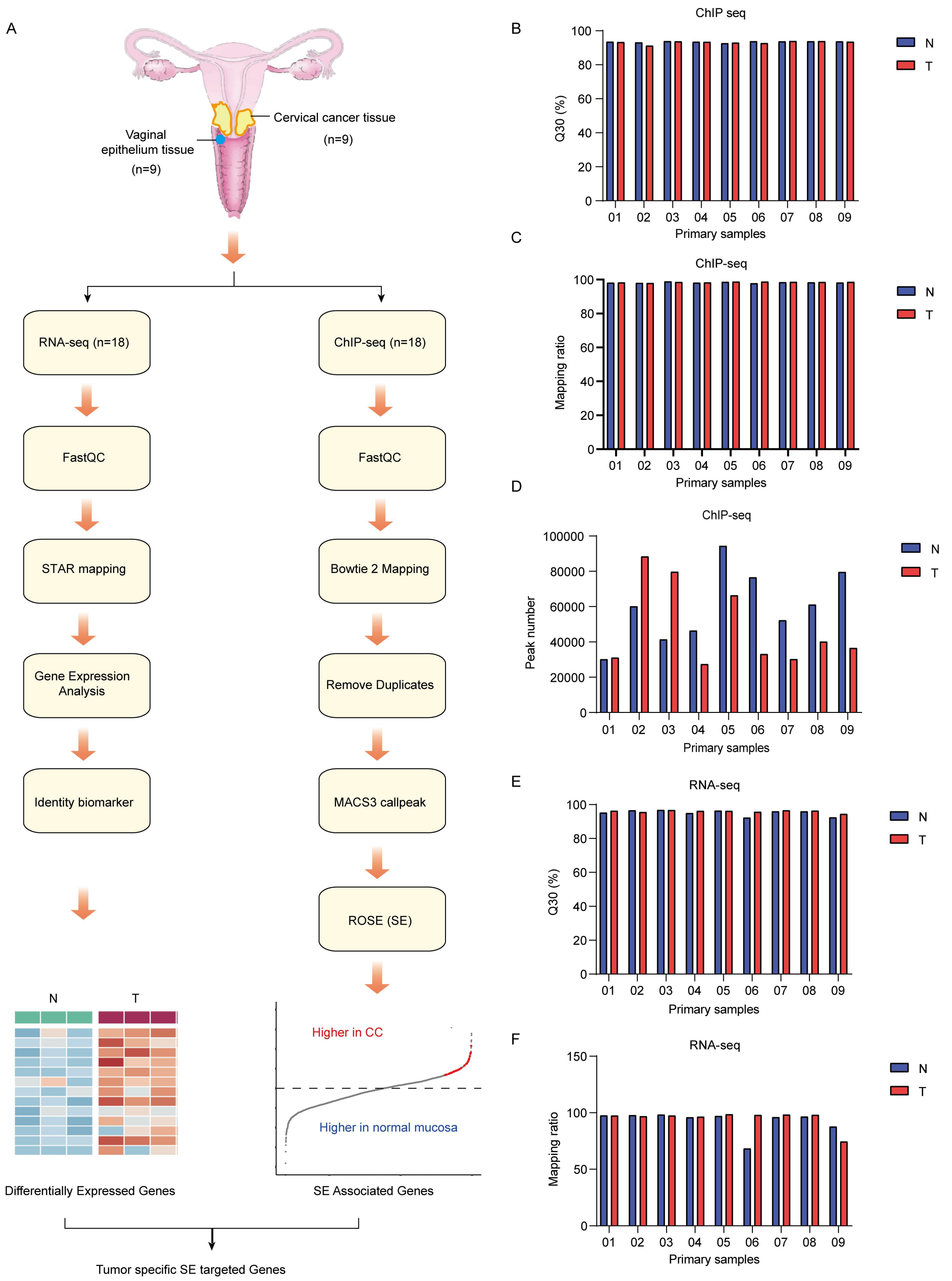

Supplementary Fig 2. Analytic pipeline and data quality assessment. (A) Overview of the Bioinformatics analysis procedures: H3K27ac ChIP-seq and RNA-seq datasets were generated from 9 CC patient pairs. After standard quality control and preprocessing, RNA-seq data were subjected to gene expression profiling, differential gene expression analysis, and

biomarker identification. Differential peak analysis was conducted on the ChIP-seq datasets. (**B-D)** Qualification measurements for ChIP-seq data. These include: (B) the proportion of ChIP-seq reads with base-call accuracy above Q30, ensuring 99.9% accuracy; (C) the proportion of successfully mapped reads; and (D) the number of H3K27ac peaks identified in 9 CC patient pairs. **(E-F)** Qualification metrics of RNA-seq data, including the proportion of RNA-seq reads with base-call accuracy above Q30 (E) and the proportion of successfully mapped reads (F).

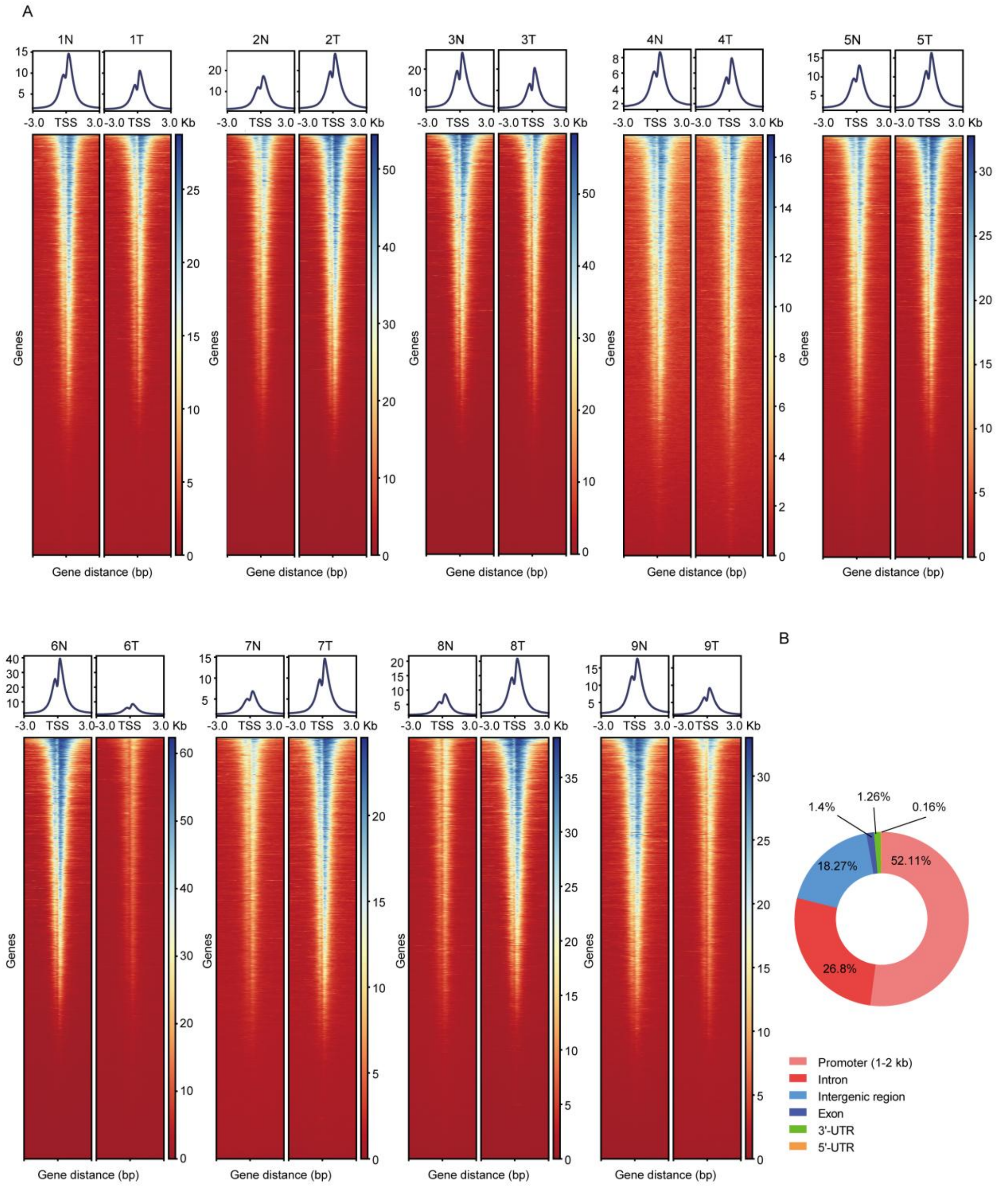

Supplementary Fig 3. H3K27ac enrichment patterns in CC tumors (T) and adjacent normal tissues (N). (A) Heatmap showing ChIP-seq signals for H3K27ac across regions ±3, 000 bp from the transcription start site (TSS) of each gene in both tumor and adjacent normal tissues. Patient IDs are indicated on top, and signaling intensity above represents the levels of H3K27ac enrichment. **(B)** Genomic distribution of CC-specific SEs. UTR, untranslated region.

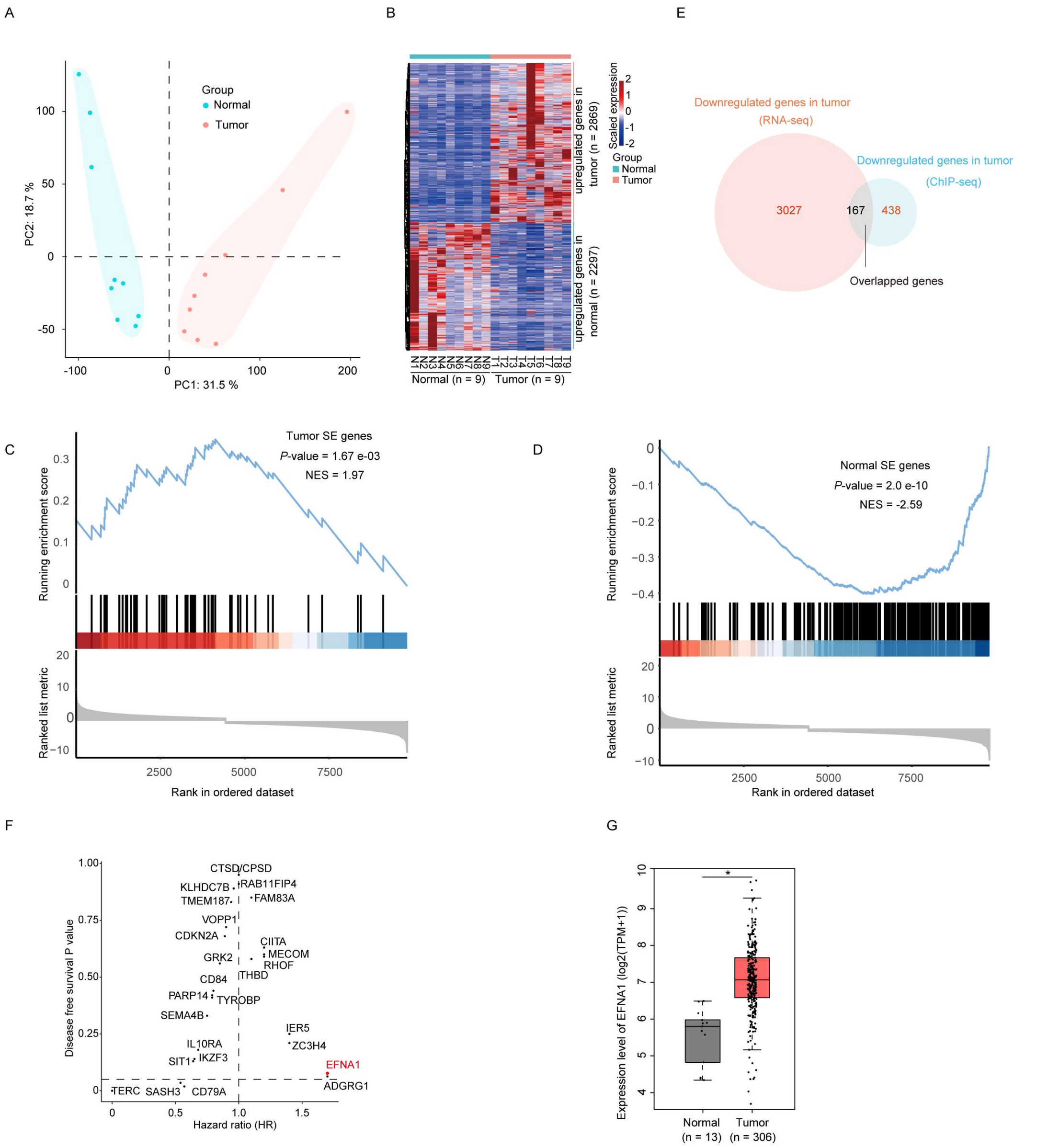

Supplementary Fig 4. Integrated analysis of ChIP-Seq and RNA-seq data from CC tumor and adjacent normal tissues. (A) PCA plot showing the top two principal components (PC1 and PC2) based on RNA-seq gene expression data from 9 CC samples and their matched adjacent normal tissues. Tumor and adjacent normal samples are represented by circles in different colors as indicated. **(B)** Heatmap illustrating the expression of 5166 differentially expressed genes between CC tumors and paired adjacent normal tissues. Gene expression level is TPM-normalized and row scaled. The color spectrum from red to blue indicates expression levels from high to low. **(C-D)** GSEA for genes proximal to elevated SEs in CC (**C**) or in adjacent normal tissues (**D**). *P* values are adjusted using Bonferroni correction. **(E)** Venn diagram illustrating the overlap of genes

downregulated in CC tumors that are targeted by repressive SEs in CC. **(F)** Scatter plot showing the associations of disease-free survival and 26 SE-targeted genes identified in **Fig. 1E**. Patients were divided into two groups based on the median expression level of each gene. (**G)** Box plot comparing *EFNA1* expression levels in CC versus normal cervical epithelial tissues utilizing data from both the TCGA and GTEx databases. Statistical analysis was performed using the Log-rank test in **F**, t-test in **G**. * P <0.05, ** P < 0.01, *** P < 0.001.

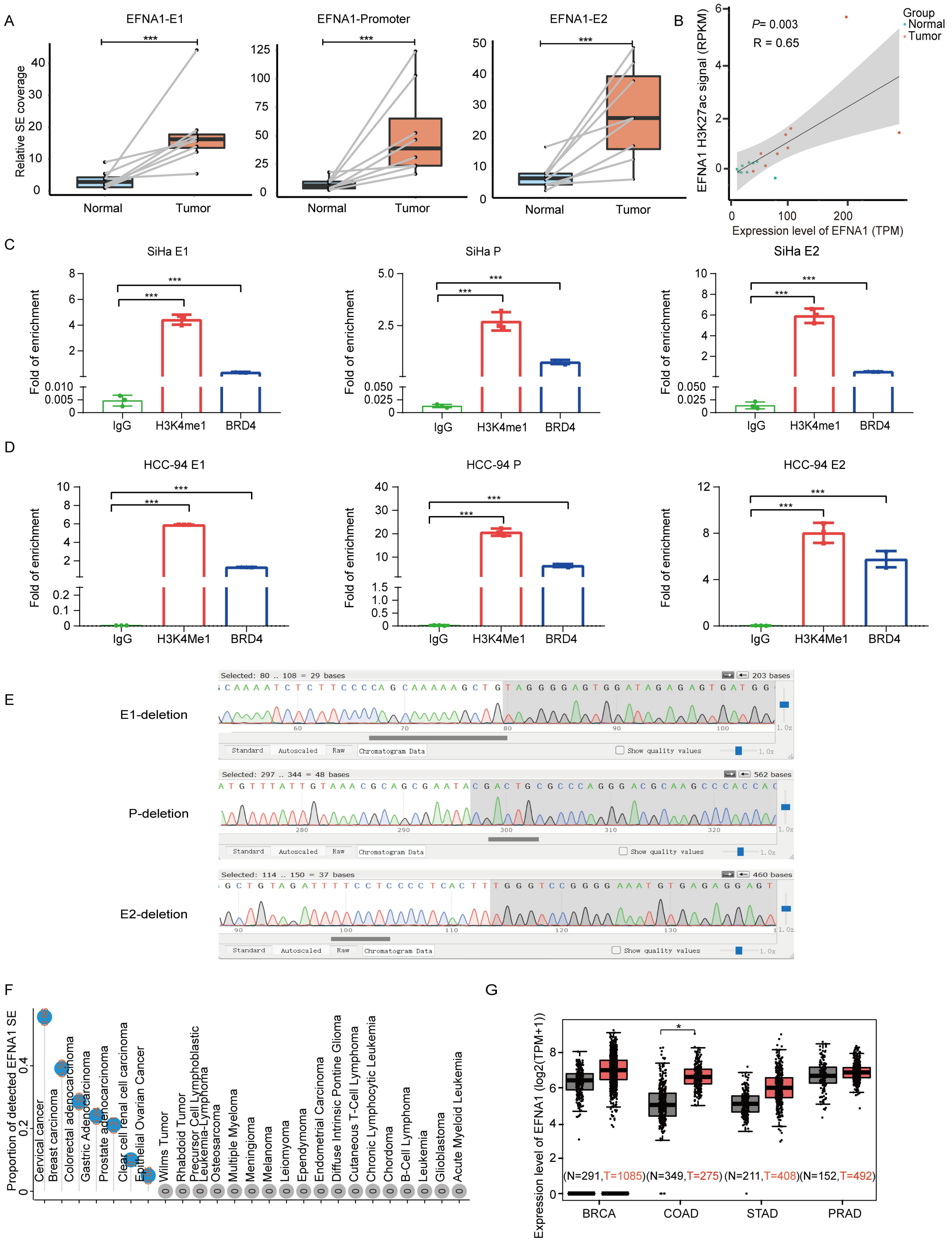

Supplementary Fig 5. Epigenomic profiling identifies putative SEs in cervical cancer. (A) ChIP-qPCR analysis showing H3K27ac enrichment within two SE regions (E1-E2) and promoter of *EFNA1* in cervical tumor and adjacent normal tissues. **(B)** Scatter plot showing Pearson's correlation between *EFNA1* expression (x axis) and H3K27ac signal intensity (y axis) at the *EFNA1*-SE locus in CC tumors and matched adjacent tissues. **(C-D)** ChIP-qPCR analysis evaluating H3K4me1 and BRD4 enrichments at the two SE regions (E1-E2) and the promoter of

EFNA1 in SiHa (C) and HCC-94 (D) cell lines. **(E)** Sanger sequencing results confirming the CRISPR/Cas9-mediated deletion of EFNA1-SE regions in SiHa cells. **(F)** Lollipop chart showing the proportion of *EFNA1*-SEs detected across 25 distinct primary tumor types. Each dot represents a detected ratio for *EFNA1*. Dots in blue and grey indicate detected and non-detected *EFNA1*-SEs, respectively. Except for CC data, the remaining 24 tumor types are obtained from the CenhANCER database (12). Only tumors with more than five samples were included. **(G)** Box plot comparing *EFNA1* expression levels between tumor (T) and normal tissues (N) in various cancer types, using data from the TCGA and GTEx. BRCA: Breast invasive carcinoma, COAD: Colon adenocarcinoma, STAD: Stomach adenocarcinoma, PRAD: Prostate adenocarcinoma. Between-group comparisons: t-test in **A** and **G;** one-way ANOVA test in **C** and **D**; Pearson's correlation test in **B**. * P <0.05, ** P < 0.01, *** P < 0.001.

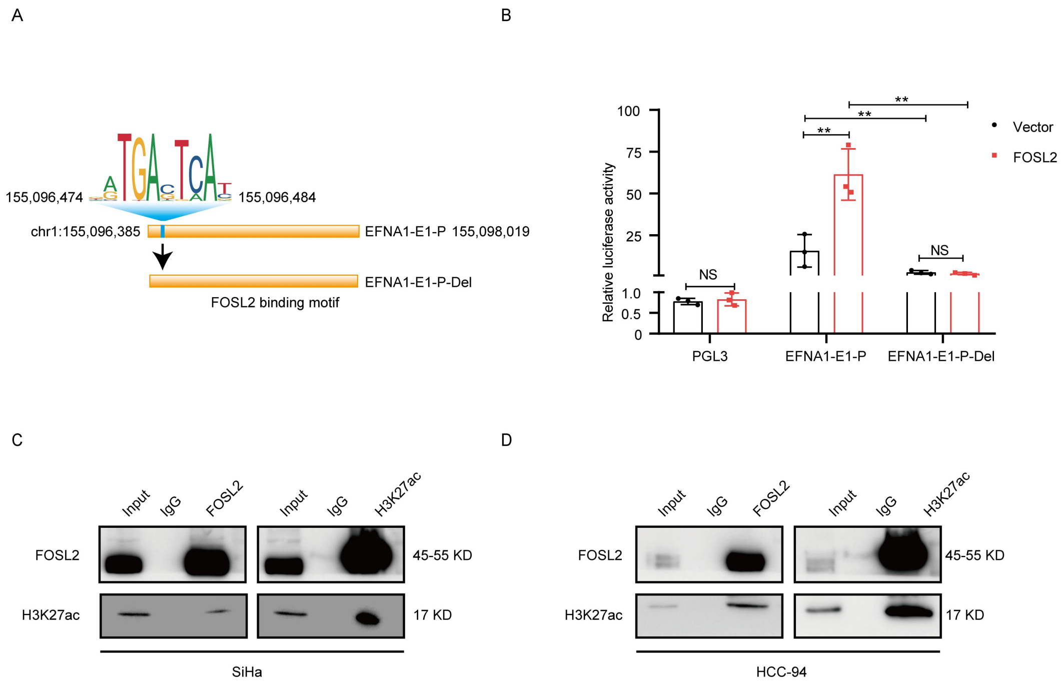

Supplementary Fig 6. Super-enhancer-associated *EFNA1* is transcriptionally activated by FOSL2. (**A**) Schematic diagram illustrating the *EFNA1* E1 region (from chr1:155,096,358–155,098,019) with the FOSL2 binding sequence (from chr1:155,096,474–155,096,484). (**B**) Luciferase reporter assay demonstrating the activity of indicated plasmids in HEK 293T cells. Cells were transfected cells with the specified plasmids for 48 h, and luciferase activity was normalized to pRL-TK luciferase activity. Data are presented as mean ± SD from three independent experiments. One-way ANOVA test is conducted to assess the difference between groups in **B**. * P <0.05, ** P < 0.01, *** P < 0.001. **(C-D)** Western blot analysis of immunoprecipitation products using anti-FOSL2 or anti-H3K27ac antibodies in SiHa (C) and HCC-94 (D) cells.

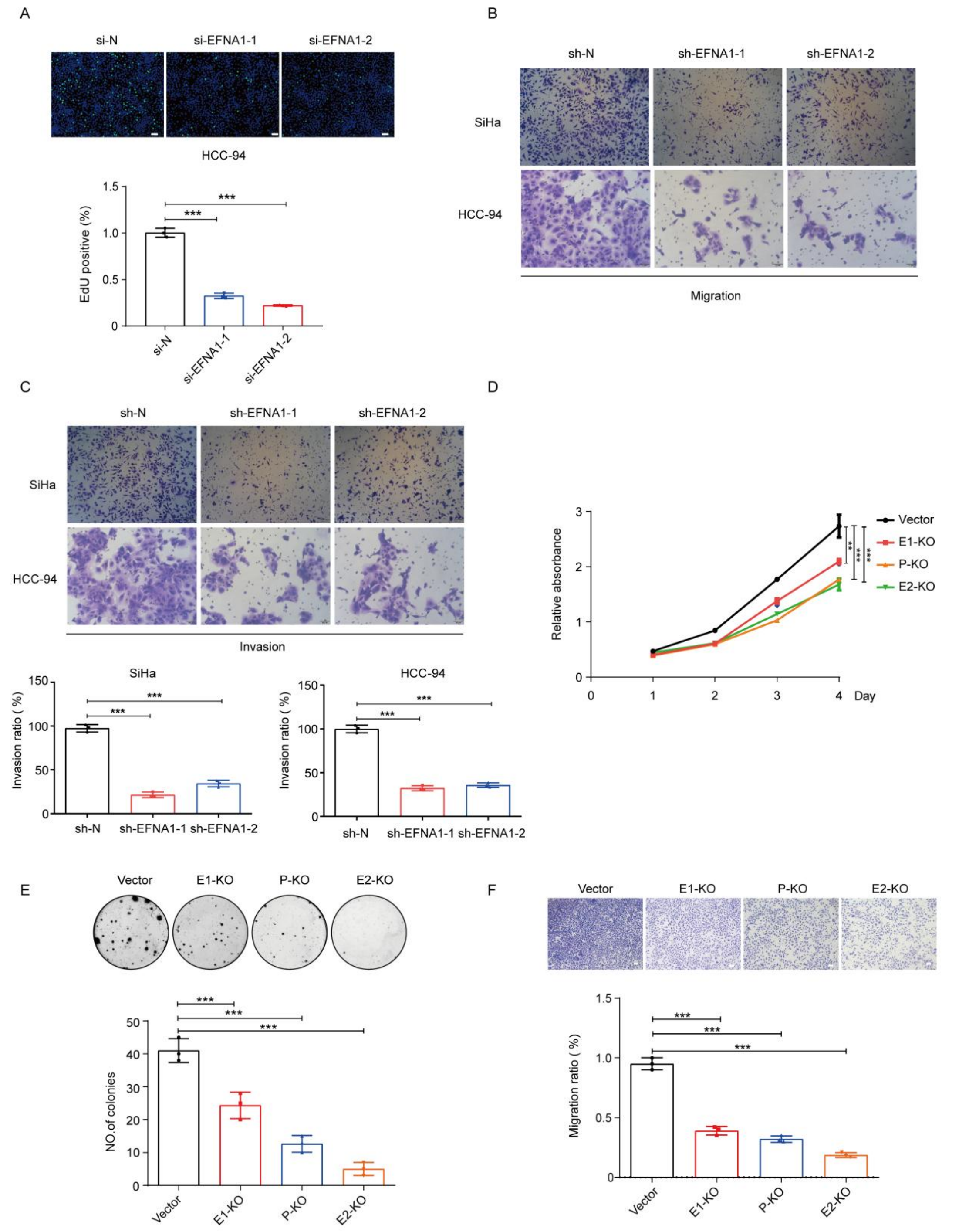

Supplementary Fig 7. EFNA1 Knockdown or *EFNA1*-SE deletions inhibit proliferation and migration in CC cells. (**A**) Representative EdU staining images in HCC-94 cells transfected with either si-N (control) or si-*EFNA1*. Quantification of EdU-positive cells is presented in bar graphs at the bottom. (**B**) Representative images for transwell assay showing the migration ability of SiHa and HCC-94 cells infected with lentivirus expressing EFNA1 shRNAs or control shRNA. (**C**) Transwell assay results showing the invasion ability of cells described in B. The migration

statistics are presented at the bottom. (**D**) CCK-8 assay assessing the proliferation rate of *EFNA1*-SE knockout SiHa cells. (**E**) Colony formation assay results for EFNA1-SE knockout cells described in D. The colony statistics are presented at the bottom. (**F)** Migration ability of EFNA1-SE knockout cells described in D assessed by transwell assays. Images are representative of three independent assays. The statistics are presented at the bottom. Data are presented as mean ± SD from three independent experiments. Statistical analysis: one-way ANOVA test. * P <0.05, ** P < 0.01, *** P < 0.001.

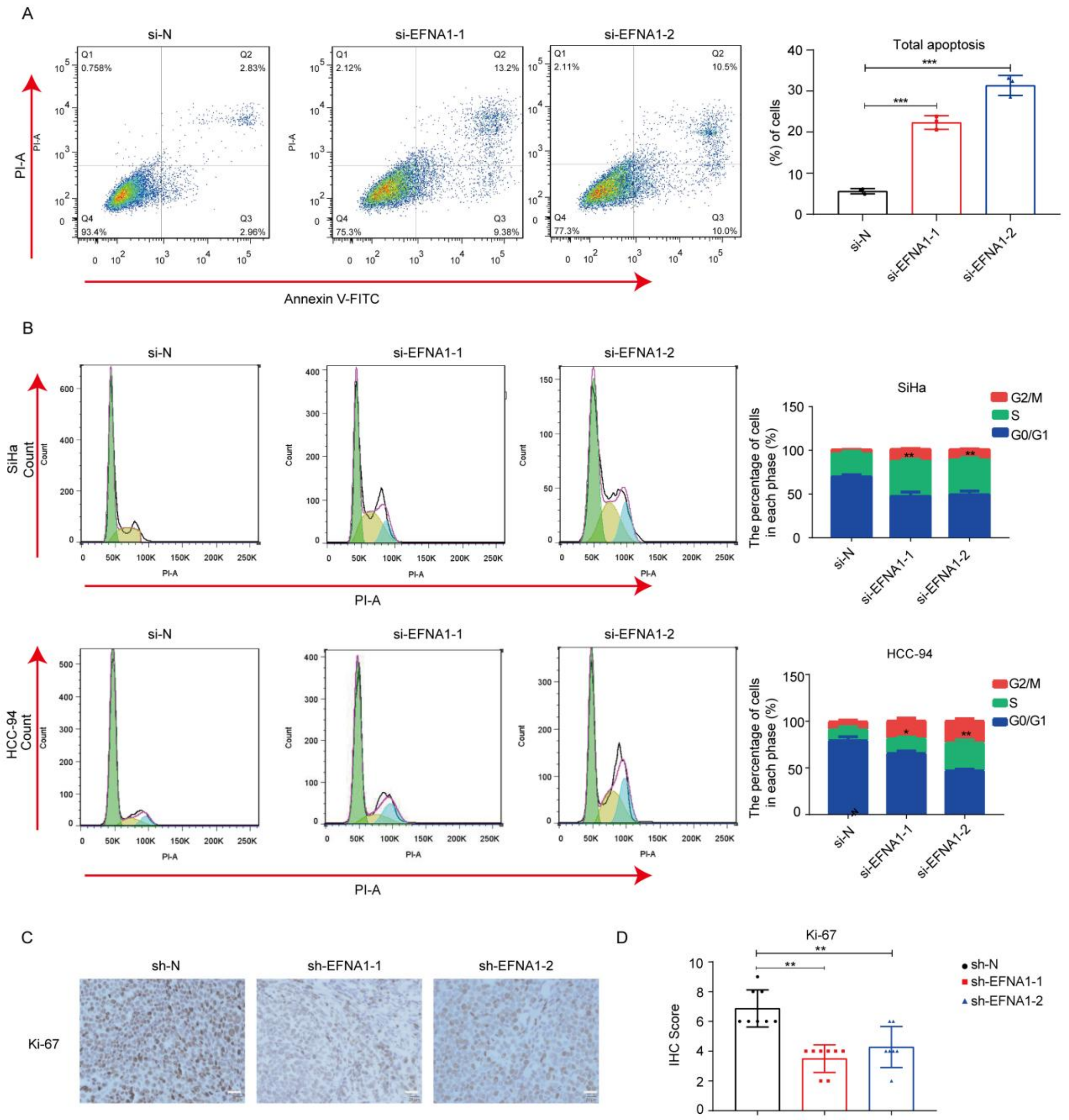

Supplementary Fig 8. EFNA1 knockdown induces apoptosis and G2/M arrest in CC cells. **(A)** Flow cytometry analysis of cell apoptosis using Annexin V-FITC/PI staining in HCC-94 cells transfected with *EFNA1* siRNAs or control siRNA. Quantification is shown as a bar graph on the right. (**B**) Cell cycle dynamics in SiHa and HCC-94 cells, post-transfection with either si-N (control) or si-*EFNA1*, analyzed by using flow cytometry. Quantification is presented as a bar graph on the right. (**C**) IHC staining of xenografts tumors using Ki-67 antibodies. Micro-vascular densities in the tumors from the three indicated groups were quantified based on Ki-67 staining (n = 8), with results shown in the bar plot on the right. Data are presented as mean ± SD from three independent experiments. Statistical analysis: one-way ANOVA test. * P <0.05, ** P < 0.01, ***

$P < 0.001$.

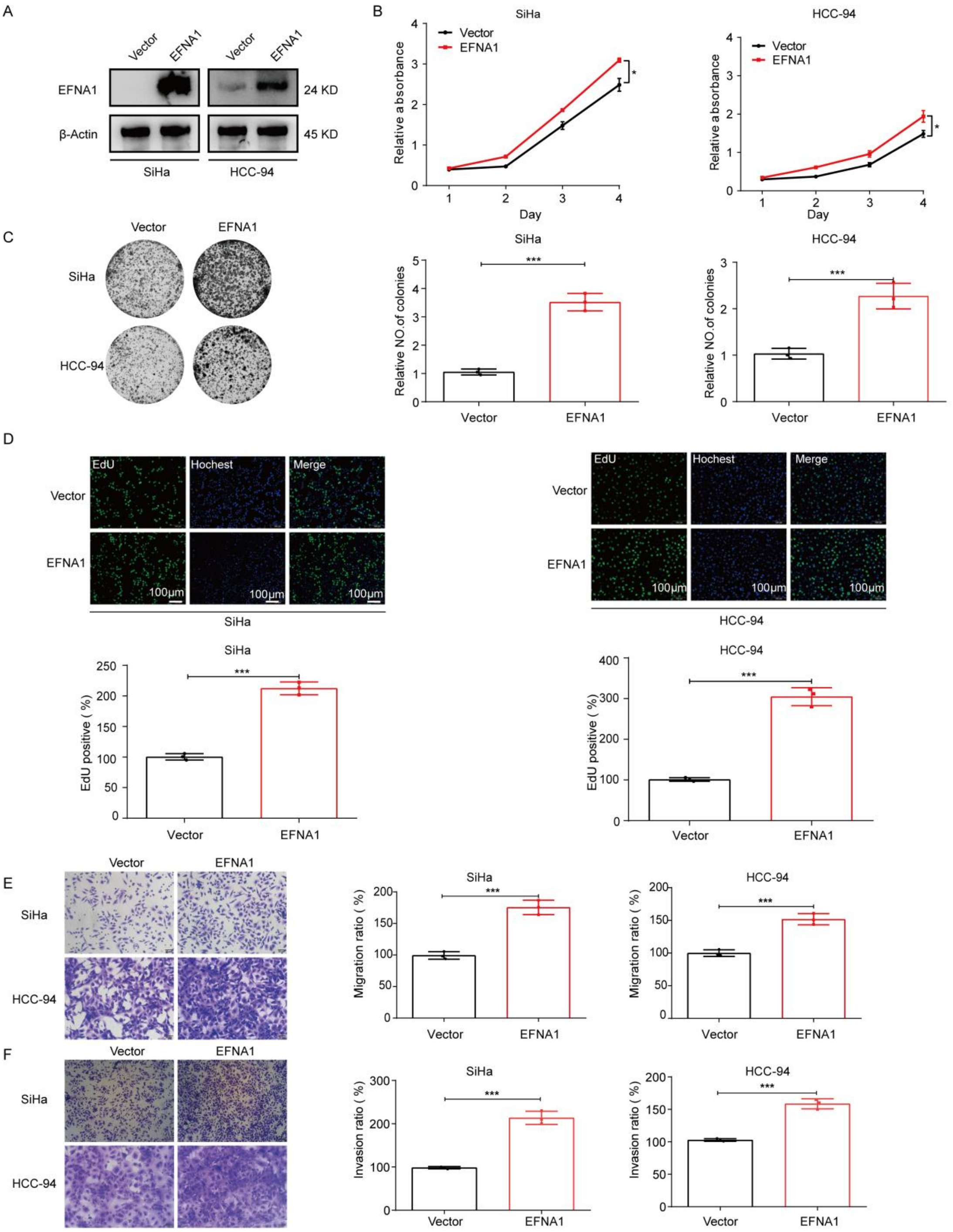

Supplementary Fig 9. EFNA1 promotes the proliferation and migration of CC cells *in vitro*. (A) Western blot analysis confirming EFNA1 overexpression in SiHa and HCC-94 cells. β-Actin is used as a loading control. (**B**) CCK-8 assay indicating the growth rate of the EFNA1-overexpressing CC cells described in A. Absorbance from day 1 to day 4 is normalized to day 0 (set as 100% control). (**C**) Representative images of colony formation assay for siHa and

HCC-94 cells described in A. Colony numbers are quantified and presented in the bar plots on the right. (**D**) Representative images of EdU staining assay for siHa and HCC-94 cells described in A. The percentage of EdU positive cells is shown in the bar graphs on the right. (**E**) Representative images of transwell assay for the cells described in A. Summary statistics of migration are shown in the bar plots on the right. (**F**) Transwell assays showing the invasion ability of cells described in A, with statistics presented at the right. Scale bar, 100 μm. Data in the bar plots are presented as the mean ± SD from three independent experiments. Statistical analysis: t-test. * P <0.05, ** P < 0.01, *** P < 0.001.

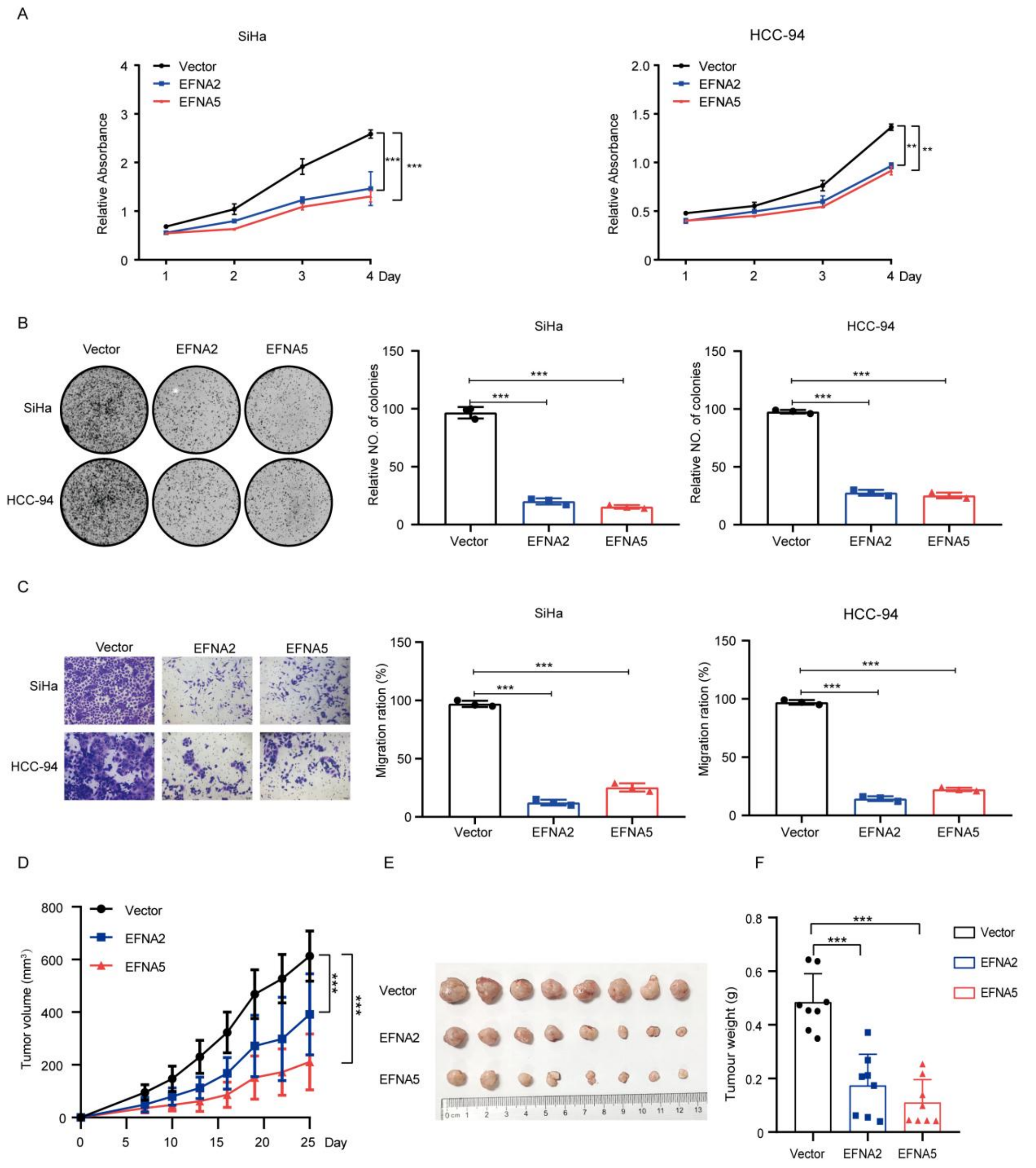

Supplementary Fig 10. EFNA2 and EFNA5 demonstrate tumor suppressive effects in cervical cancer. (A) CCK-8 assay evaluating the proliferation abilities of CC cells infected with lentivirus overexpressing EFNA2, EFNA5, or control vectors. Absorbance from Days 1-4 is normalized to that from Day 0. **(B-C)** Representative images of the colony formation assay (B) and transwell migration assay (C) for CC cells described in (A). Statistical results are presented on the right. **(D-F)** Measurements for tumorigenesis in nude mice subcutaneously injected with SiHa cells stably expressing EFNA2, EFNA5 or control vectors. Tumor volumes were recorded every 3 days. The tumors extracted from the mice were photographed (**E**) and weighted (**F**) after sacrifice.

The corresponding statistical analysis of tumor weight are presented in the bar graph. Scale bar, 100 µm. Statistical analysis: one-way ANOVA test. * P <0.05, ** P < 0.01, *** P < 0.001.

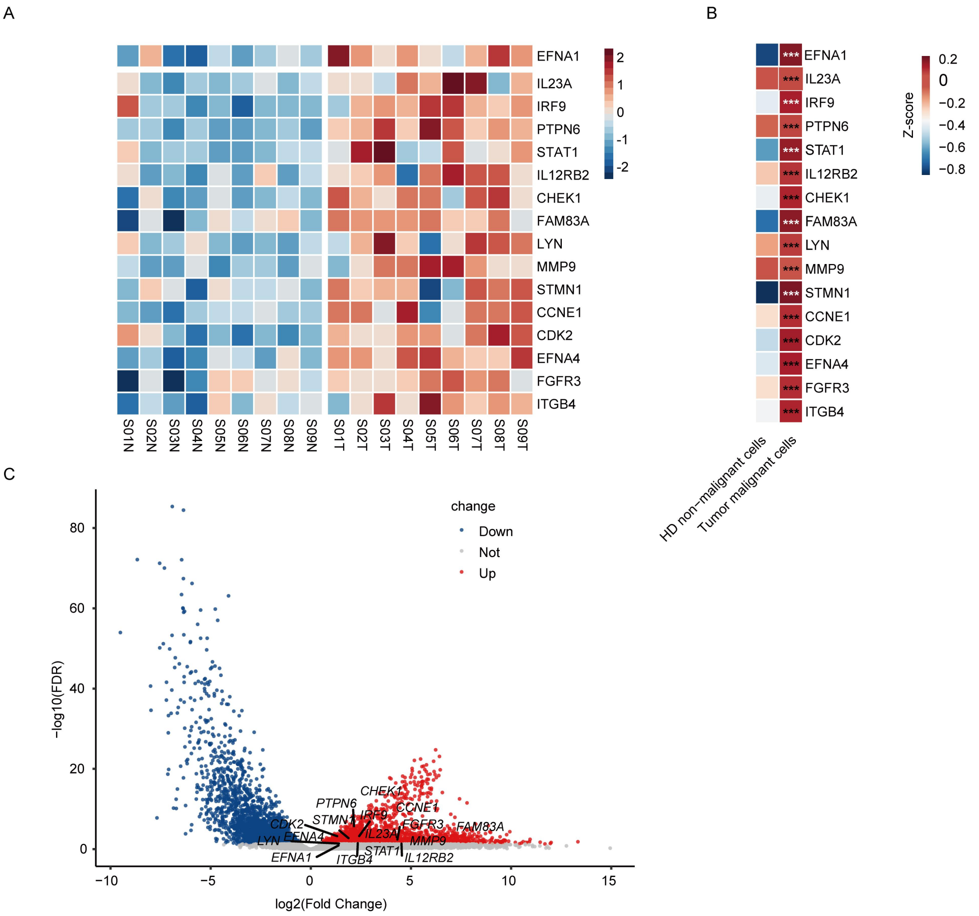

Supplementary Fig 11. Key genes in the Src/AKT/STAT3 pathway are upregulated in CC tumors. (**A**) Heatmap illustrating the expression levels of key genes within the Src/AKT/STAT3 pathway (rows) across CC tumors (T=9) and paired adjacent normal (N=9) tissues (columns). The color gradient from blue to red indicates low to high transcription levels. (**B**) Heatmap depicting the expression levels of key genes in the Src/AKT/STAT3 pathway in CC tumor cells and their microenvironments using the single-cell transcriptomic data (N=3, T=14). The color gradient from blue to red indicates low to high transcription levels. ***P < 0.001. The statistical significance was assessed using the wilcoxon rank-sum test. (**C**) Volcano plot showing differentially expressed genes (DEG) in the TCGA-CESC cohort using the edgeR package (version 3.36.0). P-values were adjusted for multiple testing using the false discovery rate (FDR) approach. Upregulated genes with a fold change >1.5 are highlighted in red, while downregulated genes with a fold change <-1.5, are shown in blue (FDR < 0.05; N=13, T=306).

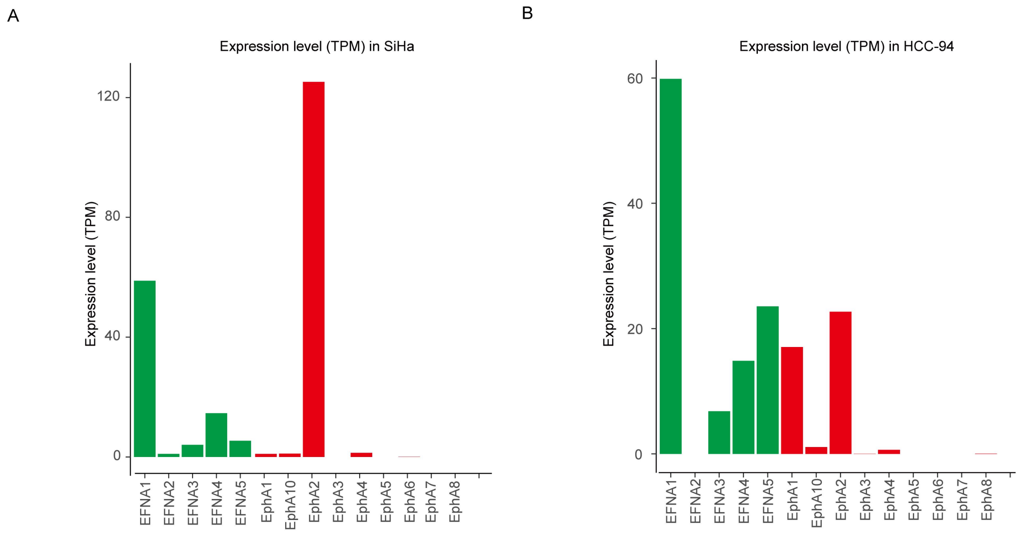

Supplementary Fig 12. Expression level of Eph-ephrin network genes in cervical cancer cells. (**A**) Transcriptome analysis showing the gene expression of EFNAs and EphA receptors in SiHa (A) and HCC-94 cell lines (**B**). TPM strands for transcripts per million.

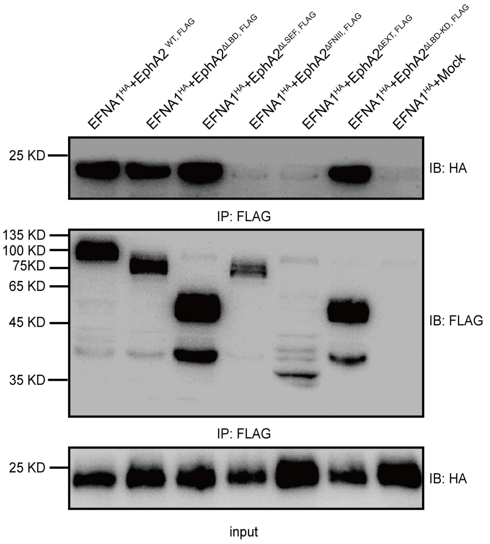

Supplementary Fig 13. Cis-interaction between EFNA1 and EphA2. Co-immunoprecipitation (Co-IP) assays in SiHa cells co-transfected with EFNA1^{HA} and either EphA2$^{WT, FLAG}$, EphA2$^{\triangle LSEF, FLAG}$, EphA2$^{\triangle LBD, FLAG}$, EphA2$^{\Delta FNIII, FLAG}$, EphA2$^{\triangle EXT, FLAG}$, EphA2$^{\triangle LBD\text{-}KD, FLAG}$, or control plasmids. Immunoprecipitations were performed using anti-Flag antibodies.

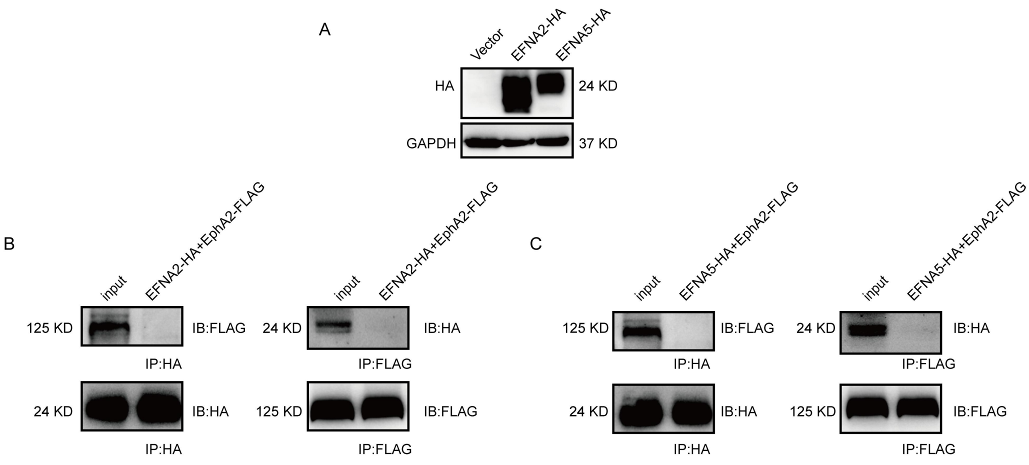

Supplementary Fig 14. EphA2 does not interact with EFNA2 or EFNA5. (**A**) Western blot analysis confirming the overexpression of HA-tagged EFNA2 or EFNA5 in 293T cells. (**B-C**) Co-IP assays evaluating the interaction between EphA2 and EFNA2 (B) or EFNA5 (C). Both forward (IP: HA, IB: FLAG; left panels) and reverse pull-down (IP: FLAG, IB: HA; right panels) approaches were used, showing no detectable interaction between EphA2 and either EFNA2 or EFNA5.

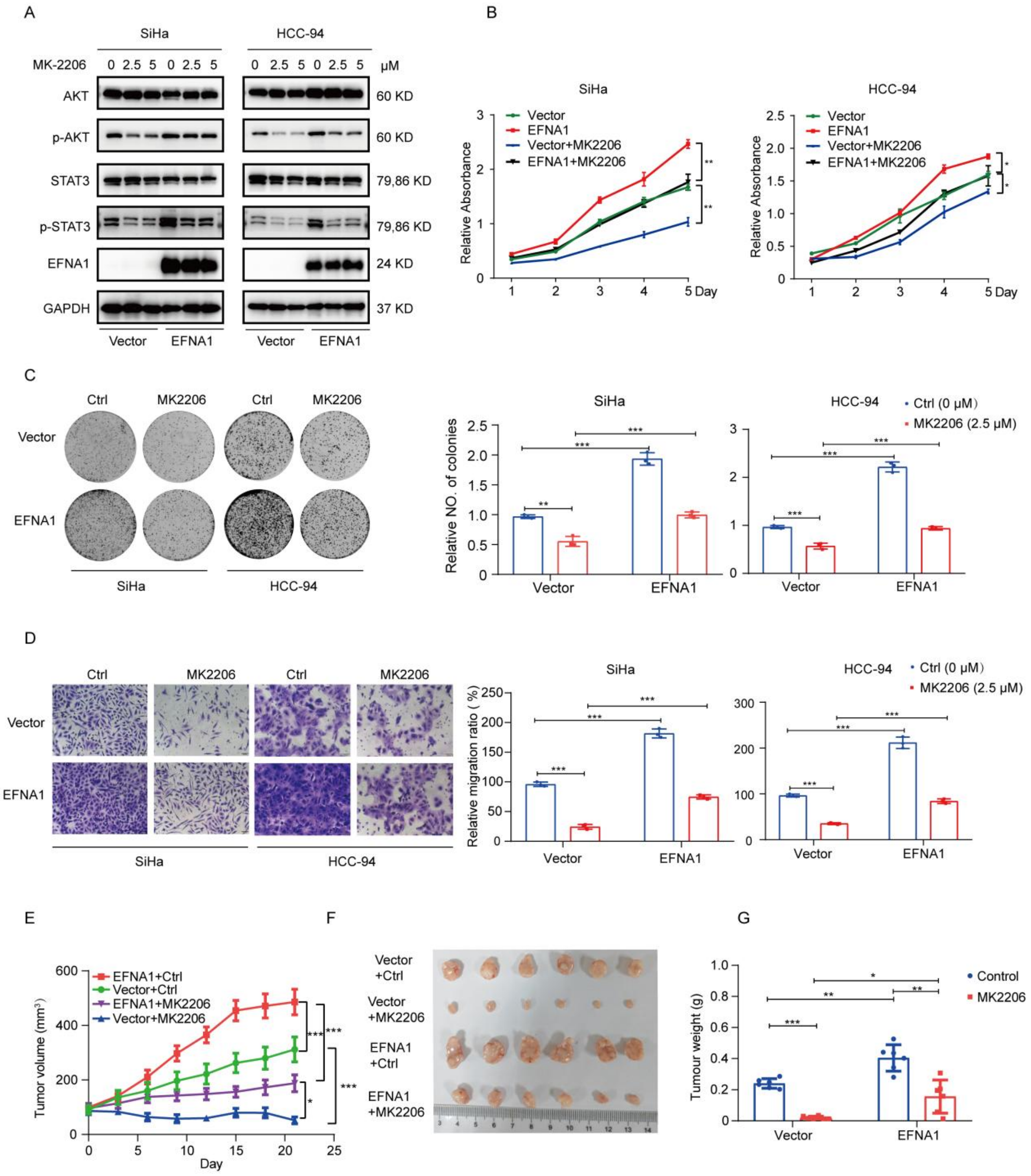

Supplementary Fig 15. MK2206 mitigates *EFNA1*-driven tumor progression in cervical cancer. (A) Western blot showing protein levels of the AKT/STAT3 pathway in SiHa and HCC-94 cells with or without *EFNA1* overexpression and MK2206 treatment. GAPDH serves as a loading control. (**B**) CCK8 assay showing cell proliferation curves for cells described in (A). (**C**) Colony formation assay for cells described in (A), with statistical results presented in the bar graph on the right. (**D**) Transwell migration assay showing the migration ability of cells described in (A), with statistical results indicated on the right. (E-G) Measurements for tumorigenesis in nude mice subcutaneously injected with HCC-94 cells stably expressing EFNA1 or control vectors, followed

by MK2206 or control vehicle treatment. Tumor volumes were recorded every 3 days (**E**). The tumors extracted from mice were photographed (**F**) and weighted (**G**) after sacrifice. The corresponding statistics are presented in the bar graph. Scale bar, 100 μm. Statistical significance levels: Between-group comparisons: one-way ANOVA test. * $P < 0.05$, ** $P < 0.01$, *** $P < 0.001$.

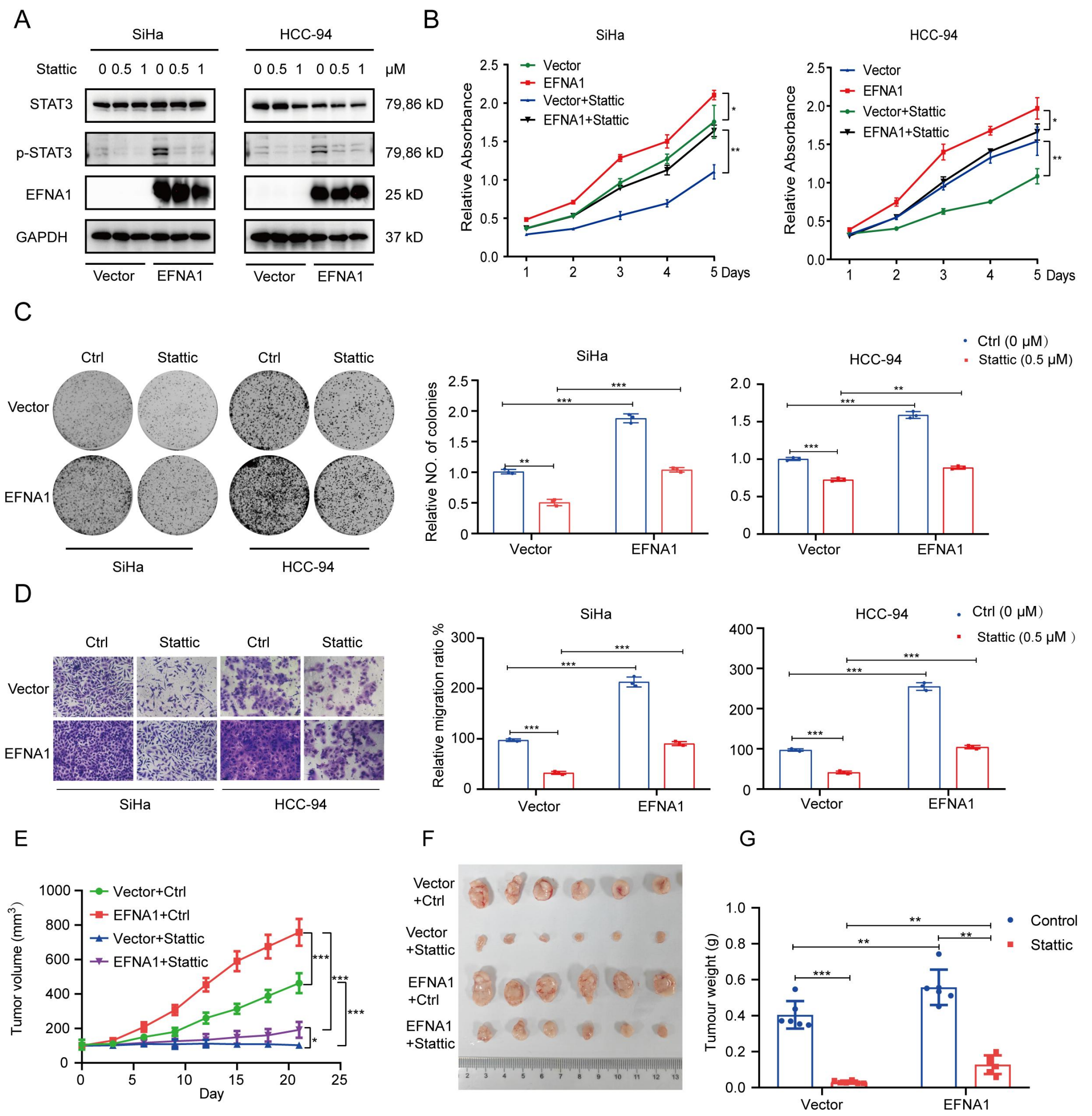

Supplementary Fig 16. Stattic mitigates *EFNA1*-driven tumor progression in cervical cancer. **(A)** Western blot showing protein levels of STAT3 in SiHa and HCC-94 cells with or without EFNA1 overexpression and Stattic treatment. GAPDH serves as a loading control. (**B**) CCK8 assay showing cell proliferation curves for cells described in (A). (**C**) Colony formation assay for cells described in (A), with statistical results presented in the bar graph on the right. (**D**) Transwell assay showing migration ability for cells described in (A), with statistical results presented in the bar graph on the right. (E-G) Measurements for tumorigenesis in nude mice subcutaneously injected with HCC-94 cells stably expressing EFNA1 or control vectors, followed by Stattic or control vehicle treatment. Tumor volumes were recorded every 3 days (**E**). The tumors extracted from mice described in E were photographed (**F**) and weighted (**G**) after sacrifice. The

corresponding statistics are presented on the right. Scale bar, 100 μm. Between-group comparisons: one-way ANOVA test. Statistical significance levels: * $P < 0.05$, ** $P < 0.01$, *** $P < 0.001$.

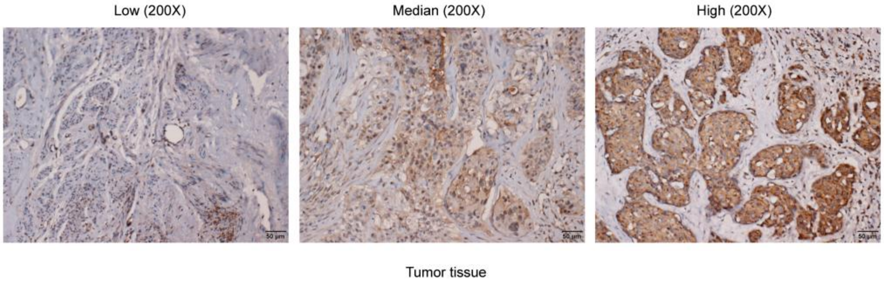

Supplementary Fig 17. **Representative images of IHC staining for EFNA1 expression levels in CC tumors.** Shown are samples with low, median, and high expression levels in 200X magnifications. Scale bar, 50 μm.

References

1. Tang Z, Kang B, Li C, Chen T, and Zhang Z. GEPIA2: an enhanced web server for large-scale expression profiling and interactive analysis. *Nucleic Acids Res.* 2019;47(W1):W556-w60.
2. Fan J, Lu F, Qin T, Peng W, Zhuang X, Li Y, et al. Multiomic analysis of cervical squamous cell carcinoma identifies cellular ecosystems with biological and clinical relevance. *Nat Genet.* 2023;55(12):2175-88.
3. Stuart T, Butler A, Hoffman P, Hafemeister C, Papalexi E, Mauck WM, 3rd, et al. Comprehensive integration of single-cell data. *Cell.* 2019;177(7):1888-902.e21.
4. Langmead B, and Salzberg SL. Fast gapped-read alignment with Bowtie 2. *Nature methods.* 2012;9(4):357-9.
5. Dobin A, Davis CA, Schlesinger F, Drenkow J, Zaleski C, Jha S, et al. STAR: ultrafast universal RNA-seq aligner. *Bioinformatics (Oxford, England).* 2013;29(1):15-21.
6. Li B, and Dewey CN. RSEM: accurate transcript quantification from RNA-Seq data with or without a reference genome. *BMC bioinformatics.* 2011;12:323.
7. Love MI, Huber W, and Anders S. Moderated estimation of fold change and dispersion for RNA-seq data with DESeq2. *Genome biology.* 2014;15(12):550.
8. Li H, and Durbin R. Fast and accurate short read alignment with Burrows-Wheeler transform. *Bioinformatics (Oxford, England).* 2009;25(14):1754-60.
9. Ramírez F, Bhardwaj V, Arrigoni L, Lam KC, Grüning BA, Villaveces J, et al. High-resolution TADs reveal DNA sequences underlying genome organization in flies. *Nat Commun.* 2018;9(1):189.
10. Heinz S, Benner C, Spann N, Bertolino E, Lin YC, Laslo P, et al. Simple combinations of lineage-determining transcription factors prime cis-regulatory elements required for macrophage and B cell identities. *Molecular cell.* 2010;38(4):576-89.
11. Wu T, Hu E, Xu S, Chen M, Guo P, Dai Z, et al. clusterProfiler 4.0: A universal enrichment tool for interpreting omics data. *Innovation (Camb).* 2021;2(3):100141.
12. Luo ZH, Shi MW, Zhang Y, Wang DY, Tong YB, Pan XL, et al. CenhANCER: a comprehensive cancer enhancer database for primary tissues and cell lines. *Database (Oxford).* 2023;2023:baad022.
