# Supplementary material for: Super-enhancer–driven EFNA1 fuels tumor progression in cervical cancer via the FOSL2-Src/AKT/STAT3 axis
Source: J Clin Invest. 2025 Feb 18;135(8):e177599. doi: 10.1172/JCI177599 (PMC11996870; doi:10.1172/JCI177599)

Fig.2D

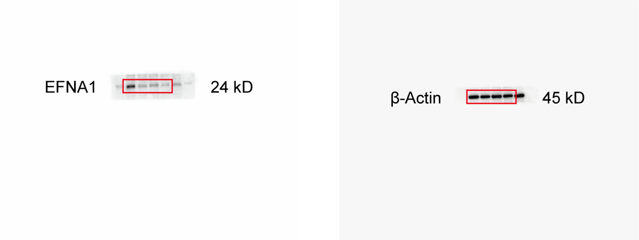

Fig.2G

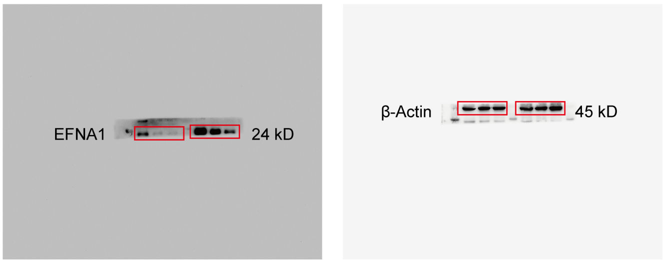

Fig.3D

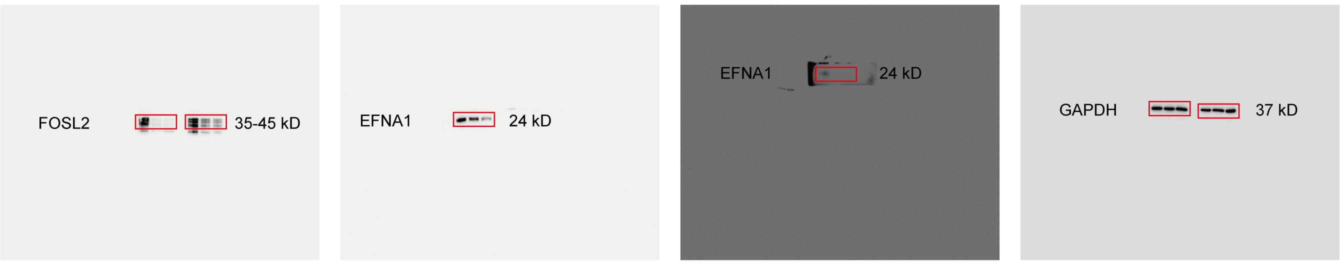

Fig.3E

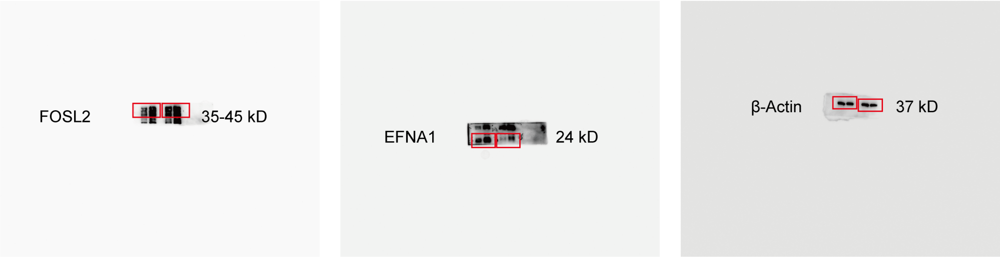

Fig.4A

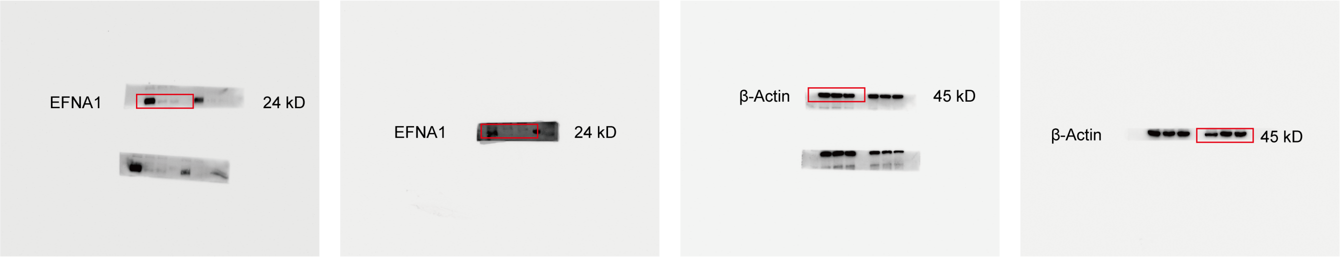

Fig 5C

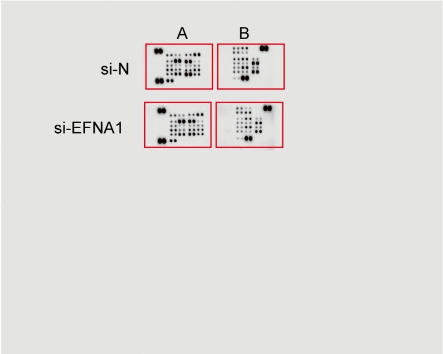

Fig 5E

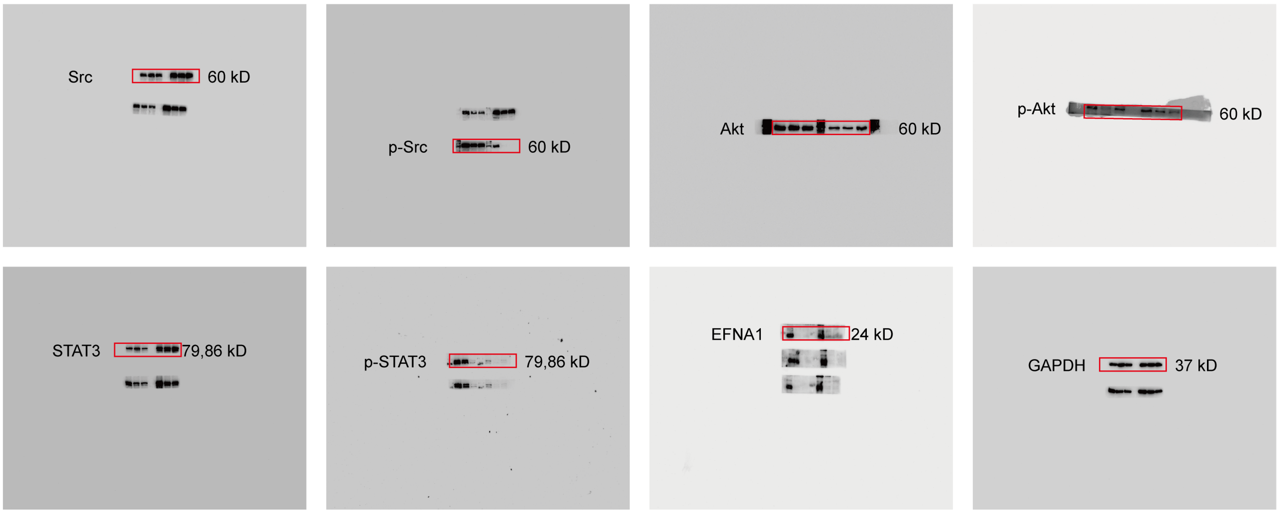

Fig 5F

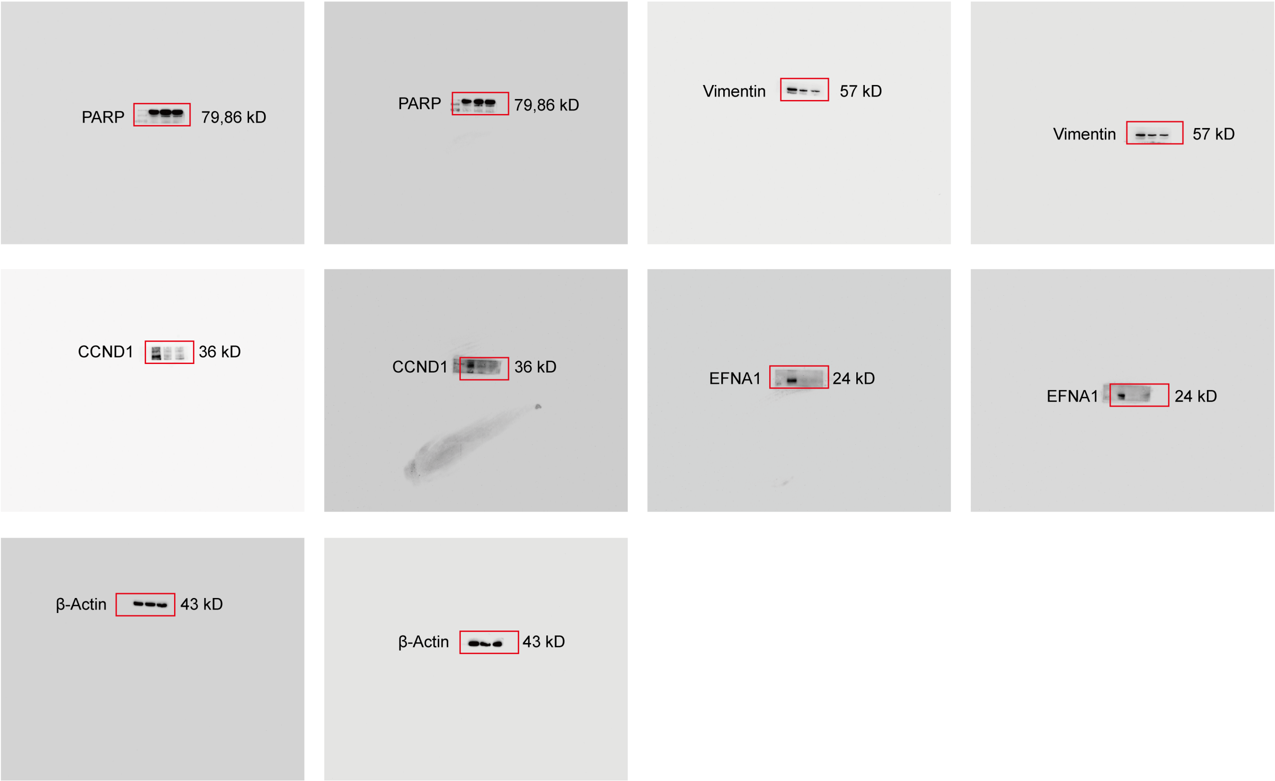

Fig 5G

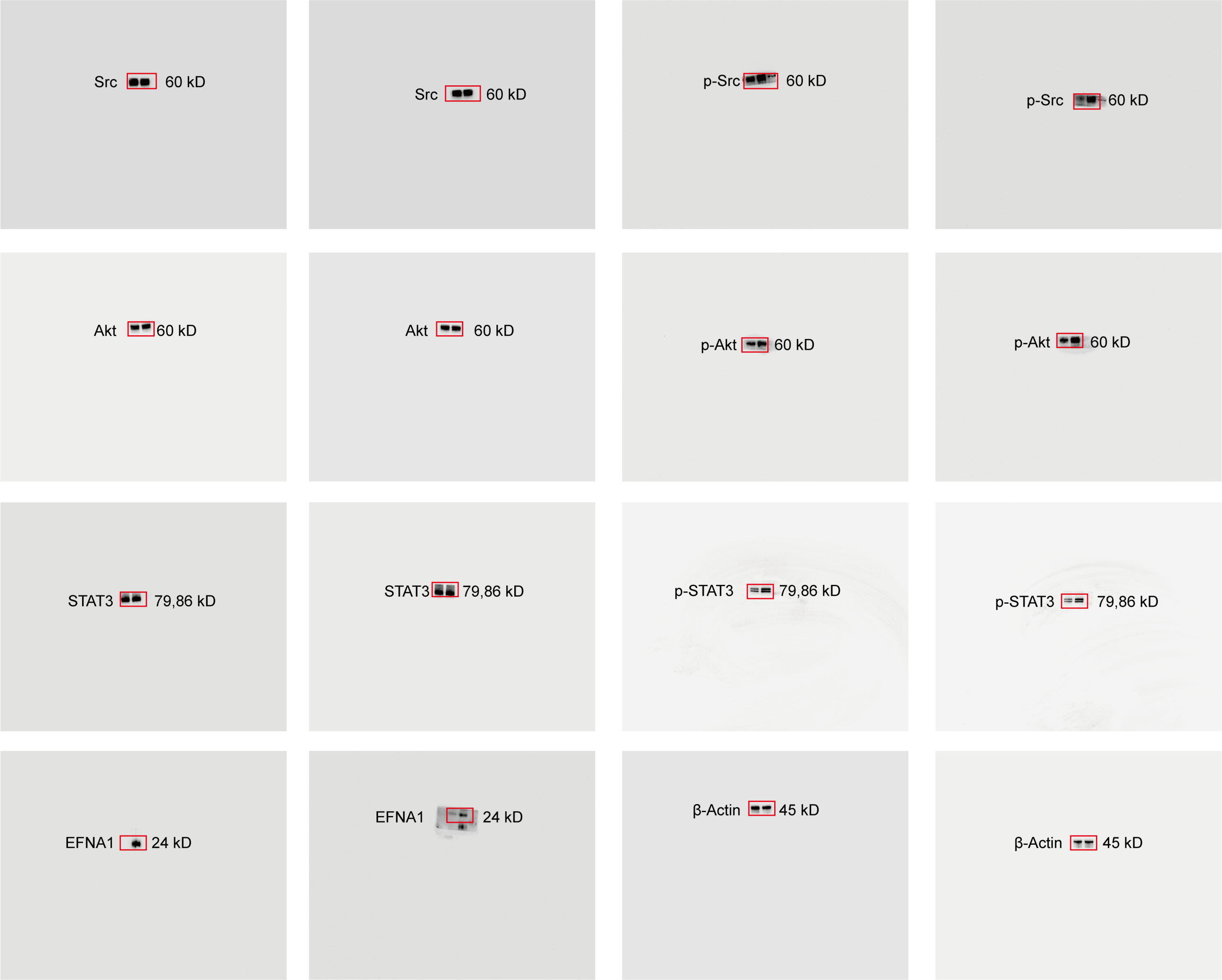
Src 60 kD
Src 60 kD
p-Src 60 kD
p-Src 60 kD
Akt 60 kD
Akt 60 kD
p-Akt 60 kD
p-Akt 60 kD
STAT3 79,86 kD
STAT3 79,86 kD
p-STAT3 79,86 kD
p-STAT3 79,86 kD
EFNA1 24 kD
EFNA1 24 kD
β-Actin 45 kD
β-Actin 45 kD

Fig 5H

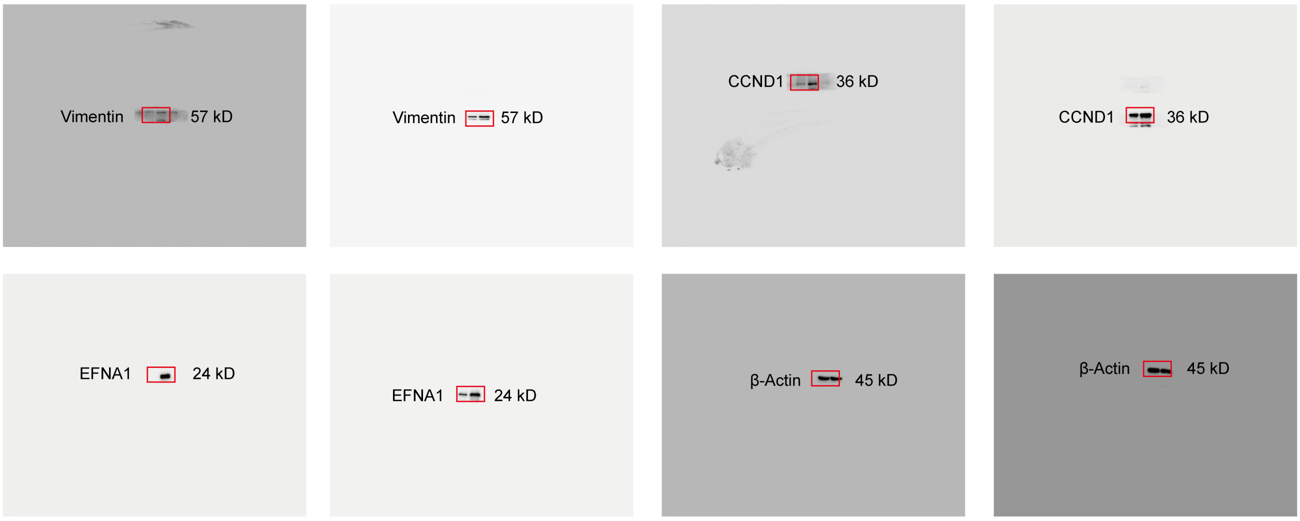
Vimentin 57 kD
Vimentin 57 kD
CCND1 36 kD
CCND1 36 kD
EFNA1 24 kD
EFNA1 24 kD
β-Actin 45 kD
β-Actin 45 kD

Fig 6A

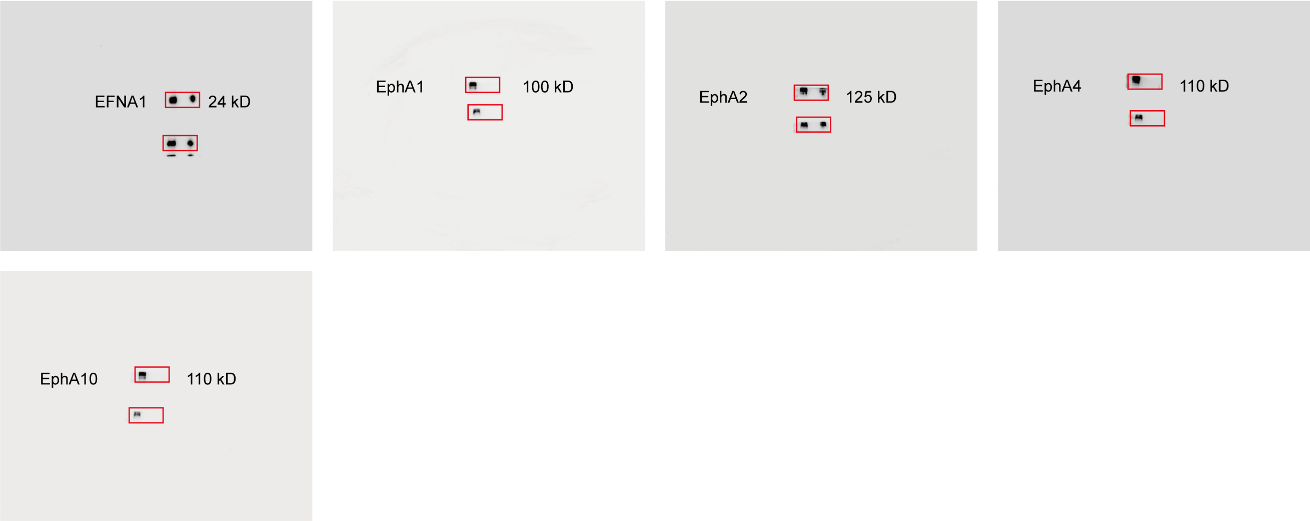

Fig 6B

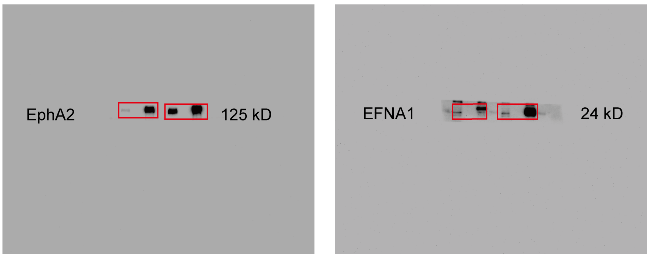

Fig 6D

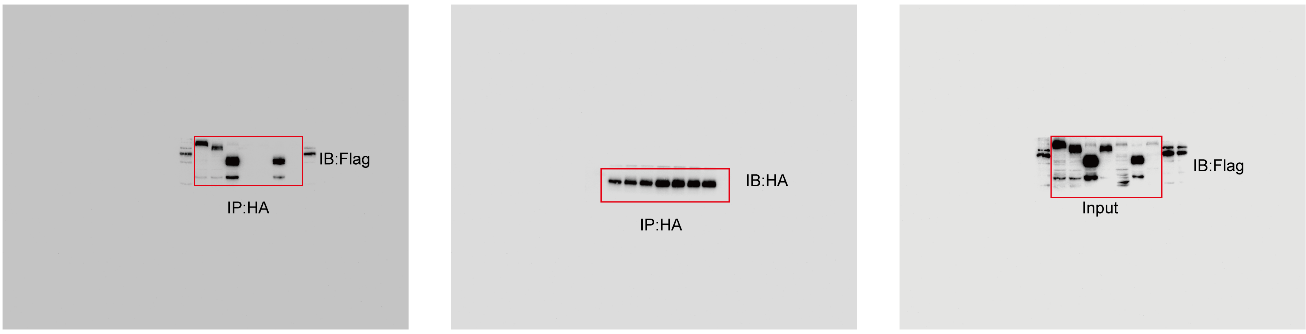

Fig 6E

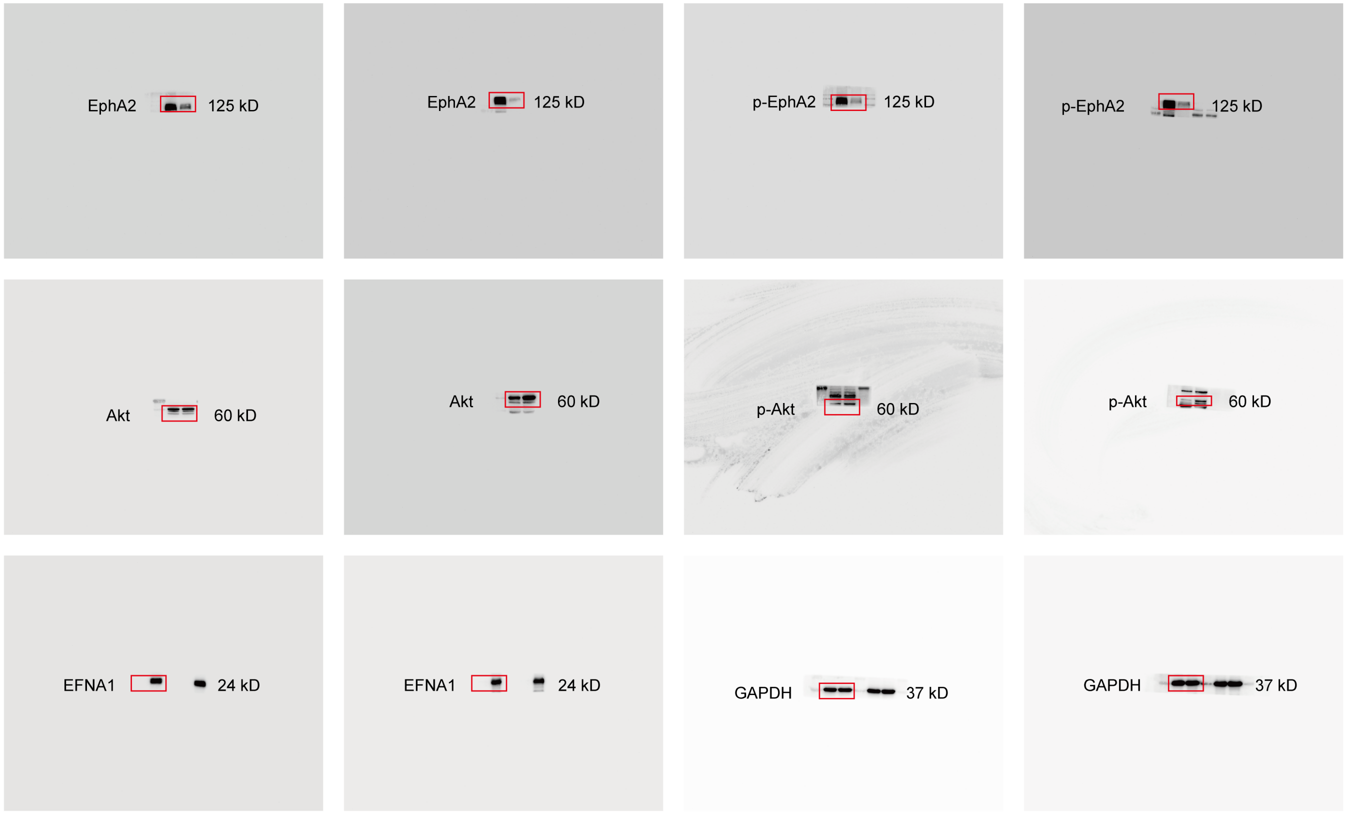
EphA2
125 kD
EphA2
125 kD
p-EphA2
125 kD
p-EphA2
125 kD
Akt
60 kD
Akt
60 kD
p-Akt
60 kD
p-Akt
60 kD
EFNA1
24 kD
EFNA1
24 kD
GAPDH
37 kD
GAPDH
37 kD

Fig 6F

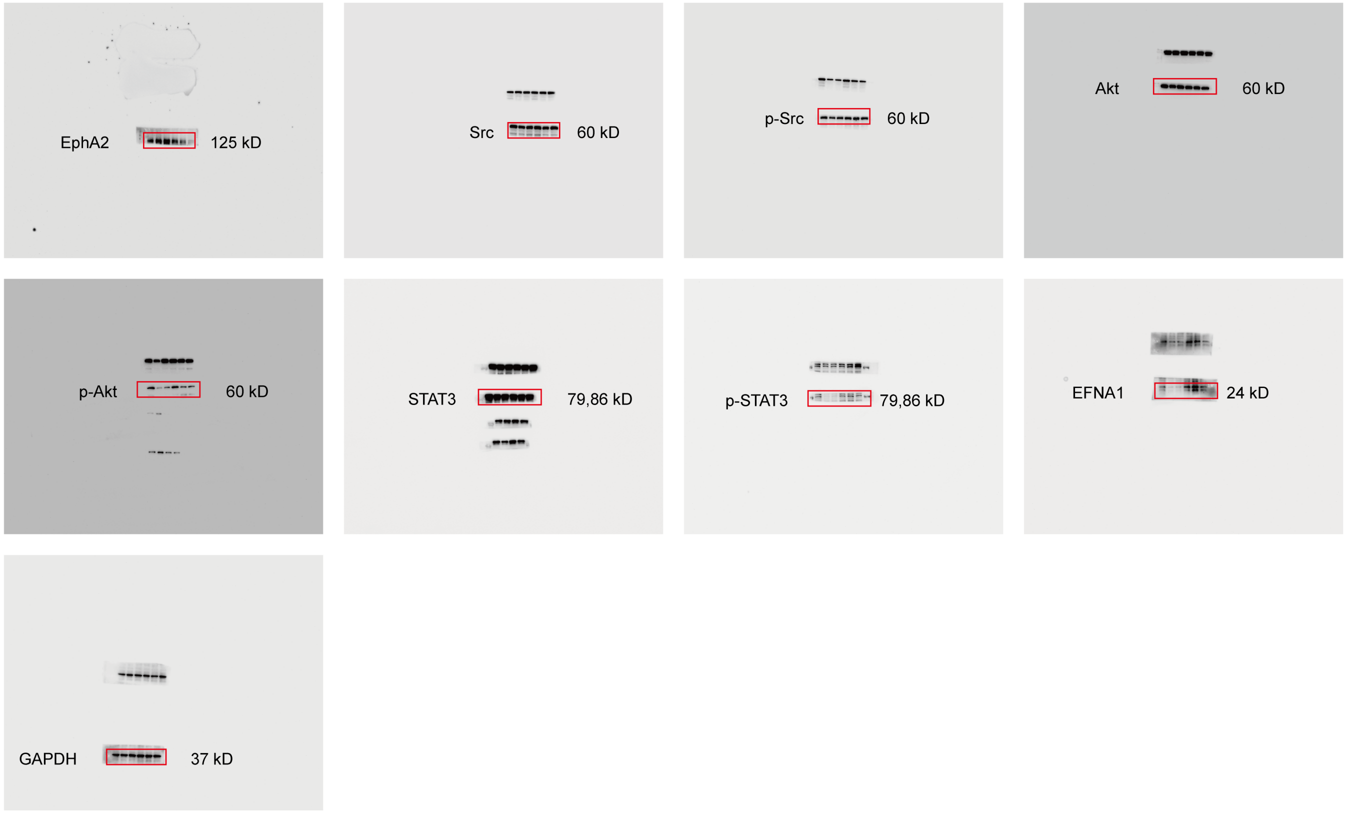
EphA2
125 kD
Src
60 kD
p-Src
60 kD
Akt
60 kD
p-Akt
60 kD
STAT3
79,86 kD
p-STAT3
79,86 kD
EFNA1
24 kD
GAPDH
37 kD

Fig 6G

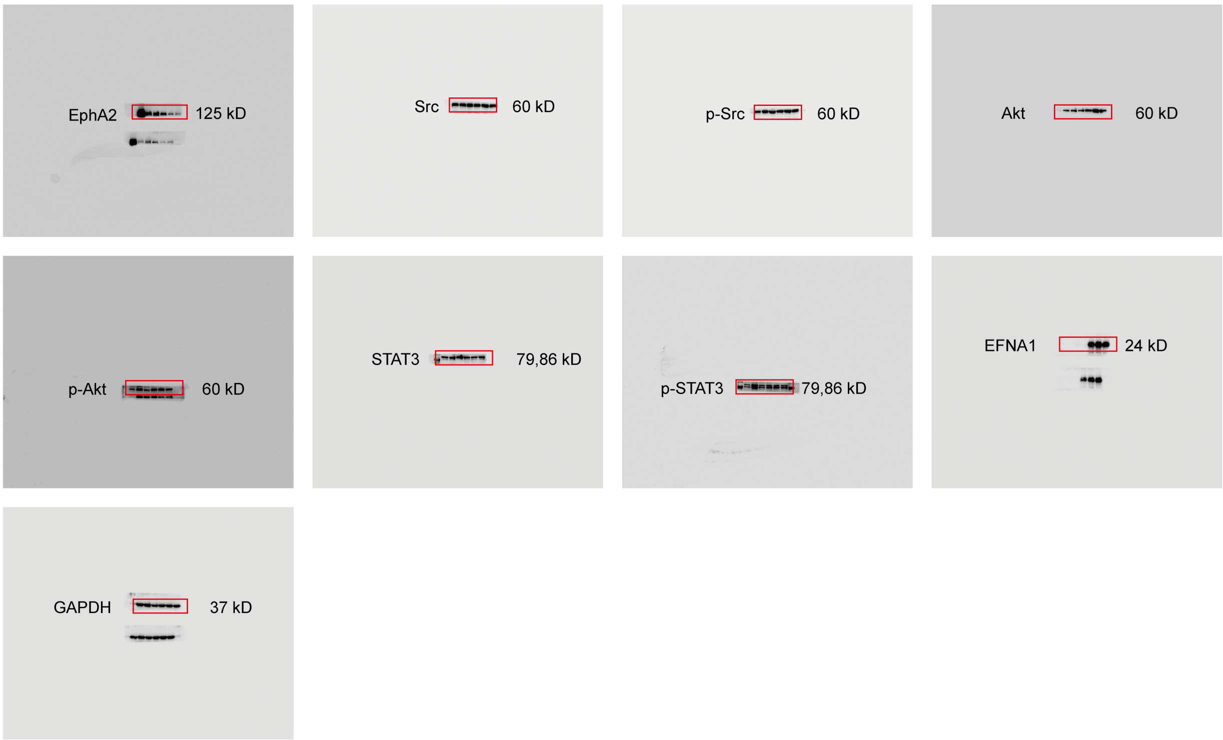
EphA2
125 kD
Src
60 kD
p-Src
60 kD
Akt
60 kD
p-Akt
60 kD
STAT3
79,86 kD
p-STAT3
79,86 kD
EFNA1
24 kD
GAPDH
37 kD

Fig 6H

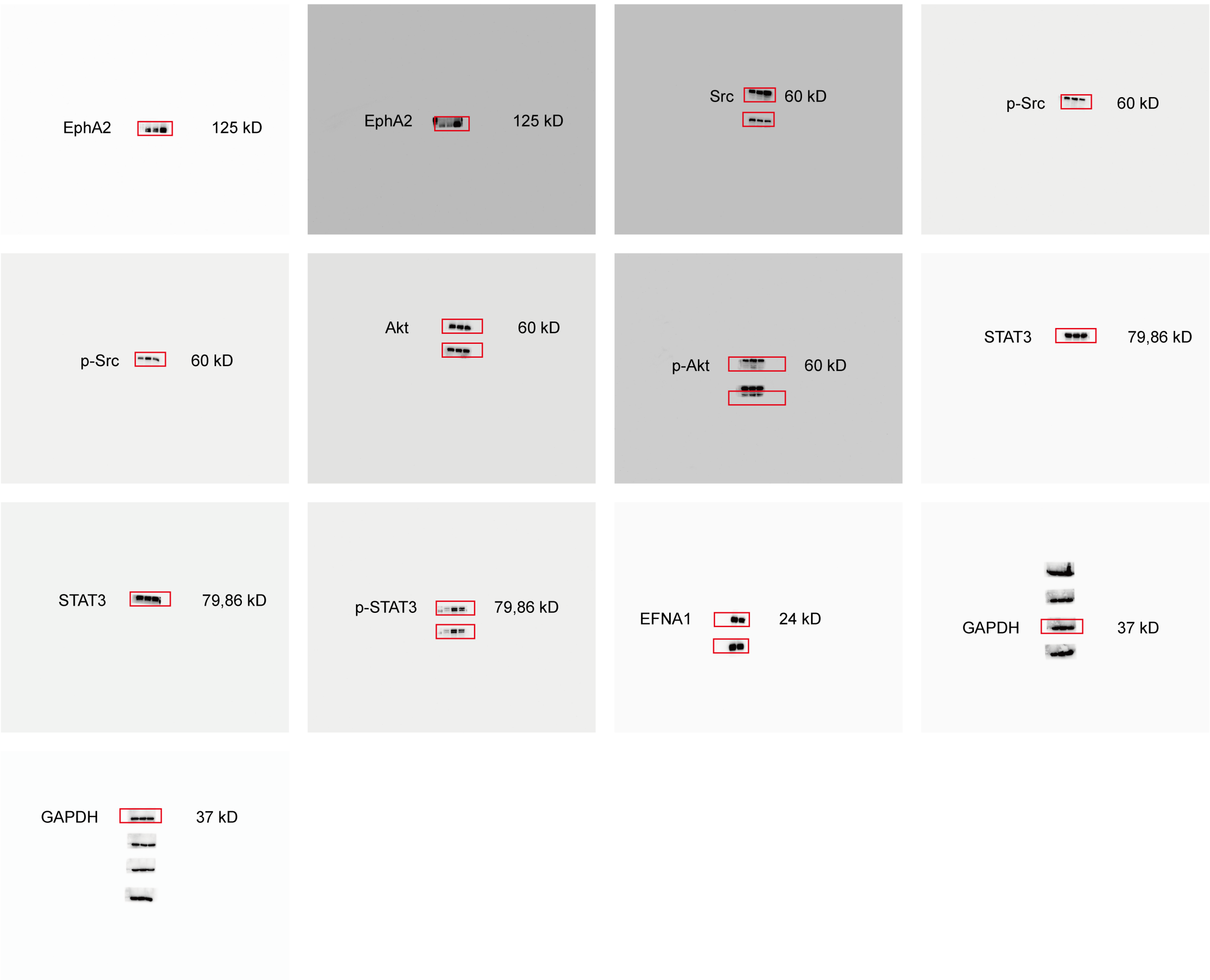
EphA2
125 kD
EphA2
125 kD
Src
60 kD
p-Src
60 kD
p-Src
60 kD
Akt
60 kD
p-Akt
60 kD
STAT3
79,86 kD
STAT3
79,86 kD
p-STAT3
79,86 kD
EFNA1
24 kD
GAPDH
37 kD
GAPDH
37 kD

Fig 7A

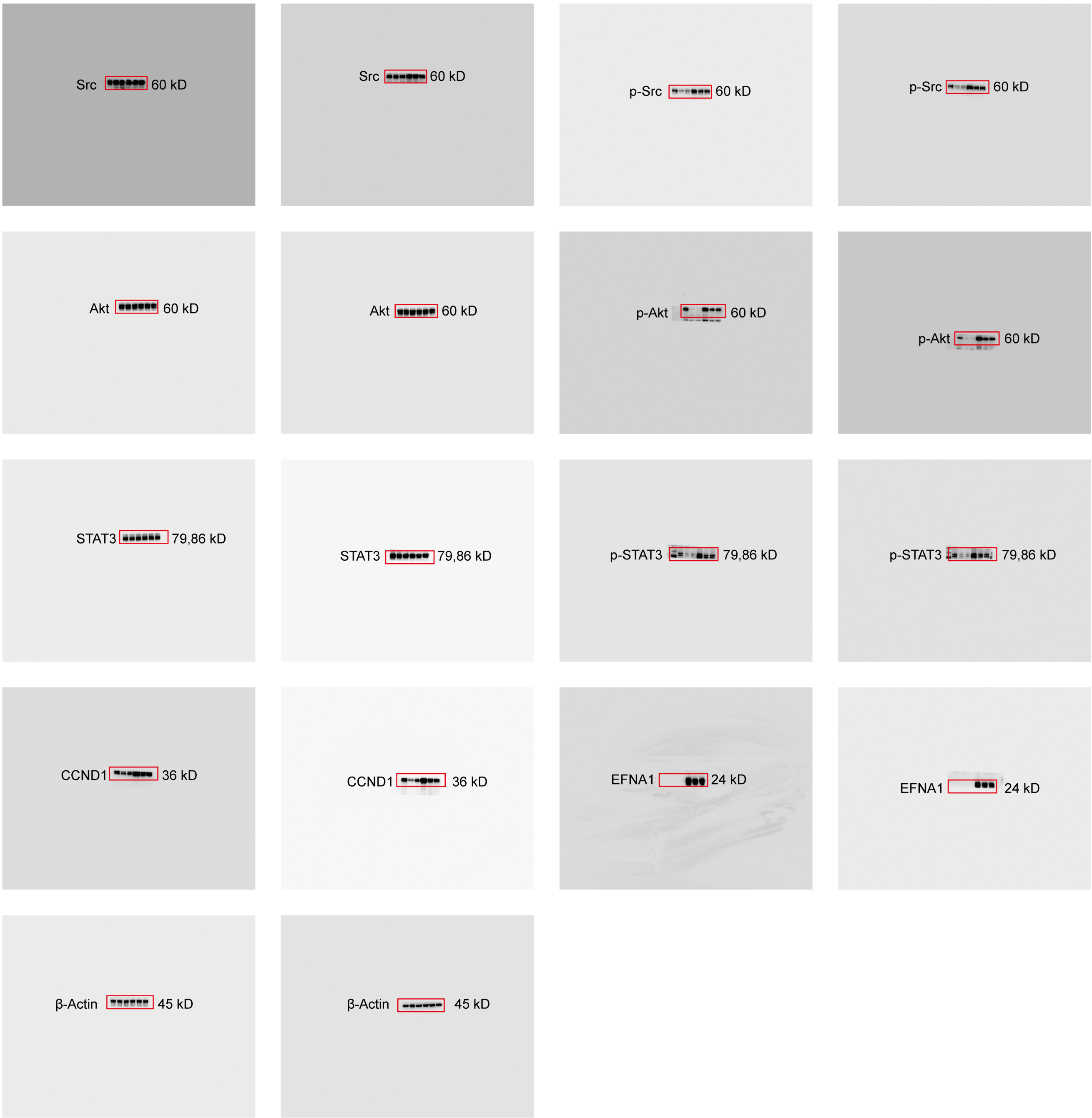
Src 60 kD
Src 60 kD
p-Src 60 kD
p-Src 60 kD
Akt 60 kD
Akt 60 kD
p-Akt 60 kD
p-Akt 60 kD
STAT3 79,86 kD
STAT3 79,86 kD
p-STAT3 79,86 kD
p-STAT3 79,86 kD
CCND1 36 kD
CCND1 36 kD
EFNA1 24 kD
EFNA1 24 kD
β-Actin 45 kD
β-Actin 45 kD

Supplementary Fig 6 D E

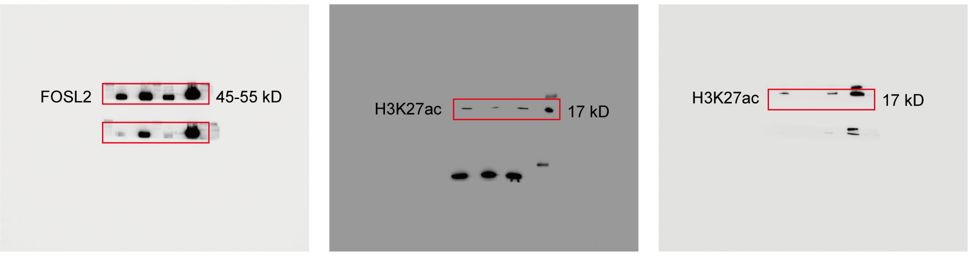

Supplementary Fig 9 A

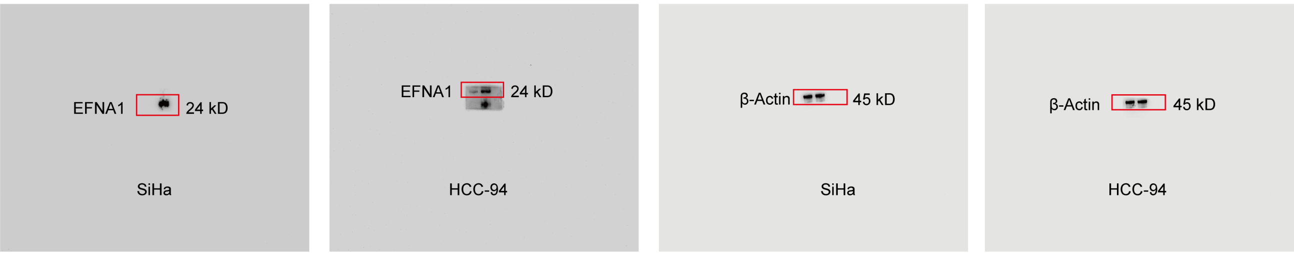

Supplementary Fig 13

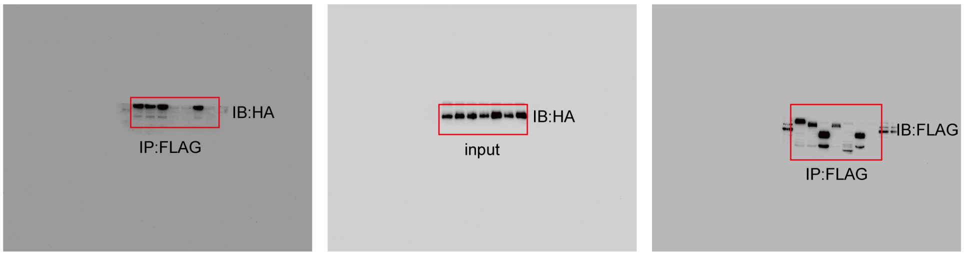

Supplementary Fig 14 A

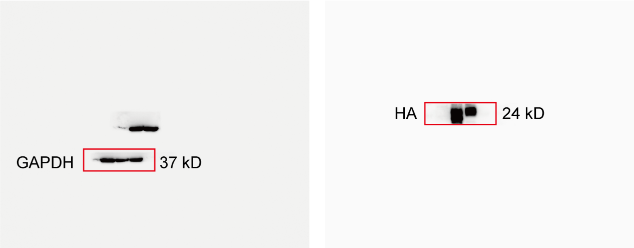

Supplementary Fig 14 B

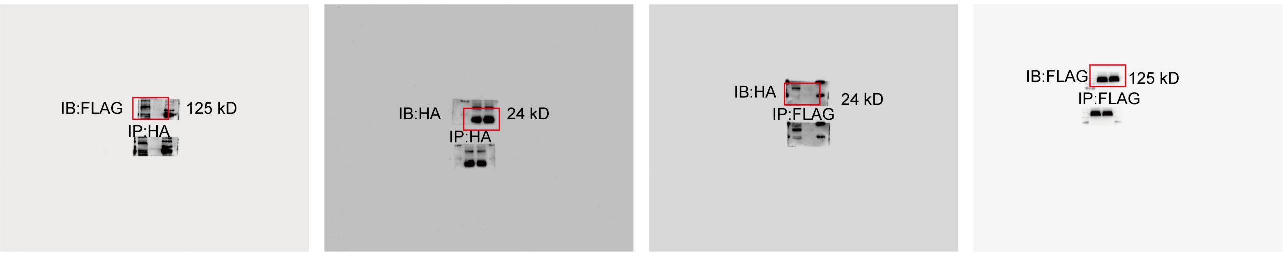

Supplementary Fig 14 C

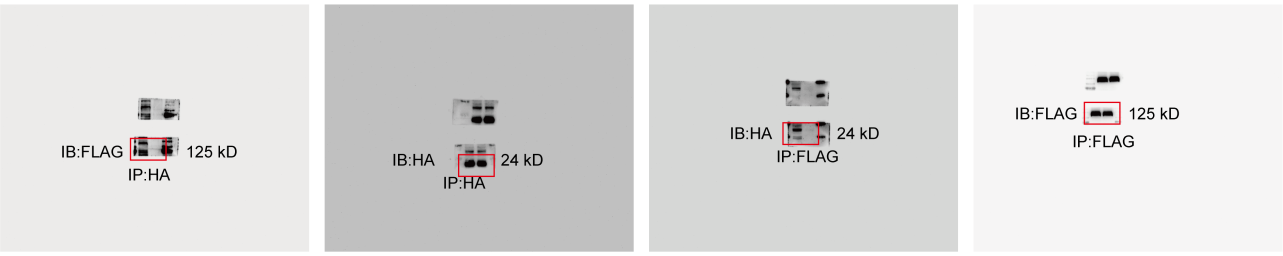

Supplementary Fig 15A

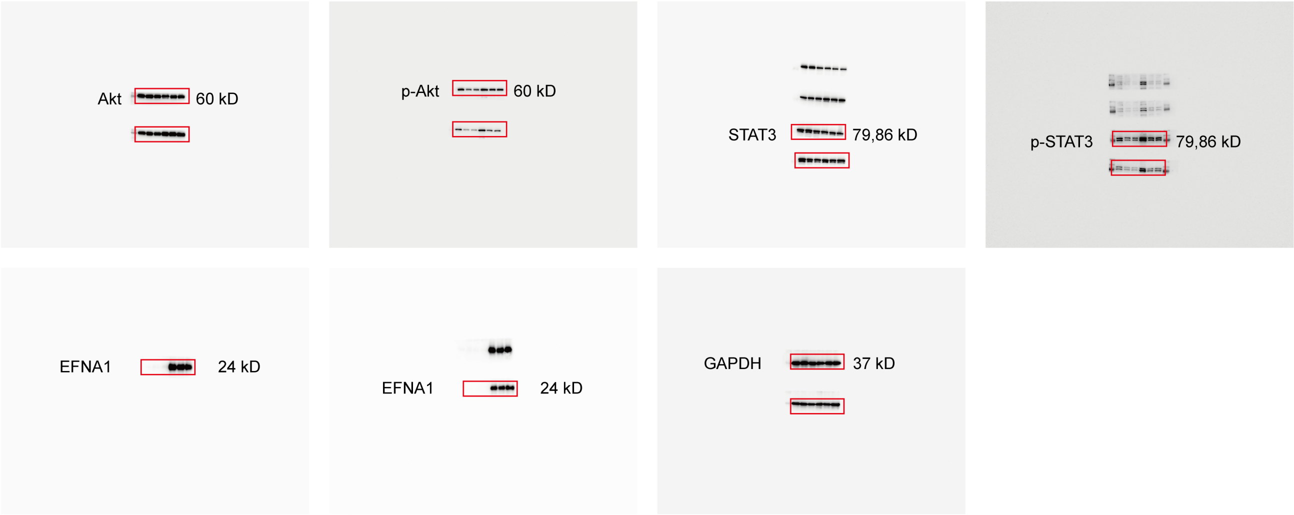

Supplementary Fig 16 A

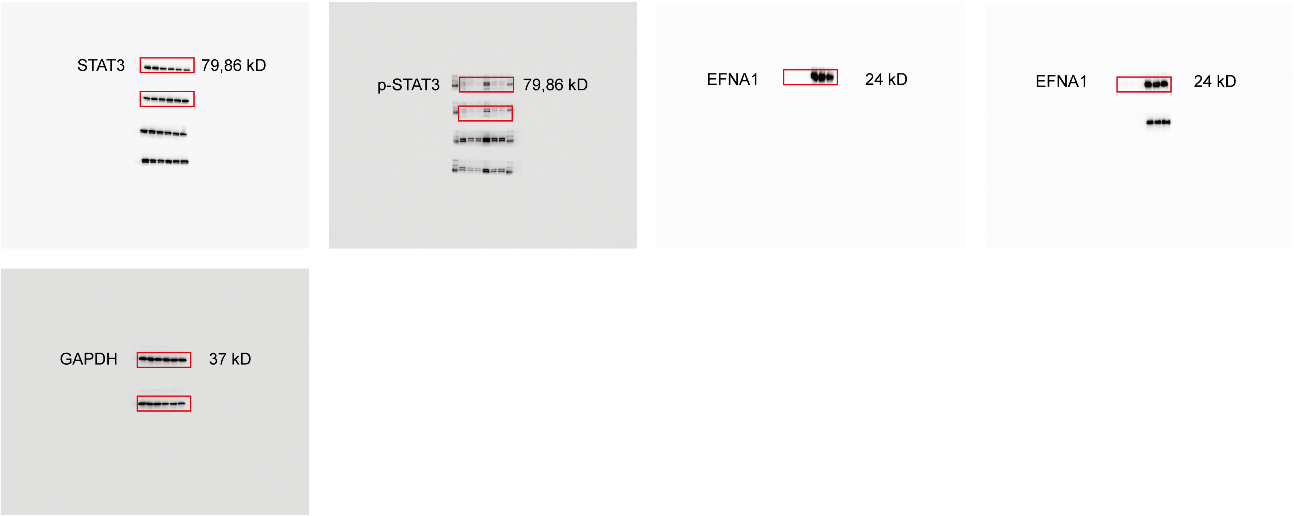

Supplement: Unedited blot and gel images [file jci-135-177599-s010.pdf]
